# Microbiological profile of patients with generalized gingivitis undergoing periodontal therapy and administration of *Bifidobacterium animalis* subsp. *lactis* HN019: A randomized clinical trial

**DOI:** 10.1371/journal.pone.0310529

**Published:** 2024-11-11

**Authors:** Flavia Furlaneto, Yara Loyanne de Almeida Silva Levi, Débora de Souza Ferreira Sávio, Izadora Cianfa Firmino da Silveira, Adriana Miranda de Oliveira, Talita Gomes Baêta Lourenço, Marcella Costa Ribeiro, Pedro Henrique Felix Silva, Sergio Luiz de Souza Salvador, Ana Paula Vieira Colombo, Michel Reis Messora

**Affiliations:** 1 Department of Oral & Maxillofacial Surgery and Periodontology, Ribeirao Preto School of Dentistry, University of Sao Paulo–USP, Ribeirao Preto, SP, Brazil; 2 Division of Post-graduate Periodontics, School of Dentistry, Federal University of Rio de Janeiro, Rio de Janeiro, RJ, Brazil; 3 Oral Microbiology Laboratory, Institute of Microbiology Paulo de Góes, Federal University of Rio de Janeiro, Rio de Janeiro, RJ, Brazil; 4 Department of Clinical Analyses, School of Pharmaceutical Sciences of Ribeirao Preto, University of Sao Paulo–USP, Ribeirao Preto, SP, Brazil; University of Catania: Universita degli Studi di Catania, ITALY

## Abstract

**Objective:**

To evaluate the adjunctive use of the probiotic *Bifidobacterium animalis* subsp. *lactis* HN019 (*B*. *lactis* HN019) to conventional therapy on clinical and microbiological parameters in patients with generalized gingivitis.

**Methods:**

Sixty systemically healthy individuals with untreated generalized gingivitis were submitted to periodontal therapy and allocated to receive Placebo (*n* = 30) or Probiotic (*n* = 30) lozenges, twice a day for 8 weeks. Bleeding on Marginal Probing (BOMP) was evaluated at baseline, after 2 and 8 weeks. Supra and subgingival biofilm were obtained at baseline and 8 weeks post-therapy for analyses by 16S rRNA gene sequencing. Differences between therapeutic groups were analyzed by non-parametric tests (*p*<0.05).

**Results:**

The Placebo and Probiotic groups showed a significant reduction in BOMP at 8 weeks compared to baseline (p<0.05). The Probiotic group had a lower percentage of BOMP when compared with the Placebo group at 8 weeks (*p*<0.0001). Alpha and beta-diversity showed no statistical significance between groups and time points. At phylum level, no significant differences were observed between groups and time points. At genus level, an increase in the relative abundances of *Bergeyella* and *Corynebacterium* were significantly associated with a greater reduction in bleeding in the Placebo group and with less reduction in bleeding in the Probiotic group, respectively. At species level, *Schaalia* spp., *Streptococcus gordonii*, and *Leptotrichia wadei* increased in Placebo and decreased in the Probiotic group after treatment. *Granulicatella adiacens* decreased significantly after the probiotic therapy, while *Saccharibacteria* (TM7) spp., *Solobacterium moorei*, and *Catonella morbi* increased significantly. In the Placebo group, *Bergeyella* sp. HMT-322 was associated with a greater percentage of reduction in bleeding. In both groups, *Actinomyces* species were related to less reduction in bleeding.

**Conclusion:**

The adjuvant use of *B*. *lactis* HN019 alongside conventional therapy enhanced the reduction in BOMP and promoted greater changes in the microbiological profile of patients with generalized gingivitis.

**Trial registration:**

The study was registered at Brazilian Clinical Trials Registry (ReBEC; protocol number: RBR-59v2yb).

## Introduction

The accumulation of biofilm and its microbial profile are the main etiologic factors of gingivitis [1]. Studies on self-assessment of periodontal conditions demonstrated that patients can underestimate the milder forms of gingival diseases, which consequently may affect the adequate control of the biofilm [2]. Moreover, maintaining a high level of personal oral hygiene tends to decrease over time despite the reinforcement and motivation by the professional [3].

In susceptible individuals, gingivitis can progress to periodontitis, causing irreversible destruction of periodontal tissues [4]. Since the success of periodontal treatment depends on the effectiveness of the biofilm control to eliminate or reduce periodontal pathogens, this may not be fully achieved by conventional strategies of mechanical removal of dental biofilm and regular patient self-care, and microbial recolonization may occur [5, 6]. Therefore, adjunctive agents may offer additional benefits in biofilm control in the management of gingivitis [7]. Different strategies of oral hygiene and the use of adjuvant therapies for controlling the dental biofilm have variable impacts on its microbial profile, which directly interfere with the clinical response to treatment, development, and reversal of gingivitis [8]. In this sense, there has been a wide search for alternative active agents that can be used to manage gingivitis [4].

In recent years, the scientific community has seen an awakening interest in the use of probiotics for oral health. These live microorganisms can confer health benefits on the host when administered in adequate amounts [9]. Clinical studies that evaluated the effects of probiotics on gingivitis showed conflicting results, as some reported improvement in gingival clinical parameters, reduction of periodontal pathogens, reduction of inflammatory markers in gingival crevicular fluid (GCF) and/or in saliva, and inhibition of gingivitis development [10–15]. In contrast, results from other studies did not reveal any additional benefits on the plaque, parameters of gingival inflammation, or profiles of salivary microbiome [16, 17]. Therefore, systematic reviews and meta-analyses show diversified results regarding the effects of probiotics on gingivitis [18, 19]. Also, a comprehensive understanding of the mechanisms of action of probiotics in gingivitis does not exist. It is worth noting that the impact of probiotics can differ based on various factors such as the specific type of strain or combination of strains used, the amount utilized, the timing of intervention, the method of delivery, and the existing microbiome of the individual [20].

Previous randomized clinical trials of our research group demonstrated that the association of the probiotic strain *Bifidobacterium animalis* subsp. *lactis* HN019 (*B*. *lactis* HN019) to non-surgical periodontal treatment in patients with generalized periodontitis provided additional benefits in terms of clinical, immunological, and microbiological outcomes [21, 22]. The probiotic group presented a greater reduction of microbial species of the red and orange complexes (*Porphyromonas gingivalis*, *Treponema denticola*, *Fusobacterium nucleatum vincentii*, *Campylobacter showae*, and *Eubacterium nodatum*), accompanied by larger counts of microbial species compatible with health compared with the control group [21]. More recently, we evaluated the effects of *B*. *lactis* HN019 as an adjunct to the conventional treatment of generalized gingivitis. The probiotic group presented reduced bleeding on probing and levels of interleukin (IL)-1β, IL-1α, and Monocyte Chemoattractant Protein (MCP)-1 in the GCF when compared with the Control group [23]. Considering the antimicrobial effects of probiotics on the management of gingivitis, most studies with microbiological analyses used culture methods, Real-Time Polymerase Chain Reaction (q-PCR), and DNA-DNA hybridization, which demonstrated no significant differences between the probiotic and the control groups in the counts of *Aggregatibacter actinomycetemcomitans*, *Porphyromonas gingivalis*, *Prevotella intermedia*, and *Fusobacterium nucleatum* [12, 16, 24]. In the last decade, the development and improvement of high-throughput sequencing technologies led to a better characterization of the oral microbiota in health and disease states, or in response to therapies in a complete way and with an unprecedented resolution, thus helping to understand that disease states are associated with changes in the balance of the entire microbiota, not just the presence of key pathogens [25–28]. These open-ended technologies allow the identification of unique microbial signatures in a variety of subjects, which can be valuable in evaluating the microbiological impacts of periodontal therapy and may provide a better understanding of the mechanisms of action of probiotics in modulating the microbiota and in the management of gingivitis. In the only study to evaluate the microbiological effects of probiotics on experimental gingivitis using sequencing technologies, the use of lozenges containing *Lacticaseibacillus rhamnosus* PB01 DSM14870 and *Lactilactobacillus curvatus* EB10 DSM32307 during both the oral hygiene discontinuation and restitution periods contributed to the recovery of baseline supragingival microbiota after the experimental period [29]. To date, no study has evaluated the microbiological effects of *B*. *lactis* HN019 administration in the treatment of generalized gingivitis using sequencing technologies. In this context, this study aimed to evaluate the effects of oral administration of *B*. *lactis* HN019, as an adjunct to conventional periodontal therapy [23], on the oral microbial profile of patients with generalized gingivitis, using 16SrRNA gene sequencing.

## Materials and methods

### Patient population

This study is the second stage of the study previously published by our group [23]. The additional evaluations presented in this paper are the clinical monitoring of Bleeding on Marginal Probing (BOMP) and the microbiological analysis by 16S rRNA gene sequencing. The study is reported according to the CONSORT and SPIRIT statements (S1 and S2 Checklists). This was a double-blind, single-center, placebo-controlled, prospective, randomized clinical trial with two parallel arms and an allocation ratio of 1:1. The project was approved (#68692917.5.0000.5419) by the School of Dentistry of Ribeirao Preto–University of Sao Paulo (FORP/USP) Ethics Committee. The authors confirm that all ongoing and related trials for this intervention are registered. The study protocol for this trial is provided in S1 and S2 Appendices. Patients enrolled for dental treatment at FORP/USP were contacted by phone or message and further screened. Eligible participants were provided with complete information about the study’s purpose, potential risks, and benefits of their participation in the study. They were also required to sign consent forms indicating their understanding of the nature of the study (S3 and S4 Appendices).

The software Graphpad Statemate 2.0 (GraphPad Software, Inc., San Diego, CA, USA) was used to calculate the sample size. The aim was to ensure that there was an 80% power to recognize a significant difference of 10% in BOMP (δ) between the groups analyzed. This was done with a confidence interval of 95% (α = 0.05) and standard deviation (Σ) of 12.43% [30]. The calculation was based on the following formula: n = {2 [(Σ)2/(δ)2]} x (Zα + Zβ)2. As a result, 24 patients were required for each experimental group. To account for the possibility of some patients being lost to follow-up, 60 patients were recruited.

### Inclusion and exclusion criteria

The inclusion criteria were: untreated dental plaque-induced generalized gingivitis assessed by BOMP, in more than 30% of the sites, with probing depths ≤3 mm, without radiographic bone loss and detectable attachment loss due to periodontitis [31]; non-smokers; systemically healthy; ≥20 fully erupted permanent teeth, excluding third molars; willingness to adhere to the study protocol. The exclusion criteria included: pregnant or lactating women; medical conditions affecting the progression of periodontal diseases or the response to treatment; antimicrobial, probiotic, and/or anti-inflammatory therapy in the previous six months; history or presence of periodontitis; presence of non-plaque-induced gingival disease, known allergies; extensive prosthetic appliances; legal incapacity; periodontal therapy in the previous six months and need of prophylactic antibiotic therapy for routine dental procedures.

### Experimental design, allocation concealment, and treatment protocol

**Fig 1:**
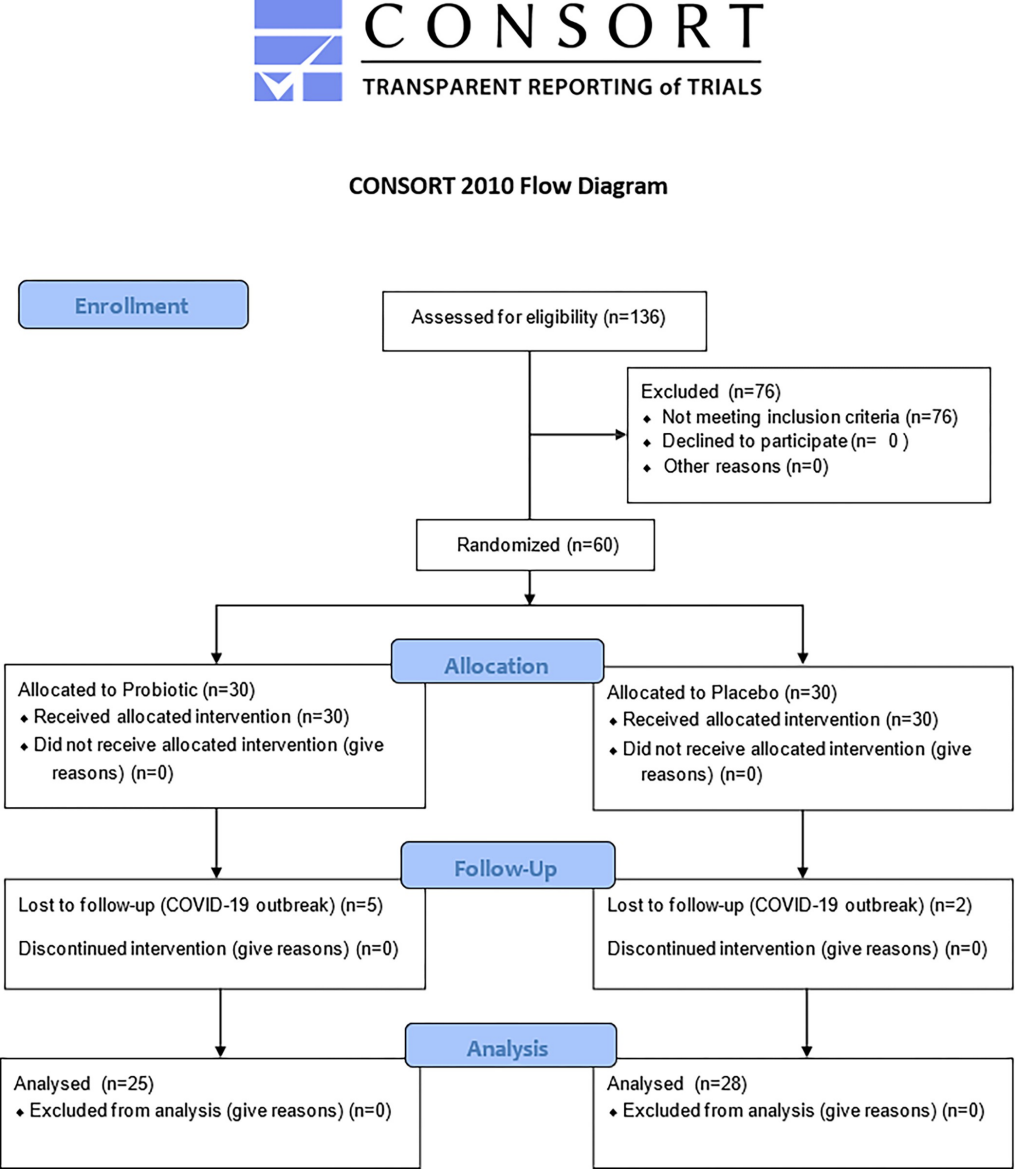
Flowchart of the study.

The recruitment of subjects was performed between May 2019 and March 2020. Each patient was followed up for 8 weeks from baseline. As depicted in Fig 1, eligible individuals were randomly allocated into the Probiotic (*n* = 30) or Placebo group (*n* = 30) after being identified by a numerical code, generated from computer software handled by the study coordinator (F.A.C.F.). The meaning of the codes was revealed by the study coordinator (F.A.C.F.) only after conducting the statistical analysis of the data.

The patients received probiotic lozenges containing 10^9^ colony-forming units (CFUs) of *B*. *lactis* HN019 (HOWARU® Bifido LYO 40 DCU-S, Danisco USA Inc., Madison, Wisconsin, USA) or placebo. A compounding pharmacy produced identical placebo and probiotics lozenges (i.e., the same taste, color, and appearance). Identical plastic vials containing the lozenges were sent to the study coordinator (F.A.C.F.), who wrote the numerical code of each patient on the vials. The coded vials were given to the examiner (M.C.R.), who was not informed of the content of the lozenges, and distributed them to the patients. The volunteers received 14 lozenges per week and were also blinded to the content of the lozenges. At the end of each week, they brought back the lozenges that had not been consumed during the week and then they received new lozenges. The patients received instructions not to consume other probiotic products during the study, to keep the lozenges in a refrigerator, and not to use any product for chemical control of bacterial plaque. One research assistant (P.H.F.S.) was responsible for monitoring patients’ compliance with the consumption of the lozenges and possible adverse events. At baseline, patients received periodontal therapy consisting of oral hygiene instructions, supragingival scaling using both hand (Gracey curettes; Hu-Friedy, Chicago, IL, USA) and ultrasonic instruments, and prophylaxis using a rubber cup and prophylaxis paste. These procedures were performed by one trained periodontist (G.A.S.) who was blinded to the experimental allocation. The patients were then instructed to take one lozenge twice a day (in the morning and before bedtime, after brushing teeth) for 8 weeks, containing either probiotic or placebo [23]. After all patients had completed the follow-up period, the study was ended.

### Clinical monitoring and biofilm samples for microbiological analysis

At baseline, as well as at 2 and 8 weeks, the BOMP was evaluated according to the angulated bleeding index (AngBI) [32] using a manual periodontal probe (PCPUNC156; Hu-Friedy, Chicago, IL, USA). The presence of bleeding was considered positive when occurring up to 30 seconds after insertion of the probe and was assessed at 4 sites per tooth (mesio-buccal, buccal, disto-buccal, and lingual). This step was performed by a trained and calibrated periodontist (Y.L.A.S.L.), who was blinded to the experimental groups. The intra-examiner reproducibility was determined by calibration evaluating ten patients (with both bleeding and non-bleeding sites upon probing) not related to this study, and the kappa coefficient was 93%. Each patient was evaluated on two separate occasions 48 hours apart to obtain the intra-examiner reliability [23].

For the collection of supra and subgingival biofilm samples, 8 interproximal non-contiguous sites with BOMP were selected. Samples were obtained from the same sites at baseline and after 8 weeks of treatment. Supra and subgingival biofilms were carefully removed with a sterile curette, from the most apical portion of the gingival sulcus to the coronal region, and transferred to sterile tubes containing 150uL of Tris-EDTA buffer. Subsequently, they were frozen in a -80°C freezer until analysis.

### Bacterial DNA extraction and 16S rRNA gene sequencing

Microbial DNA was obtained from 96 biofilm samples using a commercial QIAamp DNA mini Kit (QIAGEN, Düsseldorf, Germany). Three biofilm samples from the Placebo group and 7 from the Probiotic group were lost. The quality of the DNA was determined by electrophoresis in agarose gel and quantified by spectrophotometry (Nanodrop®, Thermo Fisher Scientific, São Paulo, SP, Brazil) and stored at -20°C until analysis. Next-generation sequencing targeting the V1-V2 hypervariable region of the 16S rRNA gene from the samples was carried out at NGS Soluções Genômicas (Piracicaba, SP, Brazil). The Illumina recommendations for library preparation were followed. A single-read sequencing of 250 nucleotides was performed using the MiSeq Illumina platform (Illumina, San Diego, CA, USA) 27F primer (S1 Text). Raw 16S rRNA sequence data were submitted to Sequence Read Archive (SRA) under BioProject accession number ID #PRJNA846723.

### Statistical analysis

The % of sites with BOMP was computed per participant and then averaged across patients in both groups. The within-group and between-group differences for BOMP were assessed by repeated-measures analysis of variance followed by Bonferroni *post-hoc* test and by Student’s t-test, respectively, with a significance level of 5% in all tests. For the microbiological data, rarefied amplicon sequence variants (ASVs) were used for computing diversity metrics. Relative abundance of assigned taxa was computed for each group and time-point, at various taxonomic levels. Taxa assigned to genus and/or species, at mean relative abundance of >1% and >0.5%, respectively, were selected to compute changes in abundance ($\%\Delta =\frac{T8-T0}{\left|T0\right|}\times 100$) after treatments. Associations and differences among groups for all parameters were assessed by Mann-Whitney and Kruskal-Wallis tests, whereas differences within groups were sought by the Wilcoxon signed-rank test. Bivariate correlation analyses between clinical and microbiological parameters were evaluated by Pearson and Spearman correlation coefficients. Given the high interdependence among oral species/phylotypes within the subgingival biofilm, multiple linear regression analysis was also carried out to search for microbial predictors (at species level) of improved therapeutic response. Changes in % of sites with BOMP post-therapy were computed for each group, and used as the clinical outcome parameter in the regression models. As predictors, 441 oral species were considered as potential independent variables in the regression using a stepwise method. The normal distribution of the dependent variable was confirmed by the Kolmogorov-Smirnov test. Assumptions of linearity (Pearson correlation), multicollinearity (tolerance and VIF), homoscedasticity and outliers were also checked within the regression analysis to select the best model. All analyses were performed by GraphPad Prism 6 (GraphPad Software, Inc., San Diego, CA, USA) and SPSS 21.0 (IBM São Paulo, SP, Brazil). All data were assessed by the study coordinator for the analyses (F.A.C.F.).

## Results

### Clinical monitoring

**Fig 2:**
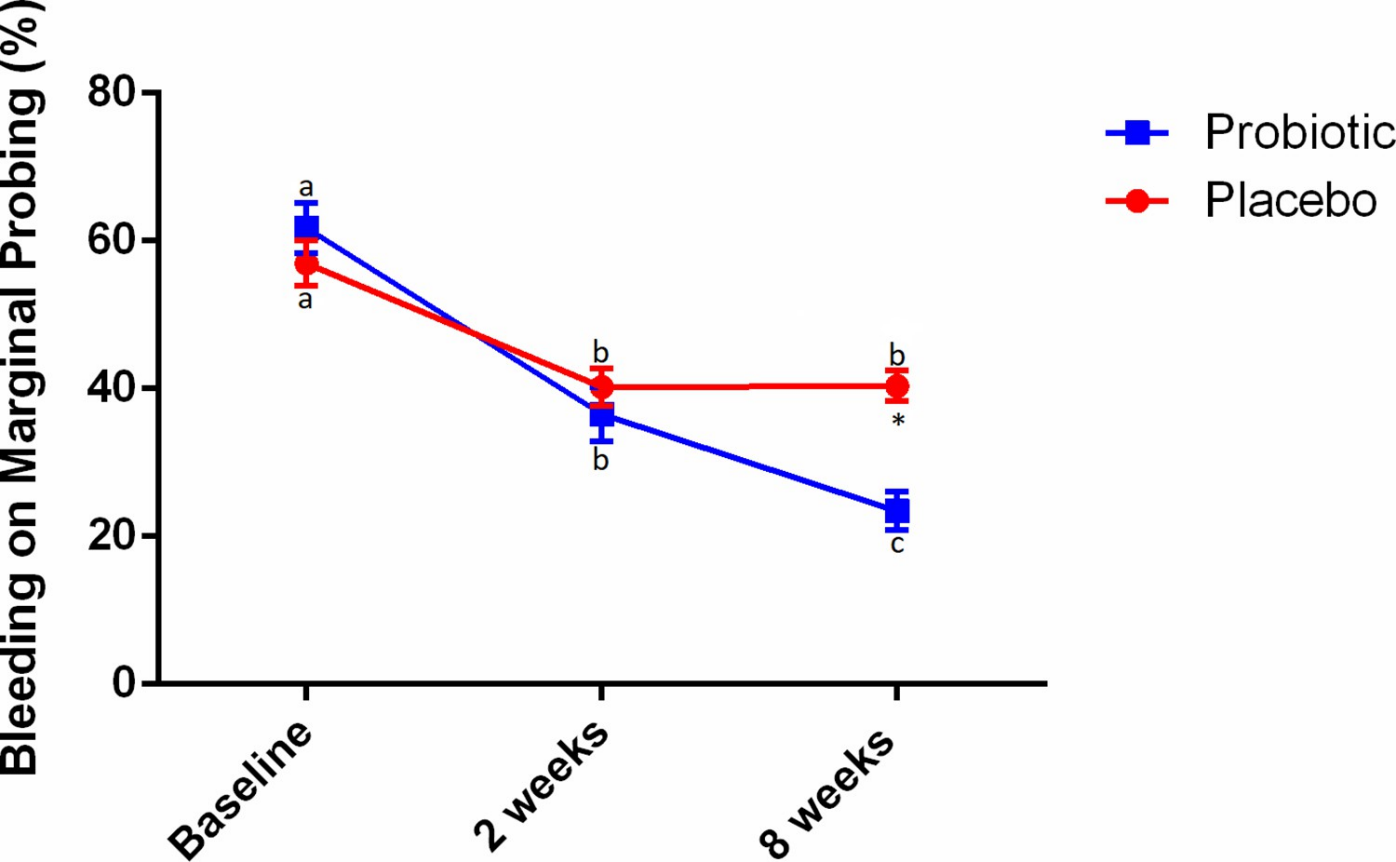
Bleeding on Marginal Probing (BOMP) scores in the test and control groups at baseline and after 8 weeks. Different letters indicate significant differences in intra-group comparisons (repeated measures ANOVA followed by Bonferroni post hoc test for multiple comparisons; p<0.05). *Significant difference in inter-group comparisons: 8-week values in the test group compared to 8-week values in the control group (p<0.0001).

The flowchart of the study is shown in Fig 1. The sample of this trial consisted of 28 patients in the Placebo group (18 female and 10 male) with mean age ± standard deviation (SD) of 22.89 ± 10.90 years and 25 patients in the Probiotic group (16 female and 9 male) with mean age ± SD of 23.92 ± 10.23 years. The demographic data of the study participants was previously described [23]. Two patients in the Placebo group and 5 patients in the Probiotic group were lost to follow-up due to COVID-19 outbreak. As previously reported, no significant differences between groups for clinical or demographic parameters were seen at baseline [23]. Throughout the study, there were no adverse effects observed as a result of probiotic therapy [23]. Fig 2 shows mean percentage rates and standard errors for BOMP over time. In the Probiotic group, significant reductions in BOMP were observed at both 2 (36.57 ± 18.30) and 8 (23.46 ± 12.89) weeks compared to baseline (61.74 ± 17.07), and also between 2 and 8 weeks (p<0.05). In the Placebo group, a significant reduction in BOMP was observed at both 2 (40.21 ± 13.66) and 8 (40.38 ± 10.73) weeks compared to baseline (56.97 ± 16.29) (p<0.05), however, no significant differences were observed between 2 and 8 weeks. Further, the Probiotic group presented lower BOMP at 8 weeks when compared to the Placebo group (p<0.0001).

### No changes on microbial diversity of the periodontal biofilm after therapies

**Fig 3:**
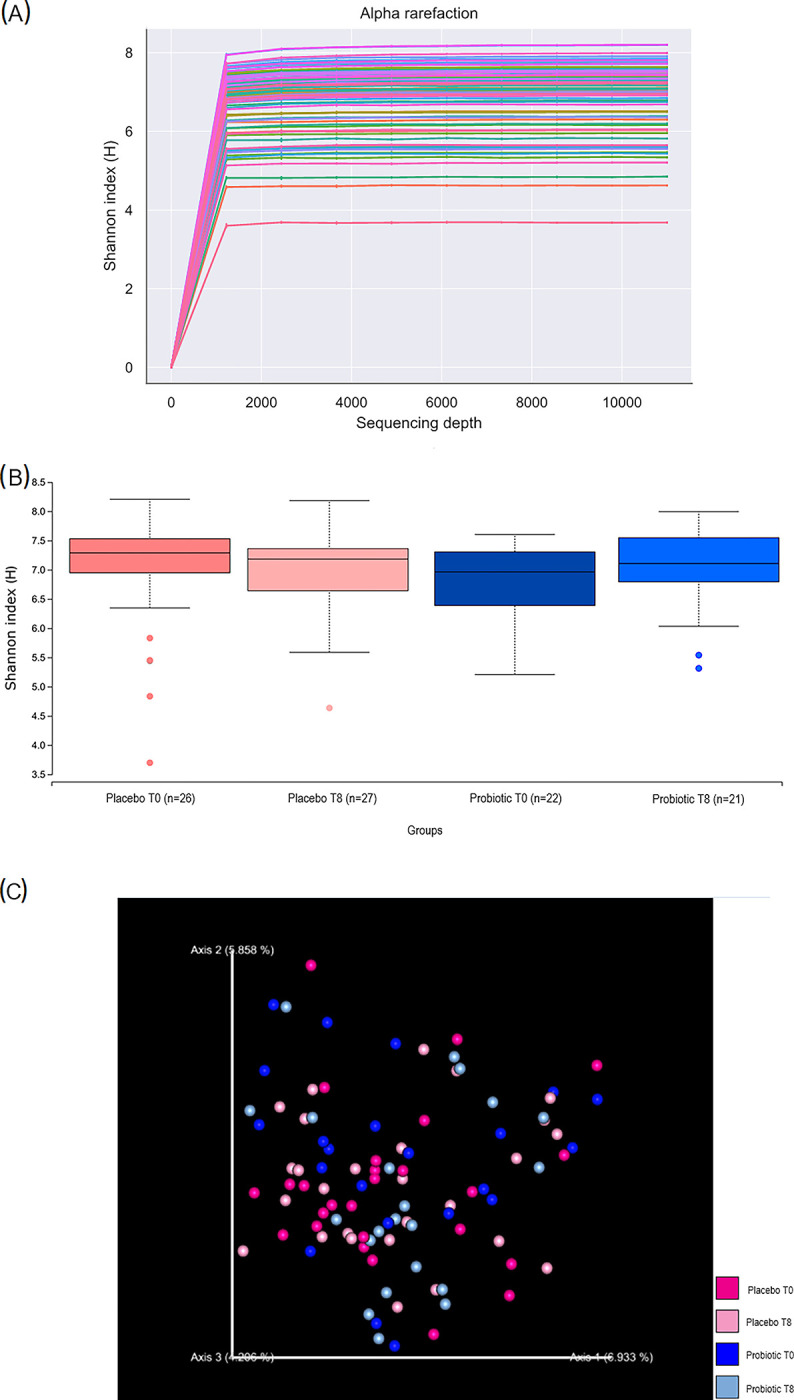
Alpha and beta-diversity. (a) Rarefaction analysis comparing diversity plaque samples across different treatment groups and time points. The curves represent the average number of ASVs encountered during iterative resampling of the original data. Shannon index provides inference about species richness and evenness based on relative abundances of different species. (b) Shannon entropy (H) was calculated for both treatment groups at baseline and post-therapy, no statistical significance was found between groups (Kruskall-Wallis, p>0.05). (c) Beta-diversity comparison among individuals by treatment group and time-point. PCoA plot based on Bray-Curtis dissimilarity metrics indicates no clear clustering of individuals based on treatment or time-point. T0 and T8 stands for baseline and post-therapy, respectively.

For diversity metrics calculation, a sampling depth of 11472 was used. Individual alpha rarefaction analysis showed that the zero slope was attained in the selected sampling depth (Fig 3A). Shannon entropy (H) was calculated for both treatment groups at baseline and post-therapy, but no statistical significance was found (p>0.05, Fig 3B). Beta-diversity analysis was done using Principal Coordinates Analysis (PCoA) ordination with Bray-Curtis dissimilarity, but no obvious clustering was observed within and between treatment groups (Fig 3C). The ASVs were then taxonomically assigned to species-level based on Human Oral Microbiome Database (eHOMD) (Chen, 2010) 16S rRNA RefSeq Version 15.22 using the sklearn-based taxonomy classifier algorithm.

### Changes in oral taxa abundance at phylum and genus levels

**Fig 4:**
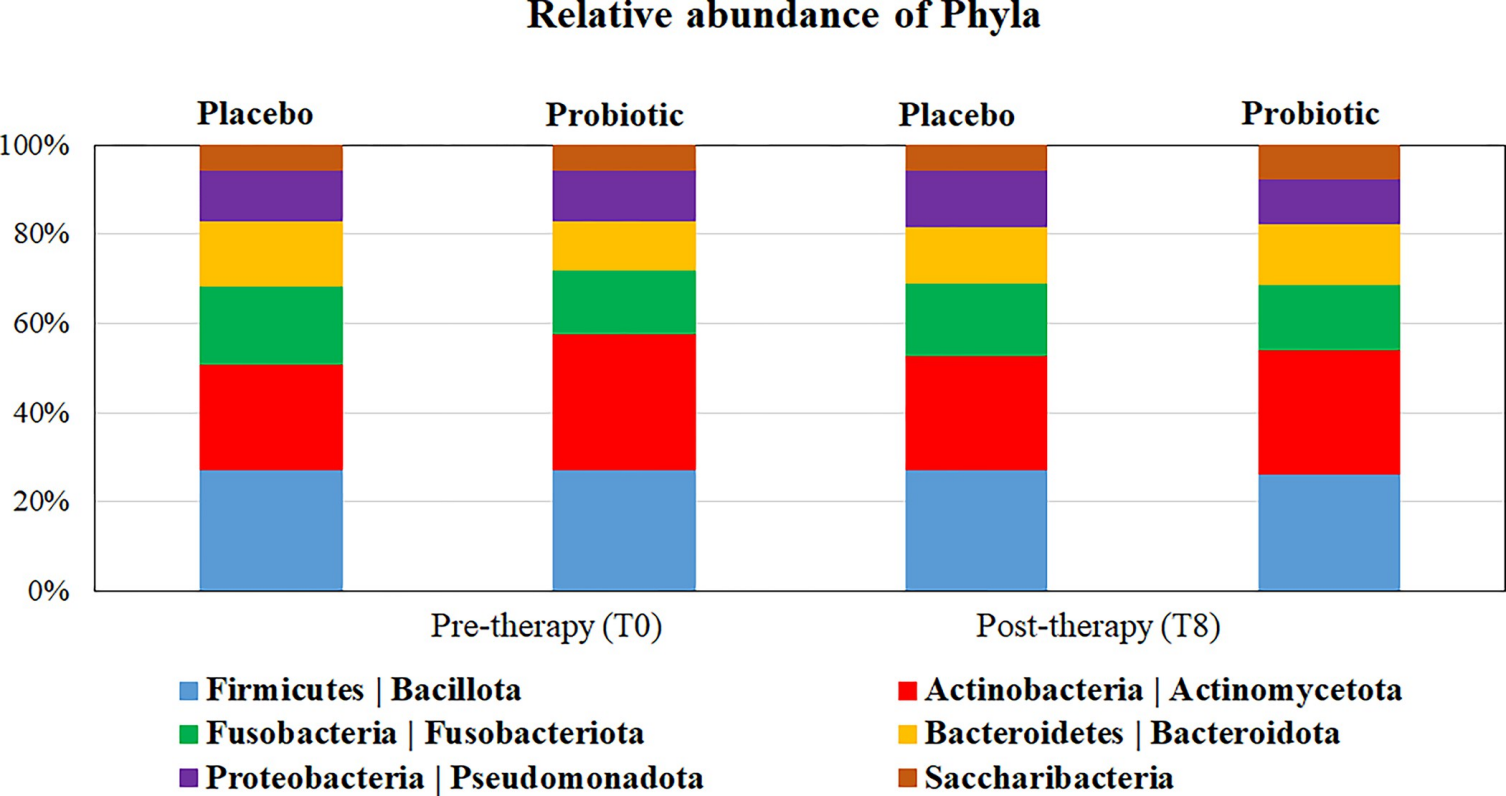
Distribution of predominant phyla in both therapeutic groups, before and after treatments. No significant differences in relative abundances between groups and time points were observed (Mann-Whitney and Wilcoxon sign tests, p > 0.05).

After taxonomy assignment, taxa at phylum and genus levels with abundance >1% were further analyzed. For the six most abundant phyla, no significant differences between groups and time points were observed (p>0.05, Fig 4). The most predominant genera were *Streptococcus*, *Actinomyces*, *Leptotrichia*, *Fusobacterium*, and *Prevotella*. Three low abundant genera (*Shuttleworthia*, *Lachnospiraceae*_[G-2] and _[G-7]) differed between groups at baseline (p<0.05). Among genera with an overall relative abundance ≥ 1% at baseline, *Schaalia* was the only genus that increased significantly in the Placebo group after treatment. Significant changes were also observed for low abundant taxa: *Granulicatella* reduced, and *Saccharibacteria* (TM7) [G-2] and *Catonella* increased in abundance after treatment with Probiotic, whereas *Tannerella* reduced in the Placebo group. At 8 weeks post-therapy, *Peptostreptococcaceae* [XI][G-7], *Peptostreptococcus*, and *Propionibacterium* were significantly more abundant in the Probiotic compared with the Placebo group (p<0.05; S1 Table).

**Fig 5:**
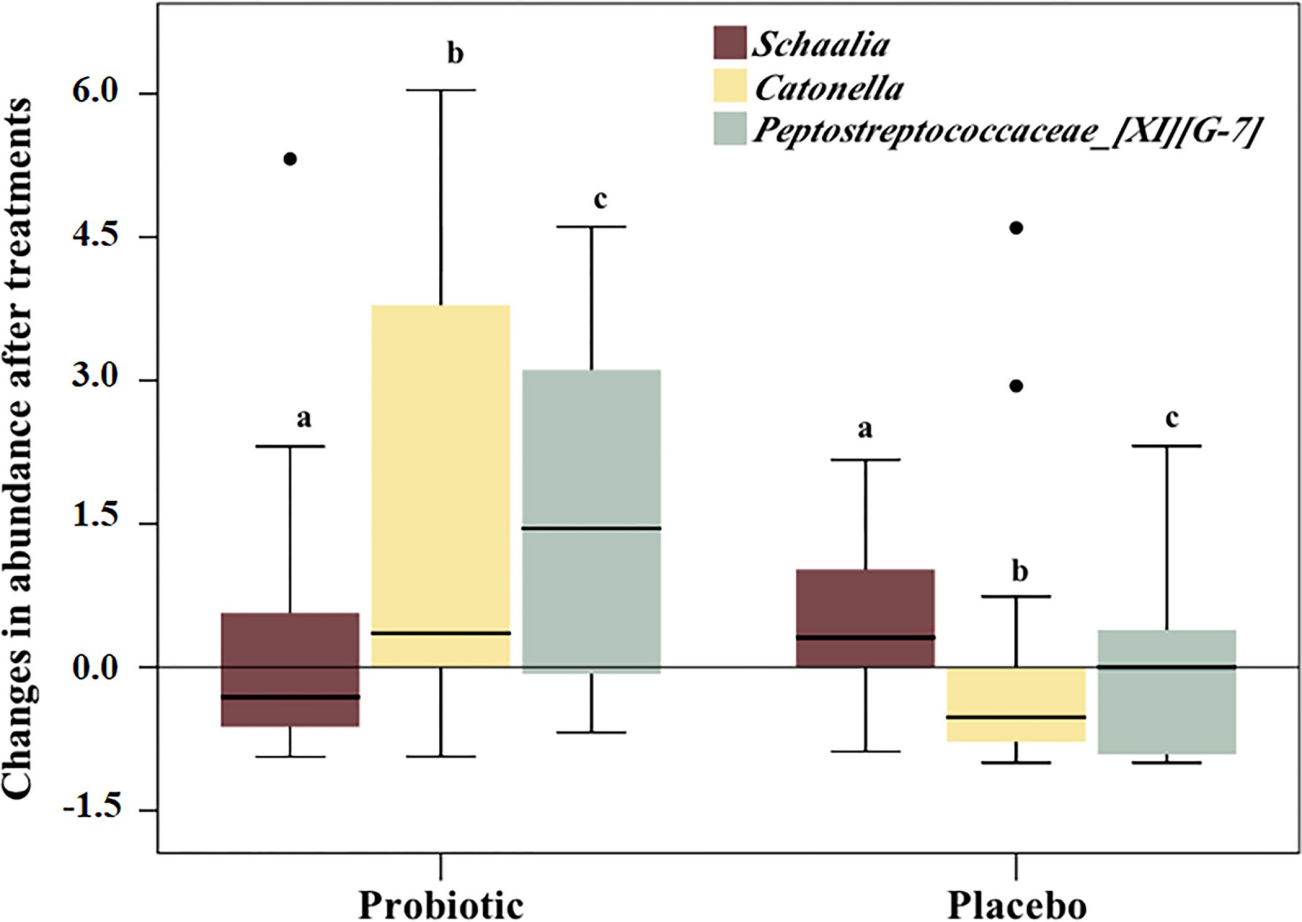
Changes in relative abundance of genera in both therapeutic groups. Box plots represent the 3 genera that differed significantly between groups in terms of changes in abundance. Negative values refer to reduction in abundance after treatment (Mann-Whitney test, a, p = 0.049; b, p = 0.001; c, p = 0.006). For instance, *Catonella* had a median increase of 35.5% in the probiotic group compared to a median reduction of 52% in the placebo. In contrast, *Schaalia* increased in the placebo and decreased in the probiotic group by 31%.

When changes in genus abundance over time were compared between treatments, 3 taxa presented significant differences (Fig 5). *Catonella* had a median increase of 35.5% in the Probiotic group compared with a median reduction of 52% in the Placebo (p = 0.001). In contrast, *Schaalia* increased in the Placebo and decreased in the Probiotic group by 31% (p<0.05), whereas *Peptostreptococcaceae* [XI][G-7] showed > 100% increase in the Probiotic group, but did not change in median abundance in the Placebo (p = 0.006).

**Fig 6:**
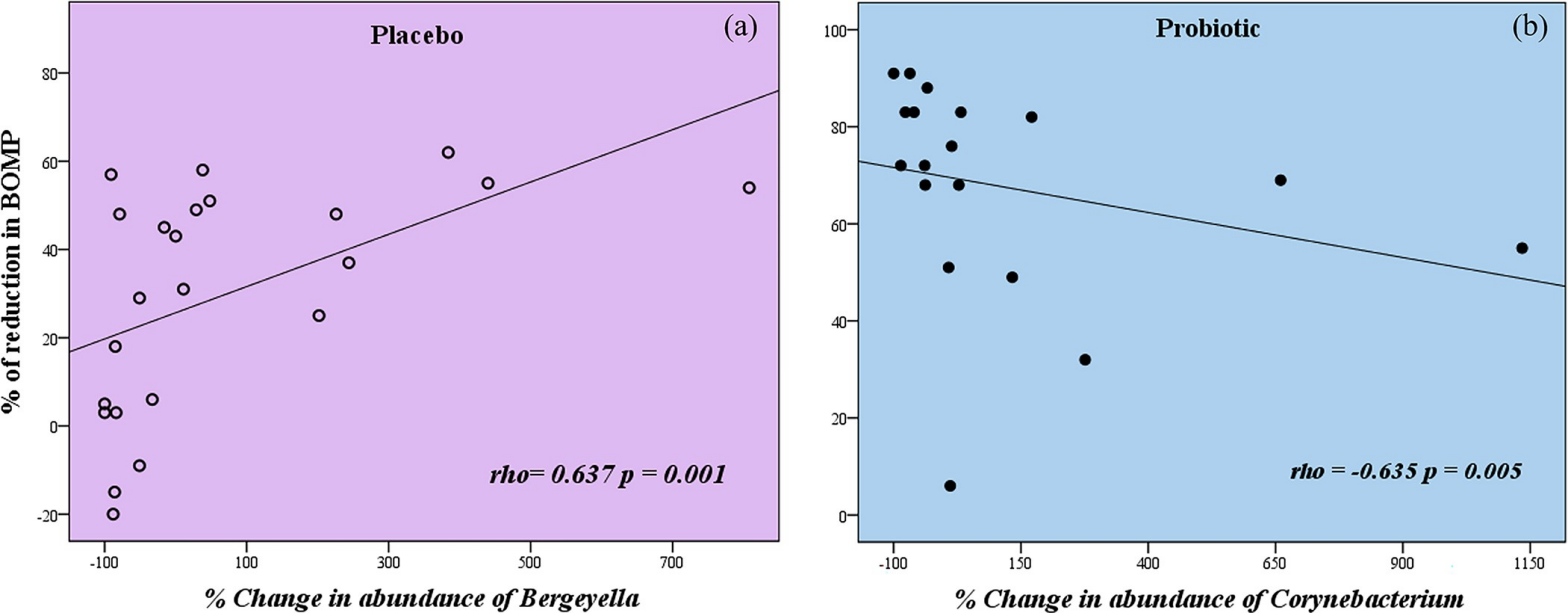
Spearman correlation (rho) between % of reduction of sites with bleeding on marginal probing (BOMP) and % change in relative abundance of specific genera 8 weeks after treatment in the groups. (a) Increase in relative abundance of *Bergeyella* is significantly associated with greater reduction in BOMP post-therapy in the Placebo group. (b) Increase in abundance of *Corynebacterium* is significantly associated with less reduction in BOMP post-therapy in the Probiotic group.

Changes in the mean % of sites with BOMP after therapy were correlated with changes in the relative abundance at 8 weeks of all genera detected. Considering only robust and highly significant correlations, an increase in abundance of *Bergeyella* correlated with a greater reduction of sites with BOMP after therapy with Placebo. In contrast, an increase in the abundance of *Corynebacterium* correlated with less reduction in BOMP after therapy with Probiotic (Fig 6 and S2 Table, p<0.01 and p<0.05).

### Changes in abundance of oral species and correlation with clinical outcome

**Fig 7:**
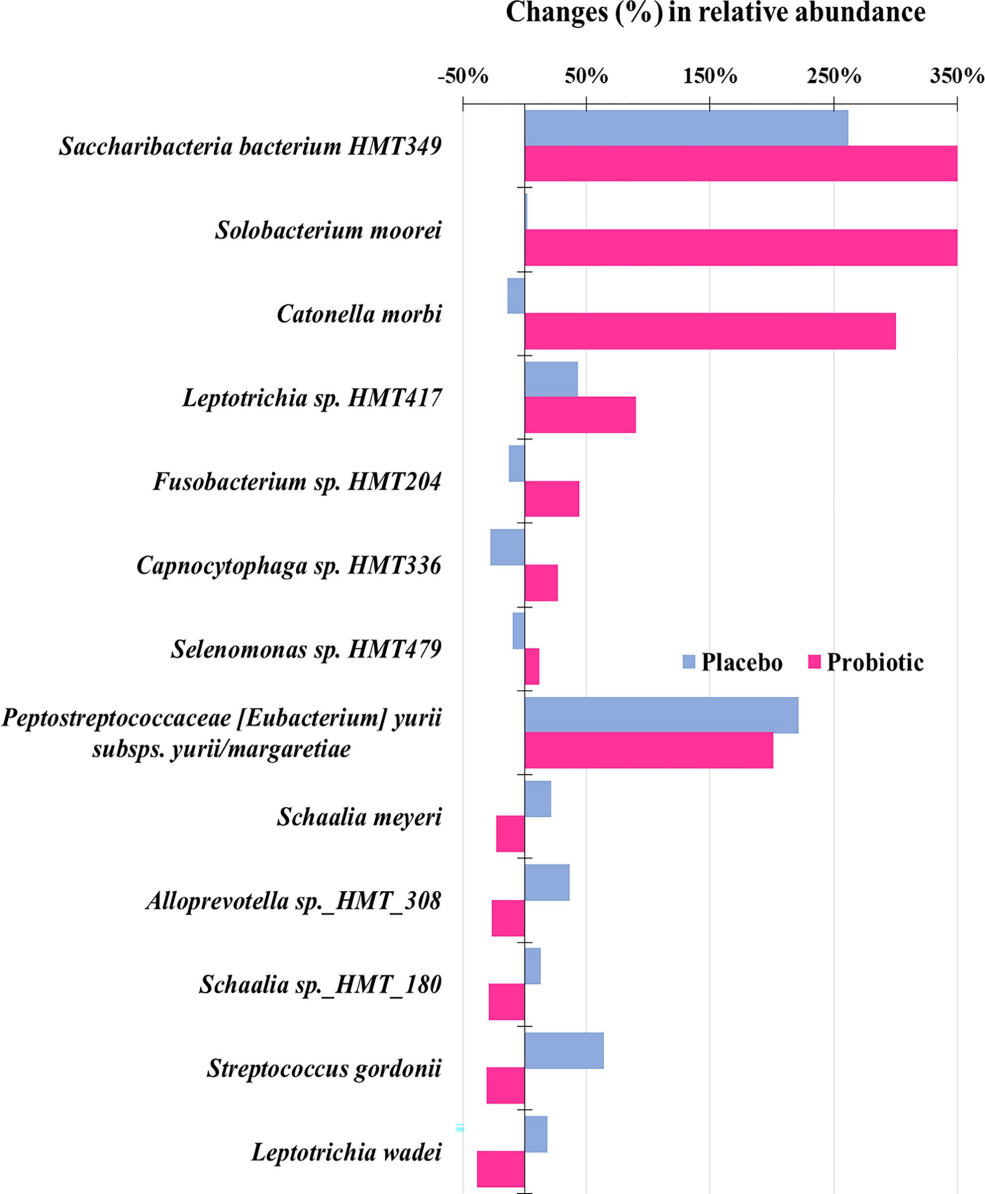
Changes in abundance of oral taxa at species level in both therapeutic groups. All taxa depicted differed significantly between groups in % of abundance changes. The top 3 species showed a marked increase in abundance after therapy with Probiotic compared to Placebo (Mann-Whitney test, p < 0.05).

Few species differed in abundance between groups at baseline or at 8 weeks after therapy (p<0.05, S3 Table). *Capnocytophaga gingivalis* increased, while *Catonella morbi* reduced significantly in the Placebo group (p<0.05). Changes were more preeminent in the Probiotic group, with 8 taxa showing an increase and only 3 presenting a reduction in abundance after treatment, which are *Granulicatella adiacens*, *Schaalia sp*. HMT-180 and *Streptococcus intermedius* (p<0.05, S3 Table). Among these taxa, species of *Capnocytophaga*, *Saccharibacteria* (TM7), *Prevotella* sp. HMT-472 and *Peptostreptococcaceae* [XI][G-7] [*Eubacterium*] *yurii subsps*. *yurii margaretiae* increased in both groups, but statistical significance was computed just for the Probiotic group. Shifts in the composition of the gingival biofilm after therapies were also evaluated by comparing the percentage of changes in oral species that had ≥0.1% of abundance at baseline (Fig 7). Of over 100 taxa, approximately 10% showed significant differences between therapies in the % of changes (increase or decrease). Overall it is noted that the Probiotic group had greater changes in taxa relative abundance; changes in the Placebo group were found, however they were less notable. Marked increases were observed in the Probiotic group for *Solobacterium moorei*, *C*. *morbi*, and *Saccharibacteria bacterium* HMT-349 (p<0.05). In contrast, *S*. *gordonii*, *Leptotrichia wadei*, *Alloprevotella* sp. HMT-308, *Schaalia meyeri* and *Schaalia* sp. HMT-180 reduced in the Probiotic group but increased in the Placebo, although at much lower proportions (p < 0.05).

Cross-analysis between species abundance and gingival bleeding was performed using Spearman correlation coefficient and regression analysis. In the probiotic-treated individuals, the increase in abundance of several species correlated with a greater reduction in gingival bleeding; however, increased abundance of *L*. *wadei* and *Leptotrichia* HMT-223 were associated with less reduction in % of sites with BOMP post-therapy (rho > 0.500, *p*<0.05, Table 1). Of interest, the enrichment of biofilm with four species/phylotypes of *Actinomyces* was moderately related to less reduction of BOMP in both groups (rho > 0.500, p<0.05). In the Placebo group, increase in *Bergeyella* HMT-322 abundance correlated with less bleeding post-therapy (rho = 0.578, *p*<0.01, Table 1).

**Table 1:** Correlation between the % of reduction in bleeding on marginal probing (BOMP) and changes in the abundance of oral species over time in the therapeutic groups.

Changes in relative abundance post-therapy (T8)	% of reduction in BOMP
**Placebo**	
*Actinomyces massiliensis*	-0.500*
*Actinomyces oris*	-0.527*
*Bergeyella* HMT-322	0.578**
*Prevotella salivae*	-0.488*
*Capnocytophaga sp*. HMT-336	0.485*
*Capnocytophaga sp*. HMT-335	0.449*
**Probiotic**	
*Actinomyces* HMT-170	-0.714**
*Actinomyces* HMT-175	-0.867**
*Bacteroidetes bacterium* HMT-365	0.644**
*Bacteroidetes bacterium* HMT-505	0.482*
*Desulfobulbus* HMT-041	0.505*
*Escherichia coli*	0.698**
*Fretibacterium* HMT-358	0.698**
*Fusobacterium naviforme*	0.556*
*Fusobacterium* HMT-203	0.475*
*Leptotrichia* HMT-223	-0.584*
*Leptotrichia wadei*	-0.523*
*Pseudoramibacter alactolyticus*	0.698**
*Peptostreptococcaceae* [XI][G-5]*[Eubacterium] saphenum*	0.602**
*Peptostreptococcaceae* [XI][G-7]*[Eubacterium] yurii subsp*. *schtitka*	0.680**
*Prevotella baroniae*	0.525*
*Prevotella dentalis*	0.698**
*Prevotella enoeca*	0.698**
*Prevotella* HMT-443	0.505*
*Streptococcus cristatus clade-578*	0.586*
*Streptococcus oralis subsp*.*dentisani clade-058*	0.637**
*Streptococcus gordonii*	0.470*
*Saccharibacteria bacterium* HMT-955	0.477*
*Treponema* HMT-258	0.505*
*Treponema* HMT-268	0.554*
*Treponema* HMT-517	0.698**
*Veillonellaceae bacterium* HMT-135	0.505*

Values represent the Spearman correlation coefficient (rho). Species in green are related to better clinical outcome; i.e. higher reductions in bleeding are correlated with higher abundances of these taxa, whereas increased abundance of species in light orange are associated with more gingival inflammation

(* p < 0.05

** p < 0.01, Mann-Whitney test). T8: 8 weeks after treatments.

Next, associations between clinical and microbial parameters were examined by constructing multiple linear regression models in which over 400 oral species were selected as predictors of therapeutic response (change in BOMP post-therapy, *i*. *e*., reduction in gingival bleeding) in each group (S4 and S5 Tables). In the Placebo group (S4 Table), the best model that met all the assumptions was able to significantly explain over 90% of the variance in BOMP with six oral species/phylotypes as predictors [F (6, 16) = 34.194; p < 0.001; R^2^ = 0.928]. High abundance of *Bergeyella* sp. HMT-322 (β = 0.503, t = 6.72, p < 0.001) and *Schaalia* sp. HMT-178 (β = 0.355, t = 65.05, p < 0.001), and low abundance of *Treponema* sp. HMT-231 (β = -0.460, t = -6.39, p < 0.001) were the best predictors of high reduction in gingival bleeding. For reduction in bleeding after treatment of gingivitis with probiotic (S5 Table), the regression analysis also resulted in a statistically significant model [F (6, 11) = 109.178; p < 0.001; R2 = 0.983], with decreased abundance of *Actinomyces* sp._HMT-175 (β = -0.910, t = -22.88, p < 0.001) and *Leptotrichia* _HMT-498 (β = -0.422, t = -10.21, p < 0.001) as the best predictors of high reduction in gingival bleeding.

## Discussion

Diagnosis and treatment of gingivitis are important to prevent the development of periodontitis in susceptible individuals and subsequently to promote overall health, as periodontitis is associated not only with oral effects but also with systemic effects, such as increased inflammatory burden, decreased serum total antioxidant level and increased risk of oxidative stress, which may contribute to the severity and progression of other diseases [33–35]. In addition, gingivitis can be associated with negative effects such as pain, discomfort, and aesthetic issues that affect the quality of life, and treatment can play an important role in reducing these negative effects [36, 37].

This double-blind randomized controlled trial was conducted to assess the efficacy of oral probiotic *B*. *lactis* HN019 as an adjunct to the treatment of generalized gingivitis by investigating the changes in BOMP and supra and subgingival microbial profiles. In this study, both Placebo and Probiotic groups showed clinical improvements and microbiological changes after periodontal therapy, but the use of lozenges containing the probiotic promoted a greater reduction in gingival bleeding and a distinct microbial profile of the biofilm at 8 weeks of post-therapy evaluation. The use of probiotics in the treatment of plaque-induced gingivitis and periodontitis has been extensively investigated in recent years [19, 21–23, 38]. Some probiotic strains have demonstrated marked reductions in BoP, PI and GI scores, highlighting their potential benefits in the management of gingivitis [9, 23, 24, 39].

Patients with gingivitis have been shown to harbor an intermediate microbial profile between gingival health and periodontitis [26, 40]. Regarding the baseline microbial composition of the gingivitis-associated biofilm, our data on the relative abundance of bacterial taxa at phylum, genus, and species levels showed that both groups had similar microbial compositions at baseline, consistent with gingivitis [1]. However, clustering comparisons indicated great inter-individual variability in both groups and experimental periods, with relatively few taxa shared by the majority of individuals, which is in agreement with previous reports [25, 41, 42]. Although no significant intra- and inter-group differences were noted in alpha and beta diversities, there were changes in the abundance of genera and species associated with periodontal health or disease after the probiotic intervention. Among over 100 genera and almost 200 species/phylotypes detected, few taxa changed significantly in abundance after both therapies. These changes were quite different between groups, indicating distinct effects on the microbiota. In general, the adjunctive use of probiotics had a larger impact on several oral taxa at 8 weeks post-therapy, which may explain in part the better clinical outcome (greater reduction in BOMP) found in this group in comparison to controls. Significant reductions in *G*. *adiacens*, *Schaalia* spp., *L*. *wadei*, *Streptococcus gordonii*, *Streptococcus intermedius*, and *Alloprevotella* sp._HMT-308 were seen, indicating a potential antimicrobial activity of *B*. *lactis* HN019 against these species. *S*. *gordonii* has been shown to facilitate colonization by *P*. *gingivalis* and to increase the virulence of *Aggregatibacter actinomycetemcomitans* [28, 43], while *L*. *wadei* was found in high frequency in samples from periodontal pockets of Iranian patients [44].

High post-therapy abundance of *L*. *wadei* and *Leptotrichia* phylotypes HMT-223 and HMT-498 had a positive correlation with more gingival inflammation in the Probiotic group, corroborating data presented by Abusleme et al. (2021) [26]. Species of *Leptotrichia* are commensal oral bacteria that act as opportunistic pathogens and they have been found to play a role in a wide range of infections and immune-inflammatory responses [44–46]. Closely related to the family *Fusobacteriaceae*, this genus was among the top five more abundant ones in this gingivitis population sample, before and after therapies. However, one should consider that different strains do present variable virulence. Therefore, it is important to evaluate taxa at the strain/species level, as there are species associated with health or disease belonging to the same phylum and/or genus [42]. *G*. *adiacens*, another gingivitis-related species [26], is a nutritionally variant streptococci of the oral microbiota also involved in dental abscesses and infectious endocarditis [47–49]. Although this species was diminished after the probiotic therapy, it was not affected in the placebo.

*Schaalia*, in particular *S*. *meyeri* and the taxon *Schaalia* sp. HMT-180, was adversely impacted by therapies, being one of the few genera that increased significantly in the Placebo and diminished modestly in the Probiotic group. In addition, the high abundance of the taxon HMT-178 was a good predictor of clinical improvement in the placebo. Bacteria of the genus *Actinomyces* were recently moved to the genus *Schaalia* [50]. These species are early colonizers of the dental biofilm, abundant in the healthy mouth, particularly on the oral mucosa. They have also been isolated from advanced carious lesions and endodontic infections [51, 52].

Enrichment of specific taxa in the biofilm after probiotic therapy included *P*. *catoniae*, *Prevotella* sp. HMT-472, *Saccharibacteria* (TM7) spp., *S*. *moorei* and *C*. *morbi*. These changes were significantly correlated with an improvement in gingival bleeding. In contrast to our findings, *C*. *morbi* was considered a potential periodontitis-related species [53–55], also enriched in gingivitis [1, 26]. *S*. *moorei* was related to periodontitis-associated halitosis [56], whereas several *Saccharibacteria bacterium* phylotypes were shown to be more enriched in periodontitis than health and gingivitis sites by other authors [26]. Methodological differences among studies and distinct populations may account in part for these controversial data.

Increased abundance of the genus *Corynebacterium* was also strongly associated with less reduction in BOMP in the probiotic group, even though this genus did not change markedly from pre- to post-therapy in both groups. *Corynebacterium* appears as a key forming element during early biofilm formation [57]. The increase in *Corynebacterium* abundance may have contributed to more biofilm formation and therefore more gingival bleeding. On the other hand, investigators also showed that this genus is enriched in periodontal health compared to gingivitis and periodontitis [26, 58]. In Spain and Colombia studies, *C*. *matruchotti* was detected in higher proportions in periodontal health/gingivitis compared to periodontitis samples [59].

In the Placebo group, shifts in relative abundance of most taxa were modest. *C*. *gingivalis* and *Schaalia* spp. increased post-therapy, whereas *Bergeyella sp*. HMT-322 was associated with a greater % of reduction in bleeding. This latest species has been significantly associated with periodontal health [60, 61]. Regarding *Capnocythophaga* species, findings were also strain-specific. *C*. *gingivalis* and *Capnocythophaga* sp. HMT-380 augmented in the biofilm samples of the Placebo and Probiotic groups, respectively. Contradictory data has shown that the prevalence of *C*. *gingivalis* in the subgingival biofilm was similar among healthy, gingivitis and periodontitis individuals [62], whereas others reported higher proportions of *Capnocytophaga* spp. in healthy or shallow pockets than in moderate/deep periodontitis sites [58, 63, 64]. In contrast, some studies indicated that *Capnocytophaga* spp. were more enriched in gingivitis than in periodontal health and periodontitis [21, 26, 60, 65]. Another interesting finding obtained in the multiple regression model was the high prediction of reduction in the oral taxon *Treponema* HMT231 to a greater reduction in BOMP in the Placebo group, in contrast to the findings of Al-Kamel et al. (2019) [65], but corroborating data from other reports that associated this species with gingivitis [1].

A common finding in both treatments was the significant correlation between the enrichment of certain species of *Actinomyces* and less reduction of sites with BOMP. *Actinomyces* spp. have been related to periodontal health status [58, 60, 61, 64, 66]. However, some species such as *Actinomyces naeslundii* may be detected in high abundance in health and gingivitis [21, 26, 60]. At the genus level, *Actinomyces* were among the most abundant taxa in both groups before and after treatments. Likewise, the genus *Prevotella* was predominant in these gingivitis patients, even after therapies. Although without significant differences, *P*. *oralis* increased in both groups; however, its increase was a predictor of a greater reduction in gingival bleeding in the Probiotic group. Also, the specific taxon *Prevotella* sp. HMT-472 increased significantly only in the Probiotic group. *Prevotella* was associated with clinical signs of gingivitis and with an increase in the levels of inflammatory mediators such as IL-1α, IL-1ß, IL-1Ra, and lactoferrin in GCF [67]. Other *Prevotella* spp., on the other hand, were found in higher abundance in subgingival/submucosal biofilm of healthy periodontal and peri-implant sites in comparison with periodontitis and peri-implantitis sites [68]. In fact, Wirth et al. (2021) [69] report on the heterogeneity of *Prevotella* spp. across groups with different periodontal status. *Prevotella* spp. are generally considered commensal bacteria due to their extensive presence in the healthy human body and their relatively rare involvement in disease [70]. Host-related factors as well as the ecological relationship of these taxa with other members of the microbiome influence the diversity and prevalence of *Prevotella* species and strains in the human microbiome, as well as the extent of their contribution to human metabolism [71]. This genus is one with high genetic diversity within and between species, thus specific *Prevotella* species may exhibit vastly different properties [70]. This kind of species heterogeneity also brings to attention the duality attributed to several genera and the constant need for in-depth metagenomic characterization of the oral microbiota in health and disease to shed light on disease‐modulating properties.

In this study, both groups presented clinical improvements as well as microbiological changes, with a decrease of some pathogenic and an increase of some health-compatible species. However, these effects were more evident in the Probiotic group. From the perspective of the ecological plaque hypothesis [72], changes in the environment may favor or hinder the colonization of the dental biofilm by different species. It is important to emphasize that the probiotic’s effects are not restricted to the microbiological aspect since their immunomodulatory effects may have a great impact on the host [38, 73]. The ability of probiotics to improve the immune response by reducing proinflammatory markers, increasing anti-inflammatory markers, enhancing the epithelial barrier function, and both innate and adaptive responses has been demonstrated [9, 10, 21, 22, 74]. Here, immunoinflammatory effects were not presented, but they have been previously shown in the first published part of this study [23].

Some inconsistencies in the microbiological results between the present study and others in the literature using Next-Generation Sequencing technologies (NGS) do exist [75, 76]. Distinct sequencing methods, inaccuracy in obtaining the sequences, or inaccurate in periodontal diagnostic criteria may contribute to these differences. The use of ASVs instead of operational taxonomic unit (OTUs), as performed in the present study, could reduce bias, providing a higher resolution in the assignment of taxonomic categories and avoiding erroneous classification in taxonomic groups [59]. Furthermore, it is important to understand the polymicrobial perspective, that is, the healthy or pathogenic state are due to an integrated network and collective activity of microorganisms, and is not restricted only to key pathogens [27, 28]. Since NGS technologies are relatively recent, the benefits of periodontal and/or probiotic therapy in the complex oral microbiome have not yet been elucidated, and it is still unclear whether the microbial community after periodontal therapy resembles that found in health or remains with the characteristics of a disease-associated microbiome [26]. Limitations in the current study must also be considered when interpreting these findings. Besides the lack of immunological evaluations, our data is restricted to a short period of post-therapy assessment. Longer follow-ups would provide more information regarding the maintenance of the clinical and microbiological improvements observed with this probiotic. Nevertheless, the high-throughput sequencing microbial data here produced provide insights on some explanations for the significant reductions in gingival inflammation in both therapeutic groups, and for the additional improvements obtained in the Probiotic group.

## Conclusion

The adjuvant use of *B*. *lactis* HN019 alongside conventional therapy enhanced the reduction in BOMP and promoted greater changes in the microbiological profile of patients with generalized gingivitis.

## Supporting information

S1 Checklist CONSORT 2010 checklist of information to include when reporting a randomised trial. (PDF)

S2 Checklist SPIRIT-outcomes 2022 checklist. (PDF)

S1 Appendix Research project submitted for approval by the ethics committee. Original version in Portuguese.
(PDF)

S2 Appendix Research project submitted for approval by the ethics committee. English version.
(PDF)

S3 Appendix Informed consent form. Original version in Portuguese.
(PDF)

S4 Appendix Informed consent form. English version.
(PDF)

S1 Text Bacterial DNA extraction, 16S rRNA gene sequencing and bioinformatics analysis. (DOCX)

S1 Table Relative abundance of bacterial genera in the gingival biofilm of gingivitis patients pre- and post-therapies. * represents significant differences within the placebo group at different time-points (Wilcoxon test. p<0.05). ** represents significant differences within the probiotic group at different time-points (Wilcoxon test. p<0.05). *** represents significant differences between groups post-therapy (Mann-Whitney. p<0.05). Post-therapy: 8 weeks after treatment.
(DOCX)

S2 Table Correlation between oral genera and % of reduction in BOMP at both therapeutic groups. *p< 0.05; **p<0.01. Numbers refer to the Spearman’s correlation coefficient (rho). T8: 8 weeks after initial therapy.
(DOCX)

S3 Table Relative abundance of bacterial taxa at species level. Mean abundance of oral species detected at ≥ 0.1% for all samples at baseline. * Refers to taxa that differed between therapeutic groups at baseline or at 8 weeks post-therapy (Mann-Whitney test. p < 0.05). Green shading represents significant differences between baseline and post-therapy within each group (Wilcoxon test. p < 0.05).
(DOCX)

S4 Table Multiple linear regression for prediction of reduction in BOMP based on changes in relative abundance of oral species/phylotypes post-therapy in the Placebo group. Dependent variable: reduction on bleeding on marginal probing (BOMP). Changes in the abundance of 441 oral species were entered as predictor variables in the analysis using the stepwise method. The model that met all assumptions was obtained at step 6. Decrease in abundance of *Treponema* sp. HMT-231 after treatment was a good predictor of high reduction in gingival bleeding. Increase in abundances of *Bergeyella sp*. HMT-322 and *Schaalia sp*. HMT-178 after treatment were good predictors of high reduction in gingival bleeding.
(DOCX)

S5 Table Multiple linear regression for prediction of reduction in BOMP based on changes in relative abundance of oral species/phylotypes post-therapy in the Probiotic group. Dependent variable: reduction on bleeding on marginal probing (BOMP). Changes in the abundance of 441 oral species were entered as predictor variables in the analysis using the stepwise method. The model that met all assumptions was obtained at step 6. Decrease in abundances of *Actinomyces* sp. HMT-175 and *Leptotrichia* HMT-498 after therapy were good predictors of high reduction in gingival bleeding.
(DOCX)
