# Supplementary material for: Microbiological profile of patients with generalized gingivitis undergoing periodontal therapy and administration of Bifidobacterium animalis subsp. lactis HN019: A randomized clinical trial
Source: PLoS One. 2024 Nov 11;19(11):e0310529. doi: 10.1371/journal.pone.0310529 (PMC11554181; doi:10.1371/journal.pone.0310529)
Supplement: S1 Appendix — Original version in Portuguese. (PDF) [file pone.0310529.s003.pdf]

S1 Appendix. Research project submitted for approval by the ethics committee. Original version in Portuguese.

Projeto de Pesquisa:
"Efeitos da terapia probiótica na gengivite: estudo do perfil clínico, microbiológico e imunológico da resposta do hospedeiro."

Informações Preliminares

Responsável Principal

CPF/Documento: 831.791.271-04	Nome: Flávia Aparecida Chaves Furlaneto Messora
Telefone: 16982020181	E-mail: flafurlaneto@hotmail.com

Instituição Proponente

CNPJ: 63.025.530/0086-01	Nome da Instituição: Universidade de Sao Paulo

Essa submissão de emenda é exclusiva do seu Centro Coordenador?

A emenda é exclusiva de seu Centro Coordenador, então as alterações realizadas em seu projeto, em virtude da emenda, NÃO serão replicadas nos Centros Participantes vinculados e nos Comitês de Ética das Instituições Coparticipantes, quando da sua aprovação.

É um estudo internacional? Não

■ Assistentes

CPF/Documento	Nome
015.231.336-27	Renata Cardoso

■ Equipe de Pesquisa

CPF/Documento	Nome
042.597.906-76	Michel Reis Messora
034.019.661-03	PEDRO HENRIQUE FELIX SILVA
054.381.405-02	RAFAEL MORAES CHAVES SANTOS

Área de Estudo

Grandes Áreas do Conhecimento (CNPq)

- Grande Área 4. Ciências da Saúde

Propósito Principal do Estudo (OMS)

- Clínico

Título Público da Pesquisa: "Efeitos da terapia probiótica na gengivite: estudo do perfil clínico, microbiológico e imunológico da resposta do hospedeiro."

Contato Público

CPF/Documento	Nome	Telefone	E-mail
831.791.271-04	Flávia Aparecida Chaves Furlaneto Messora	16982020181	flafurlaneto@hotmail.com

Contato Científico: Flávia Aparecida Chaves Furlaneto Messora

Desenho de Estudo / Apoio Financeiro

Desenho do Estudo: Intervenção/Experimental

Condições de saúde ou problemas

Condição de saúde ou Problema
Gengivite

Descritores Gerais para as Condições de Saúde

CID1-10:Classificação Internacional de Doenças

Código CID	Descrição CID
K05	Gengivite e doencas periodontais

DeCS:Descritores em Ciência da Saúde

Código DECS	Descrição DECS
C07.465.714.258.480	Gengivite

Descritores Específicos para as Condições de Saúde

CID1-10:Classificação Internacional de Doenças

Código CID	Descrição CID
K05	Gengivite e doencas periodontais

DeCS:Descritores em Ciência da Saúde

Código DECS	Descrição DECS
C07.465.714.258.480	Gengivite

Tipo de Intervenção: Experimental

Natureza da Intervenção

- Procedimento/operatória/cirurgia

Descritores da Intervenção

Lista de CID

Código CID	Descrição CID
K05	Gengivite e doencas periodontais

Lista de DECS

Código DECS	Descrição DECS
C07.465.714.258.480	Gengivite

Fase

- Fase 1

Desenho:

Estudo clínico randomizado, duplo-cego.

Apoio Financeiro

CNPJ	Nome	E-mail	Telefone	Tipo
				Financiamento Próprio

Palavra Chave

Palavra-chave
Gengivite
Probiótico
Controle químico

Resumo:

Considerando as limitações da higiene oral da população em geral e também a participação de vários mecanismos referentes ao sistema imune inato e adaptativo do hospedeiro na patogênese das doenças periodontais, o uso de probióticos como uma nova terapia adjuvante para redução de placa e gengivite tem despertado o interesse da comunidade científica odontológica, já que os mesmos podem modular a resposta imunoinflamatória do hospedeiro e modificar o ambiente bacteriano. O propósito deste estudo clínico randomizado, duplo-cego e placebo-controlado será avaliar os efeitos da administração oral da cepa probiótica Bifidobacterium animalis subsp. lactis HN019 (B. lactis HN019) em pacientes portadores de gengivite generalizada. Sessenta indivíduos com doença periodontal (gengivite generalizada) receberão o tratamento periodontal padrão-ouro para gengivite (profilaxia e/ou raspagem supragengival) 1 semana (-1s) antes do início do estudo. Os pacientes serão orientados, logo após a primeira consulta (baseline), a consumirem pastilhas contendo B. lactis HN019 (grupo teste) ou placebo (grupo controle) uma vez ao dia, durante 56 dias. Parâmetros clínicos periodontais, imunológicos e microbiológicos serão avaliados no baseline (período pré-intervenção) e após 28 e 56 dias do início da administração de probiótico ou placebo. Os parâmetros clínicos avaliados serão: índice de placa, índice gengival, índice de sangramento gengival, profundidade clínica de sondagem, nível clínico de inserção, sangramento à sondagem. Os parâmetros imunológicos serão analisados por meio de imunoensaios enzimáticos (LuminexTM xMAP®) para verificação dos níveis das citocinas IL-1, IL-1, IL-8, RANTES, MCP-1 e MIP-1 presentes no fluido crevicular gengival. Será realizada coleta das placas bacterianas supra e subgengival para análise da composição microbiológica do biofilme por meio de checkerboard DNA-DNA hybridization e para detecção e quantificação de B. lactis HN019 no biofilme, por meio de Reação em Cadeia da Polimerase (qPCR). Os dados obtidos serão estatisticamente analisados ($p<0.05$).Palavras-chave: Gengivite; Probiótico; Controle químico.

Introdução:

1. QUALIFICAÇÃO DO PRINCIPAL PROBLEMA A SER ABORDADO1.1. Doenças periodontais (DPs): perspectivas de tratamento As Doenças Periodontais (DPs) compreendem um grupo de doenças inflamatórias, incluindo gengivite e periodontite, que afetam os tecidos de proteção e suporte dos dentes e podem levar à perda dentária, atingindo grande parte da população. O levantamento epidemiológico SB Brasil 2010 demonstrou que o sangramento gengival aumenta gradativamente com o aumento da idade, afetando metade dos indivíduos com faixa etária entre 35 e 44 anos de idade. Em um recente levantamento epidemiológico realizado nos Estados Unidos, foi demonstrado que um em cada dois americanos com 30 anos de idade ou mais possui periodontite (EKE et al., 2012).O biofilme bacteriano é o fator etiológico primário para o início da inflamação gengival e subsequente destruição dos tecidos periodontais (HAFFAJEE & SOCRANSKY, 1994). A resposta imunoinflamatória do hospedeiro às agressões bacterianas é responsável pela patogênese e progressão da doença de gengivite para periodontite (SALVI & LANG, 2005). Fatores de risco adquiridos e ambientais (ex.: diabetes mellitus, fumo e estresse), bem como algumas características geneticamente transmitidas (ex.: polimorfismos gênicos para a Interleucina [IL]-1) podem acentuar a resposta inflamatória decorrente da agressão bacteriana e, eventualmente, a suscetibilidade às DPs (SALVI & LANG, 2005). As DPs tem sido associadas a aproximadamente 57 desordens sistêmicas, como diabetes mellitus, doenças coronarianas, efeitos adversos na gravidez, câncer de mama, dentre outros (AMÓDIO et al., 2014; MONSARRAT et al., 2016). Esses efeitos sistêmicos levaram ao entendimento de que encontrar um tratamento eficaz para a doença inflamatória gengival crônica é vital não só para a prevenção da perda de dentes, mas também como parte dos cuidados de saúde global do paciente (SAMUELS et al., 2012; MONSARRAT et al., 2016).A remoção mecânica da placa bacteriana é o método mais eficaz para controle da gengivite, que em um estudo sobre o desenvolvimento da gengivite a inflamação do tecido gengival era compatível com o acúmulo de biofilme. (LOE et al., 1965). Entretanto, a população em geral não realiza um controle de placa bacteriano adequado (PETERSEN & OGAWA, 2005). As estratégias baseadas nas recomendações dos profissionais, ou seja, em métodos de demonstração e de diálogo com os pacientes sobre como melhorar sua higiene oral, não levam aos resultados esperados (KOTTKE et al., 1988; BIEN et al., 1993). Tem sido demonstrado que aproximadamente 30 a 60% das informações fornecidas pelos profissionais são esquecidas em um intervalo de uma hora e que 50% das recomendações de saúde não são seguidas (DIMATTEO et al., 2012). Com base no consenso do 11th European Workshop on Periodontology on effective prevention of periodontal and peri-implant diseases, sabe-se que o paciente precisa adquirir atitudes positivas para uma mudança comportamental, e que atingir uma verdadeira mudança de comportamento em relação a um controle de placa adequado é uma das responsabilidades mais desafiadoras dos profissionais, além de poder levar um longo tempo (TONETTI et al., 2015).O fato de que a população em geral não se submete a adequada higiene oral associado ao atual conhecimento sobre a influência das DPs na saúde sistêmica do indivíduo tem incitado o desenvolvimento de agentes para controle da formação do biofilme e/ou para modulação da resposta imunoinflamatória do hospedeiro à placa (VAN DER OUDERAA, 1991; PULIKKOTIL & NATH, 2015). Tem sido demonstrado que o uso dos enxaguatórios bucais antimicrobianos com clorexidina e óleos essenciais são os mais adequados para promover controle de placa e de gengivite em longo prazo (CHARLES et al., 2004). Contudo, os efeitos adversos dos bochechos com clorexidina, como alteração do paladar, aumento da formação de cálculo supragengival, lesões em tecidos moles em pacientes jovens, respostas alérgicas e manchas em dentes e tecidos moles, levantam um questionamento a respeito da sua eficácia para uso em longo prazo (FLOTRA et al., 1971; QUIRYNEN et al., 2001). Atualmente, existe uma grande busca por um agente que apresente as mesmas propriedades da clorexidina na inibição do desenvolvimento da gengivite mas sem apresentar seus efeitos adversos, podendo ser usado por um longo período de tempo. A possibilidade de modular a resposta do hospedeiro nas DPs, interferindo na expressão de citocinas pró-inflamatórias, foi demonstrada em um estudo realizado por Assuma et al. (1998). Neste estudo, a periodontite foi induzida pela colocação de ligaduras infectadas com Porphyromonas gingivalis em primatas. A injeção local de antagonistas de algumas interleucinas pró-inflamatórias reduziu em 80% o recrutamento de células inflamatórias, em 67% a formação de osteoclastos e em 60% a perda óssea quando comparados com dentes controles que não receberam a injeção local dos antagonistas (ASSUMA et al., 1998). Nesse contexto, considerando as limitações da higiene oral e também a participação de vários mecanismos referentes ao sistema imune inato e adaptativo do hospedeiro na patogênese das DPs, tais como liberação de citocinas pró-inflamatórias e anti-inflamatórias (COCHRAN, 2008; SCHERES et al., 2010; DUTZAN et al., 2012), produção de beta-defensinas (BD) (LU et al., 2005), ativação de receptores do tipo “Toll” (TLR) (HANS & HANS, 2011) e infiltração de células “natural killer” (STELIN et al., 2009), o uso de probióticos como uma nova terapia adjuvante para redução de placa e gengivite tem despertado o interesse da comunidade científica odontológica, já que os mesmos podem modular a resposta imunoinflamatória do hospedeiro e modificar o ambiente bacteriano. Recentemente, uma revisão sistemática e meta-análise demonstrou que a evidência científica atual sustenta o uso de probióticos na abordagem terapêutica da gengivite e da periodontite (GRUNER et al., 2016).1.2. Probióticos: nova abordagem para o tratamento das DPsA terapia probiótica pode proporcionar vantagens que não são observadas quando antibióticos e/ou antissépticos são utilizados para controle químico de placa (KARUPPAIAH et al., 2013), pois não promove resistência bacteriana e pode interferir naturalmente na resposta imunoinflamatória local e sistêmica do hospedeiro. Um recente estudo de “prova de conceito” demonstrou que uma mistura de espécies de Streptococcus aplicada em dentes de cães com periodontite, como uma terapia adjuvante à raspagem e alisamento radicular (RAR), atrasou a recolonização de patógenos periodontais e reduziu a inflamação (TEUGHELS et al., 2007). Os probióticos são definidos como microrganismos vivos, principalmente bactérias, seguros para o consumo e capazes de produzirem efeitos benéficos para a saúde do hospedeiro quando ingeridos em quantidades suficientes (FAO/WHO, 2002). O consumo de probióticos pode potencializar o sistema imune do hospedeiro, bem como agir na prevenção/tratamento de certas doenças e/ou incômodos em humanos, tais como diarreia devido à infecção com Helicobacter pylori, má digestão de lactose e síndrome do intestino irritável. Além disso, atuam na prevenção do câncer de cólon e de bexiga, no controle dos níveis de colesterol e da hipertensão arterial, na proteção contra infecções do trato urinário e respiratório e na supressão de alergias (OUWEHAND et al., 2003; AMROUCHE, 2005; COMMANE et

al., 2005). Os probióticos são geralmente regulamentados como suplementos dietéticos e comercializados para melhorar ou manter a saúde (TSUBURA et al., 2009). Os principais microrganismos utilizados para fins probióticos são bactérias do gênero Lactobacillus e Bifidobacterium (TEUGHELS et al., 2008). O primeiro estudo utilizando probióticos e tendo como alvo a cavidade oral foi realizado há mais de 60 anos como tratamento para inflamações das mucosas (KRAGEN, 1954). Mas apenas recentemente a cavidade oral tem sido sugerida como um alvo relevante para as aplicações dos probióticos (MEURMAN, 2005). Os probióticos foram avaliados, principalmente, no controle da cárie dentária, podendo reduzir os níveis de Streptococcus mutans na saliva (NÄSE et al., 2001; AHOLA et al., 2002; CAGLAR et al., 2006; CAGLAR et al., 2008). Os mecanismos de ação dos probióticos na cavidade bucal parecem ser análogos àqueles descritos para o equilíbrio da microflora intestinal (HAUKIOJA, 2010). Estes mecanismos podem ser uma alternativa não apenas para o controle da cárie dentária, mas também para o tratamento da gengivite e periodontite. Os microrganismos utilizados para fins probióticos podem desencadear efeitos diretos sobre os patógenos periodontais, afetando seu crescimento, adesão e colonização (STAMATOVA & MEURMAN, 2009). Bactérias probióticas podem produzir diversos componentes que agem como agentes antimicrobianos, tais como ácido lático, peróxido de hidrogênio, bacteriocinas e substâncias inibitórias semelhantes às bacteriocinas (GILLOR et al., 2008; GORDON, 2009; OELSCHLAEGER, 2010). Sookkhee et al. (2001) isolaram bactérias produtoras de ácido lático da cavidade oral de voluntários saudáveis tailandeses e demonstraram que as mesmas desenvolviam atividade antimicrobiana contra Porphyromonas gingivalis e Streptococcus mutans. Van Hoogmoed et al. (2000) observaram que um biosurfactante produzido pelo Streptococcus mitis é capaz de diminuir a adesão de S. mutans e de vários periodontopatógenos. Outro mecanismo sugerido para explicar a ação dos probióticos no tratamento das DPs refere-se à modulação da resposta imunoinflamatória do hospedeiro (STAMATOVA & MEURMAN, 2009). Alguns estudos tem demonstrado que algumas espécies probióticas podem atenuar a expressão de IL-8 induzida por periodontopatógenos nas células epiteliais orais (COSSEAU et al., 2008; ZHANG et al., 2008; SLIEPEN et al., 2009) e reduzir os níveis de citocinas pró-inflamatórias (IL-8, IL- e Fator de Necrose Tumoral [TNF]-) no fluido crevicular gengival (TWETMAN et al., 2009). Shimauchi et al. (2008) verificaram que o consumo de probióticos diminuiu significativamente os níveis de lactoferrina salivar, uma proteína indicativa de inflamação periodontal, em indivíduos altamente susceptíveis à periodontite. Staab et al. (2009) demonstraram que a ingestão de probióticos pode reduzir a atividade de elastase de polimorfonucleares, bem como os níveis de mieloperoxidase e metaloproteinase-3 da matriz no fluido crevicular gengival de indivíduos com gengivite. Os estudos (SHIMAUCHI et al., 2008; STAAB et al., 2009; IERARDO et al., 2010; SLAWIK et al. 2011; INIESTA et al., 2012; HALLSTROM et al., 2013; LEE et al., 2015; NCE et al., 2015; TEKCE et al., 2015; NADKERNY et al., 2016; MORALES et al., 2016) que investigaram os efeitos dos probióticos nas DPs até hoje usaram principalmente microorganismos do gênero Lactobacillus. Contudo, outros probióticos em potencial merecem ser investigados. O agente Bifidobacterium animalis subsp. lactis HN019, originado de lácteos, é considerado um probiótico em potencial e tem a capacidade de resistir à ação da bile e a pH bem ácidos (PRASAD et al., 1998). Essa estirpe também é capaz de se aderir em quantidades elevadas em diferentes tipos de células do epitélio intestinal (GOPAL et al., 2001) e apresenta interessantes propriedades imunomoduladoras (GILL et al., 2000). Estudos desenvolvidos em humanos mostraram que B. lactis HN019 foi capaz de melhorar a resposta imune inata de indivíduos idosos e de meia-idade (GILL et al., 2001), proporcionou um aumento na atividade citotóxica das células “natural killer” e na atividade fagocítica dos monócitos periféricos, sendo esta atividade persistente até seis semanas após a interrupção do consumo do probiótico (ZHOU & GILL, 2005), diminuiu a deficiência em ferro em crianças pré-escolares, proporcionando ganho de peso (SAZAWAL et al., 2010) e promoveu a proteção dos enterócitos frente a uma infecção aguda (LIU et al., 2010). Um estudo realizado em animais demonstrou que 80% dos ratos tratados com B. lactis HN019 diariamente durante uma semana permaneceram vivos por três semanas após serem infectados com Salmonella typhimurium. Nos animais que não consumiram o probiótico, a taxa de mortalidade foi de 93% (SHU et al., 2000). Em um estudo realizado por nosso grupo, constatou-se que a ingestão do probiótico B. lactis HN019 por camundongos promove uma inversão na razão de células B-1/B-2 (BOGSAN et al., 2014). Células B compreendem dois subtipos distintos: linfócitos B convencionais (ou B-2) e linfócitos B-1. A presença aumentada de células B-1 em indivíduos com periodontite (5 a 6 vezes mais do que em indivíduos sem periodontite) pode explicar a maior destruição dos tecidos periodontais observada nesses pacientes (BERGLUNDH et al., 2002). Há relatos de que a modulação da reabsorção óssea in vivo e a diferenciação das células semelhantes a osteoclastos pode ser promovida pela interação de células B-1 (PUGLIESE et al., 2012), uma vez que células multinucleadas dependem de células B-1 para sua formação (BOGSAN et al., 2005). Pelo conhecimento que temos até hoje, nosso grupo realizou recentemente o primeiro estudo com a administração de B. lactis em DPs. Os efeitos da administração local do B. lactis HN019 foram analisados em ratos com periodontite induzida por ligadura (OLIVEIRA et al., 2016). Observou-se que os animais tratados apresentaram maiores proporções de espécies semelhantes a Actinomyces e Streptococcus e menores proporções de espécies semelhantes a Eikenella corrodens e Prevotella intermedia do que os animais não tratados. Além disso, houve maiores expressões de OPG e de beta-defensinas e menores expressões de IL-1 e RANKL nos animais que receberam B. lactis HN019 do que nos animais não tratados. Todos esses resultados obtidos com o uso do B. lactis HN019 sugerem que esta estirpe probiótica pode ser bastante útil no tratamento das DPs. Ainda há poucos estudos clínicos avaliando os efeitos de probióticos na gengivite. Esses estudos foram realizados em modelos de gengivite experimental com a suspensão da higiene oral em todos os dentes dos indivíduos (STAAB et al., 2009; LEE et al., 2015) ou em apenas alguns dentes (HALLSTROM et al., 2013; SLAWIK et al., 2011) e também com a administração de probióticos em pacientes já com a gengivite instalada (TWETMAN et al., 2009; KRASSE et al., 2006; IERARDO et al., 2010; INIESTA et al., 2012). Estudos clínicos comprovam que a remoção mecânica da placa bacteriana leva a completa resolução da inflamação gengival, ou seja, a uma gengiva clinicamente saudável, sem efeitos deletérios para os pacientes, em poucos dias após início dos procedimentos de higiene oral adequados (LOE et al., 1965; PANCER et al., 2016). Dentre os estudos clínicos que avaliaram os efeitos de probióticos na gengivite, não há nenhum com a utilização da cepa B. lactis HN019. De um modo geral, os estudos clínicos demonstraram que o uso de probióticos pode promover significativa redução de periodontopatógenos (INIESTA et al., 2012), melhorar os parâmetros clínicos gengivais (KRASSE et al., 2006; HARINI & ANEGUNDI, 2010; IERARDO et al., 2010; LEE et al., 2015, NADKERNY et al., 2015), reduzir marcadores inflamatórios do fluido crevicular gengival (FCG) (STAAB et al., 2009; TWETMAN et al., 2009; LEE et al., 2015) ou da saliva (SHIMAUCHI et al., 2008; IERARDO et al., 2010) e inibir o desenvolvimento da gengivite (STAAB et al., 2009; SLAWIK et al., 2011; KARUPPAIAH et al., 2013; LEE et al., 2015). No estudo de Hallstrom et al. (2013), os autores não conseguiram demonstrar um efeito protetor da administração de Lactobacillus no padrão inflamatório ou na composição microbiológica da placa supragengival de pacientes com gengivite experimental. No entanto, como os próprios autores realçam, são necessários mais estudos clínicos para elucidar o papel dos probióticos no tratamento da gengivite, periodontite e, ainda, da mucosite periimplantar. É importante ressaltar também que ainda há uma grande lacuna na literatura sobre os reais mecanismos de ação dos probióticos para o tratamento e prevenção das DPs, principalmente no que se refere à atuação dos mesmos na modulação da resposta imunoinflamatória periodontal do hospedeiro. Como mecanismos imunoinflamatórios similares, determinantes no processo de saúde-doença do hospedeiro, ocorrem nos tecidos periodontais e na mucosa intestinal, acredita-se que a ação imunoinflamatória dos probióticos na cavidade bucal seja análoga àquela descrita na mucosa intestinal. Estudos recentes demonstraram que probióticos podem: potencializar a imunidade do hospedeiro por meio de um aumento na expressão de beta-defensina (BD) na mucosa intestinal (DENG et al., 2013) da mesma forma Oliveira et al; (2016) obteve resultados semelhantes no epitelio oral, reforçar a barreira epitelial intestinal aumentando a expressão de TLR (CASTILLO et al., 2011), reduzir a inflamação intestinal interferindo na expressão de células positivas para o Grupamento de Diferenciação (CD)-4, CD-8, CD-57 e foxhead box P3 (Foxp3) ou na via do Fator Nuclear Kappa-beta (JEON et al., 2012; NISHITANI et al., 2009), diminuir a produção de citocinas pró-inflamatórias (IL-1, IL-4, IL-8, IL-17, TNF-, Interferon [INF]-, Proteína Quimiotática de Monócitos [MCP-1] e Fator Estimulador de Colônia de Macrófagos [M-CSF]) (PHILIPPE et al., 2011; ZHU et al., 2012; RODRIGUES et al., 2012; OKAMOTO et al., 2012; MARIMAN et al., 2012; BADIA et al., 2012) e aumentar a produção de citocinas anti-inflamatórias (IL-10 e Fator de Crescimento Transformador [TGF]-) (FINAMORE et al., 2012; RODRIGUES et al., 2012; ARRIBAS et al., 2012). Dessa forma, novos estudos são fundamentais para explorar esses mecanismos de ação dos probióticos nos tecidos periodontais. A

terapia probiótica ainda se encontra em um “estágio de infância” para os cuidados da saúde periodontal, mas abre, sem dúvidas, uma porta para o novo paradigma de tratamento das DPs em um modo nanomolecular (CHATTERJEE et al., 2011). Muitos estudos clínicos prévios que avaliaram o uso de probióticos nas DPs produziram resultados inconsistentes, o que pode ser atribuído a diferenças nas cepas utilizadas e ao delineamento dos estudos, que pode permitir viéses (LEE et al., 2015). Portanto, novos estudos são fundamentais para melhor elucidar o papel das bactérias benéficas na cavidade bucal, identificar novas espécies de bactérias, estabelecer tempos e novos veículos de administração e determinar a real significância clínica desta terapia (TEUGHELS et al., 2008; STAMATOVA & MEURMAN, 2009; TONETTI & CHAPPLE, 2011).

Hipótese:

Espera-se que o uso de probióticos (grupo teste) potencialize os efeitos da terapia periodontal padrão-ouro, levando a melhores resultados em relação aos parâmetros clínicos periodontais, microbiológicos e imunológicos, quando comparados ao grupo controle.

Objetivo Primário:

Avaliar os efeitos da administração oral da cepa probiótica B. lactis HN019 como adjuvante ao tratamento da gengivite em humanos.

Objetivo Secundário:

Em pacientes portadores de gengivite generalizada, com administração oral de probióticos ou placebo, avaliar:
Parâmetros clínicos: índice de placa, índice gengival, índice de sangramento gengival, profundidade clínica de sondagem, nível clínico de inserção, sangramento à sondagem;
Níveis das citocinas IL-1, IL-1, IL-8, RANTES, MCP-1 e MIP-1 presentes no fluido crevicular gengival, por meio de imunoensaios enzimáticos (LuminexTM xMAP®);
Microbiota do biofilme, por meio de checkerboard DNA-DNA hybridization;
Detecção e quantificação de Bifidobacterium animalis subsp. lactis HN019 no biofilme, por meio de Reação em Cadeia da Polimerase (qPCR).

Metodologia Proposta:

Consentimento para a pesquisa (Termo de Consentimento Livre e Esclarecido), submissão do projeto junto ao CEP; Cálculo amostral(total= 60, n= 30); Seleção dos pacientes (Clínica de Pós-graduação FORP-USP);Todos os pacientes serão submetidos a um exame radiográfico periapical completo da boca toda. Os voluntários serão agrupados em um programa de higiene oral (PHO) de acordo com suas necessidades específicas. Neste programa, os pacientes receberão instruções para um efetivo autocontrole de placa bacteriana, incluindo informações sobre a técnica de Bass (BASS, 1954) e limpeza interproximal com fio dental. Eles serão também motivados a escovar o dorso da língua uma vez ao dia e receberão uma escova dental e um mesmo dentifrício que deverá ser utilizado durante todo o período experimental (Colgate Total®, Anakol Ind. Com. Ltda - Kolynos do Brasil – Colgate Palmolive Co., São Bernardo do Campo, SP, Brasil). Antes de iniciar o estudo, os indivíduos selecionados serão identificados por um código numérico. De acordo com uma tabela numérica aleatória gerada por um programa computadorizado, o coordenador do estudo alocará cada paciente em um dos seguintes grupos experimentais: controle (placebo) ou teste (terapia probiótica). Os voluntários não saberão a qual grupo experimental pertencem. Uma semanas antes das coletas iniciais (-1s) todos os pacientes incluidos na pesquisa receberão profilaxia e/ou raspagem supragengival, conforme a necessidade. No dia 0, todos os indivíduos receberão pastilhas apresentando a mesma composição, textura, formato, sabor, com exceção da presença do probiótico. No grupo teste, as pastilhas de 10 mg conterão 109 unidades formadoras de colônias (UFCs) de Bifidobacterium animalis subsp. lactis HN019. Os indivíduos serão orientados, logo após a primeira consulta (baseline), a consumirem a pastilha uma vez ao dia, durante 8 semanas. Eles serão orientados a consumir a pastilha sempre após a última escovação, antes de dormir, deixando a pastilha dissolver na boca. Durante os 56 dias em que consumirão as pastilhas, os voluntários serão orientados também a conservarem as pastilhas em geladeira, a não consumirem nenhum outro produto probiótico e a não fazerem uso de nenhum produto para controle químico de placa bacteriana, com exceção do dentifrício. Após 28 dias do início da ingestão diária de Bifidobacterium animalis subsp. lactis HN019, os pacientes serão orientados a retonarem para coleta de novos dados clínicos, sendo que deverão ficar 8 horas anteriores ao exame sem realização de nenhuma medida de higiene, com exceção das pastilhas, (AYALA., et al.; 2016). Os participantes serão instruídos a manter sua rotina de higiene oral durante o decorrer do estudo. Os parâmetros clínicos periodontais, imunológicos e microbiológicos serão avaliados em três períodos: no baseline (período pré-intervenção), após 28 dias do início da administração de probiótico ou placebo e após 56 dias do início da administração de probiótico ou placebo. Os exames clínicos periodontais serão realizados por um único examinador devidamente treinado e calibrado, o qual desconhecerá os grupos experimentais aos quais pertence cada voluntário. Os examinadores das avaliações imunológicas e microbiológicas também serão cegos quanto aos grupos experimentais das amostras analisadas. As pastilhas probióticas e não probióticas (placebo) serão preparadas por uma farmácia de manipulação e ambas apresentarão o mesmo formato e serão acondicionadas em frascos idênticos. As pastilhas serão enviadas ao coordenador do estudo, o qual marcará o código numérico de cada paciente em um conjunto de 56 pastilhas (quantidade a ser consumida por cada indivíduo durante 8 semanas), de acordo com o grupo experimental a que pertence. As embalagens codificadas serão enviadas ao examinador clínico do estudo, o qual distribuirá aos pacientes e, em nenhum momento, terá informações sobre o conteúdo das mesmas. (Ver o restante no arquivo do projeto).

Critério de Inclusão:

Critérios de inclusão serão: (1) indivíduos sistemicamente saudáveis, (2) presença de inflamação gengival em mais de 30% dos sítios (IG=1), (3) presença de 28 20 dentes permanentes totalmente erupcionados, excluindo-se terceiros molares e dentes indicados para exodontia, (4) disposição em aderir ao protocolo do estudo.

Critério de Exclusão:

Critérios de exclusão para o presente estudo: (1) grávidas ou lactantes, (2) envolvimentos sistêmicos que possam interferir nos resultados do estudo (ex: diabetes mellitus, desordens imunológicas), (3) ingestão de antimicrobianos e/ou anti-inflamatórios nos últimos 6 meses e de probióticos ou antissépticos orais no último mês, (4) uso de medicação com implicações gengivais, (5) histórico ou presença de periodontite, (6) presença de doença gengival não induzida por placa, (7) alergias conhecidas aos materiais experimentais, (8) presença de aparelho ortodôntico, (9) envolvimentos protéticos extensos, (10) tabagismo, (11) pacientes legalmente incapazes.

Riscos:

Os riscos presentes são referentes ao desconforto que o paciente terá ao passar pelo tratamento periodontal padrão-ouro para gengivite (profilaxia e/ou raspagem supragengival). O uso das pastilhas contendo placebo ou probióticos não trará riscos à saúde do paciente.

Benefícios:

Equilíbrio da flora intestinal

Metodologia de Análise de Dados:

Será verificada a normalidade e homocedasticidade dos dados obtidos. As comparações inter e intra-grupos nos diferentes intervalos de tempo serão realizadas por meio de testes paramétricos ou não-paramétricos adequados. Para todas as análises estatísticas será utilizado um nível de significância de 5%. Todos os cálculos serão realizados pelo software SPSS (SPSS, Chicago IL, EUA).

Desfecho Primário:

Índice de Sangramento Gengival

Desfecho Secundário:

Índice de Placa de TureskyÍndice Gengival (LOE & SILNESS, 1963) Profundidade clínica de sondagem (mm)Nível clínico de inserção (mm)Sangramento à sondagem, avaliado dicotomicamente (AINAMO & BAY, 1975)Composição microbiológica do biofilme por meio de checkerboard DNA-DNA hybridizationQuantificação absoluta de Bifidobacterium animalis subsp. lactis HN019 no biofilme por qPCRQuantificação das citocinas (pg/l) IL-1, IL-1, IL-8, RANTES, MCP-1, MIP-1

Tamanho da Amostra no Brasil: 60

Países de Recrutamento

País de Origem do Estudo	País	Nº de participantes da pesquisa
Sim	BRASIL	60

Outras Informações

Haverá uso de fontes secundárias de dados (prontuários, dados demográficos, etc)?

Não

Informe o número de indivíduos abordados pessoalmente, recrutados, ou que sofrerão algum tipo de intervenção neste centro de pesquisa:

60

Grupos em que serão divididos os participantes da pesquisa neste centro

ID Grupo	Nº de Indivíduos	Intervenções a serem realizadas
Controle (terapia periodontal padrão-ouro)	30	Profilaxia e/ou raspagem supragengival, Consumo de pastilhas contendo placebo e Tratamento Periodontal adicional (se necessário) ao final do experimento.
Teste(terapia periodontal padrão-ouro eprobiótico)	30	Profilaxia e/ou raspagem supragengival, Consumo de pastilhas probióticas e Tratamento Periodontal adicional (se necessário) ao final do experimento.

O Estudo é Multicêntrico no Brasil?

Não

Propõe dispensa do TCLE?

Não

Haverá retenção de amostras para armazenamento em banco?

Não

Cronograma de Execução

Identificação da Etapa	Início (DD/MM/AAAA)	Término (DD/MM/AAAA)
Preparo de resumos para apresentação dos resultados em congressos nacionais e internacionais	01/05/2019	30/08/2019
Análise imunológica	01/08/2018	01/01/2019
Monitoramentos clínico, imunológico e microbiológico	01/08/2018	01/01/2019
Relatório final das atividades desenvolvidas	01/05/2019	30/08/2019
Análise estatística dos dados obtidos	01/02/2019	30/04/2019
Coleta de dados	01/03/2018	02/07/2018
Monitoramentos clínico, imunológico e microbiológico	01/05/2018	02/07/2018
Triagem dos pacientes-Aquisição parcial de materiais de consumo	01/08/2017	02/07/2018
Preparo de resumos para apresentação dos resultados em congressos nacionais e internacionais	28/02/2019	31/05/2019
Preparo dos manuscritos para publicação em periódicos de seletiva política editorial classificados com A1 no sistema Qualis da CAPES	01/05/2019	30/08/2019

Relatório parcial de atividades - 1	02/07/2018	31/07/2018
Análises microbiológica	01/08/2018	01/01/2019

Orçamento Financeiro

Identificação de Orçamento	Tipo	Valor em Reais (R$)
Curetas de Gracey	Custeio	R$ 1.500,00
Dentifrício (Colgate)	Custeio	R$ 500,00
Material de Consumo (Papel, xerox,impressões, clínicos, laboratoriais etc)	Custeio	R$ 5.000,00
PCR	Custeio	R$ 3.500,00
Sondas Clínicas Periodontais	Custeio	R$ 500,00
Escovas dentais (Colgate)	Custeio	R$ 800,00
Checkboard	Custeio	R$ 30.000,00
Luminex	Custeio	R$ 30.000,00
Total em R$		R$ 71.800,00

Bibliografia:

5. REFERÊNCIAS BIBLIOGRÁFICAS 1. Ahola AJ, Yli-Knuuttila H, Suomalainen T, Poussa T, et al. Short-term consumption of probiotic-containing cheese and its effect on dental caries risk factor. Arch Oral Biol., 47:799-804, 2002. 2. Ainamo J & Bay I. Periodontal indexes for and in practice. Tandlaegebladet, 80:149-52, 1975. 3. Amódio J, Palioto DB, Carrara HH, Tiezzi DG, et al. Oral health after breast cancer treatment in postmenopausal women. Clinics (Sao Paulo)., 69(10):706-8, 2014. 4. Amrouche T. Contribution à l étude du pouvoir immunomodulateur de bifidobactéries: analyse in vitro et étude ex vivo des mécanisms moléculaires impliques / Tahar Amrouche. Québec: Université Laval, 2005. 175p. 5. Arribas B, Garrido-Mesa N, Perán L, Camuesco D, et al. The immunomodulatory properties of viable Lactobacillus salivarius ssp. salivarius CECT5713 are not restricted to the large intestine. Eur J Nutr., 51:365-374, 2012. 6. Assuma R, Oates T, Cochran D, Amar S, Graves DT. IL-1 and TNF antagonists inhibit the inflammatory response and bone loss in experimental periodontitis. J Immunol., 160:403-409, 1998. 7. Alkaya B, Laleman I, Keceli S, Ozcelik O, Cenk Haytac M, Teughels W. Clinical effects of probiotics containing Bacillus species on gingivitis: a pilot randomized controlled trial. J Periodont Res 2016; doi:10.1111/jre.12415. 8. Badia R, Brufau MT, Guerrero-Zamora AM, Lizardo R, et al. -Galactomannan and Saccharomyces cerevisiae var. boulardii modulate the immune response against Salmonella enterica serovar Typhimurium in porcine intestinal epithelial and dendritic cells. Clin Vaccine Immunol., 19:368-376, 2012. 9. Bass CC. An effective method of personal oral hygiene. J La State Med Soc., 106:57-73, 1954. 10. Berglundh T, Liljenberg B, Tarkowski A, Lindhe J. The presence of local and circulating autoreactive B cells in patients with advanced periodontitis. J. Clin. Periodontol., 29:281-286, 2002. 11. Bien T, Miller WM, Tonigan J. Brief interventions for alcohol problems: a review. Addiction 1993: 88: 315–336. 12. Bogsan CS, Novaes e Brito RR, Palos Mda C, Mortara RA, et al. B-1 cells are pivotal for in vivo inammatory giant cell formation. Int. J. Exp. Pathol., 86:257-265, 2005. 13. Bogsan CSB, Ferreira L, Maldonado C, Perdigon G, et al. Fermented or unfermented milk using Bifidobacterium animalis subsp. lactis HN019: Technological approach determines the probiotic modulation of mucosal cellular immunity. Food Research International., 64, 283-288, 2014. 14. Caglar E, Cildir SK, Ergeneli S, Sandalli N, Twetman S. Salivary mutans streptococci and lactobacilli levels after ingestion of the probiotic bacterium Lactobacillus reuteri ATCC 55730 by straws or tablets. Acta Odontol Scand., 64:314-318, 2006. 15. Caglar E, Kuscu OO, Cildir SK, Kuvvetli SS, Sandalli N. A probiotic lozenge administered medical device and its effect on salivary mutans streptococci and lactobacilli. Int J Paediatr Dent., 18:35-9, 2008. 16. Castillo NA, Perdigón G, de Moreno de Leblanc A. Oral administration of a probiotic Lactobacillus modulates cytokine production and TLR expression improving the immune response against Salmonella enterica serovar Typhimurium infection in mice. BMC Microbiol., 11:177, 2011. 17. Chatterjee A, Bhattacharya H, Kandwal A. Probiotics in periodontal health and disease. J Indian Soc Periodontol., 15:23-28, 2011. 18. Charles CH, Mostler KM, Bartels LL, Mankodi SM. Comparative antiplaque and antigingivitis effectiveness of a chlorhexidine and an essential oil mouthrinse: 6-month clinical trial. J Clin Periodontol., 31(10):878-84, 2004. 19. Cochran DL. Inflammation and bone loss in periodontal disease. J Periodontol., 79(Suppl):1569-1576, 2008. 20. Commane D, Hughes R, Shortt C, Rowland I. The potential mechanisms involved in the anti-carcinogenic action of probiotics. Mutat Res., 11;591:276-289, 2005. 21. Cosseau C, Devine DA, Dullaghan E, Gardy JL, et al. The commensal Streptococcus salivarius K12 downregulates the innate immune responses of human epithelial cells and promotes host-microbe homeostasis. Infect. Immun., 76, 4163-4175, 2008. 22. Deng J, Li Y, Zhang J, Yang Q. Co-administration of Bacillus subtilis RJGP16 and Lactobacillus salivarius B1 strongly enhances the intestinal mucosal immunity of piglets. Res Vet Sci., 94:62-68, 2013. 23. DiMatteo MR, Giordani PJ, Lepper HS, Croghan TW. Patient adherence and medical treatment outcomes: a meta-analysis. Med Care 2002: 40: 794–811. 24. Dutzan N, Vernal R, Vaque JP, García-Sesnich J, et al. Interleukin-21 expression and its association with proinflammatory cytokines in untreated chronic periodontitis patients. J Periodontol., 83:948-54, 2012. 25. Eke PI, Dye BA, Wei L, Thornton-Evans GO, et al. Prevalence of periodontitis in adults in the United States: 2009 and 2010. J Dent Res., 91:914-20, 2012. 26. Finamore A, Roselli M, Britti MS, Merendino N, Mengheri E. Lactobacillus rhamnosus GG and Bifidobacterium animalis MB5 induce intestinal but not systemic antigen-specific hyporesponsiveness in ovalbumin-immunized rats. J Nutr., 142:375-81, 2012. 27. Flotra L, Gjermo P, Rolla G, Waerhaug J. Side effects of chlorhexidine mouthwashes. Scand J Dent Res., 79(2):119–25, 1971. 28. Gill HS, Rutherfurd KJ, Cross ML. Dietary probiotic supplementation enhances natural killer cell activity in the elderly: An investigation of age-related immunological changes. J. Clin. Immunol., 21:264-271, 2001. 29. Gill HS, Rutherfurd KJ, Prasad J, Gopal PK. Enhancement of natural and acquired immunity by Lactobacillus rhamnosus (HN001), Lactobacillus acidophilus (HN017) and Bifidobacterium lactis (HN019). Br J Nut., 83:167-76, 2000. 30. Gillor O, Etzion A, Riley MA. The dual role of bacteriocins as anti- and probiotics. Appl. Microbiol. Biotechnol., 81:591-606, 2008. 31. Gruner D, Paris S, Schwendicke F. Probiotics for managing caries and periodontitis: Systematic review and meta-analysis. J Dent.,48:16-25, 2016. 32. Gopal PK, Prasad J, Smart J, Gill HS. In vitro adherence properties of Lactobacillus rhamnosus DR20 and Bifidobacterium lactis DR10 strains and their antagonistic activity against an enterotoxigenic Escherichia coli. Int. J. Food Microbiol., 67:207-216, 2001. 33. Gordon DM. The potential of bacteriocin producing probiotics and associated caveats. Future Microbiol., 4:941-943, 2009. 34. Guidelines for the evaluation of probiotics in food: report of a joint FAO/WHO working group on drafting guidelines for the evaluation of probiotics in food. London: FAO/WHO, 2002. 35. Haffajee AD & Socransky SS. Microbial etiological agents of destructive periodontal diseases. Periodontology 2000, 5:78-111, 1994. 36. Hans M, Hans VM. Toll-like receptors and their dual role in periodontitis: a review. J Oral Sci., 53:263-271, 2011. 37. Hallström H, Lindgren S, Yucel-Lindberg T, Dahlén G, et al. Effect of probiotic lozenges on inflammatory reactions and oral biofilm during experimental gingivitis. Acta Odontol Scand.,71(3-4):828-33, 2013. 38. Harini PM, Anegundi RT. Efficacy of a probiotic and chlorhexidine mouth rinses: a short-term clinical study. J Indian Soc Pedod Prev

Dent., 28(3):179-82, 2010. 39. Ierardo G, Bossù M, Tarantino D, Trinchieri V, et al. The arginine-deiminase enzymatic system on gingivitis: preliminary pediatric study. Ann Stomatol (Roma).,1(1):8-13, 2010. 40. Iniesta M, Herrera D, Montero E, Zurbriggen M, et al. Probiotic effects of orally administered Lactobacillus reuteri-containing tablets on the subgingival and salivary microbiota in patients with gingivitis. A randomized clinical trial. J Clin Periodontol., 39(8):736-44, 2012. 41. nce G, Gürsoy H, pçi D, Cakar G, et al. Clinical and biochemical evaluation of lozenges containing Lactobacillus reuteri as an adjunct to non-surgical periodontal therapy in chronic periodontitis. J Periodontol 2015;86:746-54. 42. Jeon SG, Kayama H, Ueda Y, Takahashi T, et al. Probiotic Bifidobacterium breve induces IL-10-producing Tr1 cells in the colon. PLoS Pathog., 8(5):e1002714., 2012. 43. Junick J, Blaut M. Quantification of human fecal bifidobacterium species by use of quantitative real-time PCR analysis targeting the groEL gene. Appl Environ Microbiol., 78:2613-2622, 2012. 44. Karuppaiah RM, Shankar S, Raj SK, Ramesh K, et al. Evaluation of the efficacy of probiotics in plaque reduction and gingival health maintenance among school children - A Randomized Control Trial. J Int Oral Health., 5(5):33-7, 2013. 45. Kragen H. The treatment of inflammatory affections of the oral mucosa with a lactic acid bacterial culturepreparation. Zahnarztl Welt.10;9(11):306-8. 1954 46. Krasse P, Carlsson B, Dahl C, Paulsson A, et al. Decreased gum bleeding and reduced gingivitis by the probiotic Lactobacillus reuteri. Swed Dent J., 30:55-60, 2006. 47. Kottke T, Battista RN, Degriese G, Brekke M. Attributes of successful smoking cessation interventions in medical practice: a meta-analysis of 30 controlled trials. JAMA, 259: 2882–2889, 1988. 48. Liu C, Zhang ZY, Dong K & Guo XK. Adhesion and immunomodulatory effects of Bifidobacterium lactis HN019 on intestinal epithelial cells INT-407. World J Gastroenterol., 14;16:2283-2290, 2010. 49. Lu Q, Samaranayake LP, Darveau RP, Jin L. Expression of human beta-defensin-3 in gingival epithelia. J Periodontal Res., 40:474-481, 2005. 50. Lee JK, Kim SJ, Ko SH, Ouwehand AC, Ma DS. Modulation of the host response by probiotic Lactobacillus brevis CD2 in experimental gingivitis. Oral Dis., 21(6):705-12, 2015. 51. Loe H, Silness J. Periodontal Disease In Pregnancy. I. Prevalence And Severity. Acta Odontol Scand., 21:533-51, 1963. 52. Loe H, Theilade E, Jensen Sb. Experimental Gingivitis In Man. J Periodontol., 36:177-87, 1965. 53. Mariman R, Kremer B, van Erk M, Lagerweij T, et al. Gene expression profiling identifies mechanisms of protection to recurrent trinitrobenzene sulfonic acid colitis mediated by probiotics. Inflamm Bowel Dis., 18:1424-1433, 2012. 54. Meurman JH. Probiotics: do they have a role in oral medicine and dentistry. Eur J Oral Sci., 113:188-196, 2005. 55. Morales A, Carvajal P, Silva N et al. Clinical effects of Lactobacillus Rhamnosus in non-surgical treatment of chronic periodontitis: a randomized placebo-controlled trial with 1-year follow-up. J Periodontol 2016;4:1-12. 56. Monsarrat P, Blaizot A, Kemoun P, Ravaud P, et al. Clinical research activity in periodontal medicine: a systematic mapping of trial registers. Journal Clin Periodontol., 43: 390–400, 2016. 57. Nadkerny PV, Ravishankar PL, Pramod V, Agarwal LA, Bhandari S. A comparative evaluation of the efficacy of probiotic and chlorhexidine mouthrinses on clinical inflammatory parameters of gingivitis: A randomized controlled clinical study. J Indian Soc Periodontol., 19(6):633-9, 2015. 58. Näse L, Hatakka K, Savilahti E, Saxelin M, et al. Effect of long-term consumption of a probiotic bacterium, Lactobacillus rhamnosus GG, in milk on dental caries and caries risk in children. Caries Res., 35:412-420, 2001. 59. Nishitani Y, Tanoue T, Yamada K, Ishida T, et al. Lactococcus lactis subsp. cremoris FC alleviates symptoms of colitis induced by dextran sulfate sodium in mice. Int Immunopharmacol., 9:1444-1451, 2009. 60. Okamoto K, Fujiya M, Nata T, Ueno N, et al. Competence and sporulation factor derived from Bacillus subtilis improves epithelial cell injury in intestinal inflammation via immunomodulation and cytoprotection. Int J Colorectal Dis., 27:1039-1046, 2012. 61. Ouwehand AC, Salvadori B, Fonden R, Mogensen G, et al. Health effects of probiotics and culture-containing dairy products in humans. Bulletin of the International Dairy Federation, 380:4-19, 2003. 62. Oliveira LFF, Salvador SL, Silva PHF, Furlaneto FAC, Figueiredo L, Casarin R, Ervolino E, Palioto DB, Souza SLS, Taba-Jr M, Novaes-Jr AB, Messora MR. Benefits of Bifidobacterium animalis subsp lactis Probiotic in Experimental Periodontitis. J Periodontol.;88(2):197-208, 2016. 63. World Health Organization. Oral health surveys:basic methods. 4° Ed. Geneva:World Health Organization, 1997. 64. Pancer BA, Kott D, Sugai JV, Panagakos FS, et al. Effects of triclosan on host response and microbial biomarkers during experimental gingivitis. J Clin Periodontol., 43: 435–444, 2016. 65. Petersen PE, Ogawa H. Strengthening the Prevention of Periodontal Disease: The WHO Approach. J Periodontol, 76:2187-2193, 2005. 66. Pulikkotil SJ, Nath S. Effects of curcumin on crevicular levels of IL-1 and CCL28 in experimental gingivitis. Aust Dent J., 60(3):317-27, 2015. 67. Prasad J, Smart JB, Gopal PK, Gill HS. Selection and characterization of Lactobacillus and Bifidobacterium strains for use as probiotics. Int. Dairy J., 8:993-1002, 1998. 68. Philippe D, Heupel E, Blum-Sperisen S, Riedel CU. Treatment with Bifidobacterium bifidum 17 partially protects mice from Th1-driven inflammation in a chemically induced model of colitis. Int J Food Microbiol., 149:45-49, 2011. 69. Pugliese LS, Goncalves TO, Popi AF, Mariano M, et al. B-1 lymphocytes differentiate into functional osteoclast-like cells. Immunobiology, 217:336-344, 2012. 70. Quirynen M, Avontroodt P, Peeters W, Pauwels M, et al. Effect of different chlorhexidine formulations in mouthrinses on de novo plaque formation. J Clin Periodontol., 28(12):1127–36, 2001. 71. Rodrigues DM, Sousa AJ, Johnson-Henry KC, Sherman PM, Gareau MG. Probiotics are effective for the prevention and treatment of Citrobacter rodentium-induced colitis in mice. J Infect Dis., 206:99-109, 2012. 72. SB Brasil 2010: Pesquisa Nacional de Saúde Bucal: resultados principais / Ministério da Saúde. Secretaria de Atenção à Saúde. Secretaria de Vigilância em Saúde. – Brasília : Ministério da Saúde, 2012. 73. Salvi GE & Lang NP. Host response modulation in the management of periodontal diseases. J Clin Periodontol., 32 Suppl 6:108-29, 2005. 74. Samuels N1, Grbic JT, Saffer AJ, Wexler ID, Williams RC. Effect of an herbal mouth rinse in preventing periodontal inflammation in an experimental gingivitis model: a pilot study. Compend Contin Educ Dent., 33(3):204-6, 208-11, 2012. 75. Sazawal S, Dhingra U, Hiremath G, Sarkar A, et al. Effects of bifidobacterium lactis HN019 and prebiotic oligosaccharide added to milk on iron status, anemia, and growth among children 1 to 4 years old. J. pediatr. gastroenterol. nutr., 51:341-346, 2010. 76. Scheres N, Laine ML, de Vries TJ, Everts V, van Winkelhoff AJ. Gingival and periodontal ligament fibroblasts differ in their inflammatory response to viable Porphyromonas gingivalis. J Periodontal Res., 45:262-70, 2010. 77. Shimauchi H, Mayanagi G, Nakaya S, Minamibuchi M, et al. Improvement of periodontal condition by probiotics with Lactobacillus salivarius WB21: a randomized, double-blind, placebo-controlled study. J Clin Periodontol., 35:897-905, 2008. 78. Slawik S1, Staufenbiel I, Schilke R, Nicksch S, et al. Probiotics affect the clinical inflammatory parameters of experimental gingivitis in humans. Eur J Clin Nutr., 65(7):857-63, 2011. 79. Shu Q, Lin H, Rutherfurd KJ, Fenwick SG, et al. Dietary Bifidobacterium lactis (HN019) enhances resistance to oral Salmonella typhimurium infection in mice. Microbiol. Immunol., 44:213-222, 2000. 80. Sliepen I, Van Damme J, Van Essche M, Loozen G, et al. Microbial interactions influence inflammatory host cell responses. J Dent Res., 88:1026-1030, 2009. 81. Socransky SS, Haffajee AD, Smith C, Martin L, et al. Use of checkerboard DNA-DNA hybridization to study complex microbial ecosystems. Oral Microbiol Immunol., 19:352-62, 2004. 82. Sookkhee S, Chulasiri M, Prachyabrued W. Lactic acid bacteria from healthy oral cavity of Thai volunteers: inhibition of oral pathogens. J. Appl. Microbiol., 90:172–179, 2001. 83. Staab B, Eick S, Knöfler G, Jentsch H. The influence of a probiotic milk drink on the development of gingivitis: a pilot study. J Clin Periodontol., 36:850-856, 2009. 84. Stamatova I & Meurman JH. Probiotics and periodontal Disease. Periodontol 2000, 51:141-151, 2009. 85. Stelin S, Ramakrishan H, Talwar A, Arun KV, Kumar TS. Immunohistological analysis of CD1a langerhans cells and CD57 natural killer cells in healthy and diseased human gingival tissue: A comparative study. J Indian Soc Periodontol., 13:150-154, 2009. 86. Teughels W, Newman MG, Coucke W, Haffajee AD, et al. Guiding periodontal pocket recolonization: a proof of concept. J Dent Res., 86:1078–1082, 2007. 87. Teughels W, Van Essche M, Sliepen I & Quirynen M. Probiotics and oral health. Periodontol 2000, 48:111-1147, 2008. 88. Tekce M, Ince G, Gursoy H, et al. Clinical and microbiological effects of probiotic lozenges in the treatment of chronic periodontitis: a 1-year follow-up study. J Clin Periodontol 2015;42:363–72. 89. Tonetti MS, Chapple IL. Working Group 3 of Seventh European Workshop on Periodontology. Biological approaches to the development of novel periodontal therapies—consensus of the Seventh European Workshop on Periodontology. J Clin Periodontol., 38 Suppl 11:114-118, 2011. 90. Tonetti MS, Eickholz P, Loos BG, Papapanou P, et al. Principles in prevention of periodontal diseases: Consensus report of group 1 of the 11th European Workshop on Periodontology on effective prevention of periodontal and peri-implant diseases. J Clin Periodontol., 42 Suppl 16:S5-11, 2015. 91. Tsubura S, Mizunuma H, Ishikawa S, Oyake I, et al. The effect of Baccillus subtilis Mouth rinsing in patients with periodontitis. Eur J Clin Microbiol Infect Dis., 28:1353-1356, 2009. 92. Turesky S, Gilmore ND, Glickman I. Reduced plaque formation by the chloromethyl analogue of victamine C. J Periodontol., 41(1):41-3, 1970. 93. Twetman S, Derawi B, Keller M, Ekstrand K, et al. Short–term effect of chewing gums containing

probiotic Lactobacillus reuteri on the levels of inflammatory mediators in gingival crevicular fluid. Acta Odontol Scand., 67:19-24, 2009. 94. Van der Ouderaa EJG: Anti-plaque agents. Rationale and prospects for prevention of gingivitis and periodontal disease. J Clin Periodontol., 18: 447-454, 1991. 95. Vivekananda MR, Vandana KL, Bhat KG. Effect of the probiotic Lactobacilli reuteri (Prodentis) in the management of periodontal disease: a preliminary randomized clinical trial. J Oral Microbiol. 2:5344, 2010. 96. Zhang G, Chen R, Rudney JD. Streptococcus cristatus attenuates Fusobacterium nucleatum-induced interleukin-8 expression in oral epithelial cells. J. Periodontal. Res., 43, 408-416, 2008. 97. Zhou JS & Gill HS. Immunostimulatory probiotic Lactobacillus rhamnosus HN001 and Bifidobacterium lactis HN019 do not induce pathological inflammation in mouse model of experimental autoimmune thyroiditis. Int. J. Food Microbiol., 103:97-104, 2005. 98. Zhu D, Chen X, Wu J, Ju Y, et al. Effect of perioperative intestinal probiotics on intestinal flora and immune function in patients with colorectal cancer. Nan Fang Yi Ke Da Xue Xue Bao, 32:1190-1193, 2012.

Upload de Documentos

Arquivo Anexos:

Tipo	Arquivo
Comprovante de Recepção	PB_COMPROVANTE_RECEPCAO_897890.pdf
Folha de Rosto	FOLHA_de_ROSTO1.pdf
Declaração de Instituição e Infraestrutura	aut_infrDCTMF.pdf
TCLE / Termos de Assentimento / Justificativa de Ausência	TCLE.docx
Outros	Questionario.docx
Declaração de Instituição e Infraestrutura	aut_infrC.pdf
Outros	dec_part_pesq.doc
Outros	Flavia_Formulario_para_EMENDA_Projeto_Gengivite.pdf
Projeto Detalhado / Brochura Investigador	PROJETO_DE_PESQUISA.pdf
Comprovante de Recepção	PB_COMPROVANTE_RECEPCAO_964740.pdf

Finalizar

Manter sigilo da integra do projeto de pesquisa: Sim

Prazo: 2 anos

Justificativa da Emenda:

Todos os dados clínicos dos pacientes do presente projeto de pesquisa já foram coletados e os procedimentos clínicos do estudo já foram finalizados. Foram coletadas amostras de biofilme dental para serem submetidas à análise microbiológica. A análise microbiológica que havia sido submetida ao Comitê de Ética em Pesquisa tinha como objetivo verificar a composição microbiológica do biofilme por meio de checkerboard DNA-DNA hybridization e a quantificação absoluta de Bifidobacterium animalis subsp. lactis HN019 no biofilme por qPCR. Porém, surgiu a oportunidade de realizar uma análise microbiológica mais completa das amostras de biofilme, utilizando a tecnologia de sequenciamento de nova geração. Declaro que isso não irá influenciar o atendimento aos pacientes ou a coleta de suas amostras, os quais já foram realizados.
