# Supplementary material for: Microbiological profile of patients with generalized gingivitis undergoing periodontal therapy and administration of Bifidobacterium animalis subsp. lactis HN019: A randomized clinical trial
Source: PLoS One. 2024 Nov 11;19(11):e0310529. doi: 10.1371/journal.pone.0310529 (PMC11554181; doi:10.1371/journal.pone.0310529)
Supplement: S2 Appendix — English version. (PDF) [file pone.0310529.s004.pdf]

S2 Appendix. Research project submitted for approval by the ethics committee. English version.

**Research Project:**
"Effects of probiotic therapy on gingivitis: study of the clinical, microbiological and immunological profile of the host response."

Preliminary Information

Principal

CPF/Document: 831.791.271-04	Name: Flávia Aparecida Chaves Furlaneto Messora
Phone: 16982020181	E-mail: flafurlaneto@hotmail.com

Proposing Institution

CNPJ: 63.025.530/0086-01	Name of Institution: University of Sao Paulo

Is this amendment submission exclusive to your Coordinating Center?

The amendment is exclusive to your Coordinating Center, so the changes made to your project as a result of the amendment will NOT be replicated in the linked Participating Centers and in the Ethics Committees of the Co-participating Institutions, when they are approved.

Is it an international study? No

■ **Assistants**

CPF/Document	Name
015.231.336-27	Renata Cardoso

■ **Research Team**

CPF/Document	Name
042.597.906-76	Michel Reis Messora
034.019.661-03	PEDRO HENRIQUE FELIX SILVA
054.381.405-02	RAFAEL MORAES CHAVES SANTOS

Study Area

Broad Areas of Knowledge (CNPq)

- Major Area 4. Health Sciences

Main Purpose of the Study (WHO)

- Clinical

Public Title of **the Research:** "Effects of probiotic therapy on gingivitis: study of the clinical, microbiological and immunological profile of the host response."

Public Contact

CPF/Document	Name	Telephone	E-mail
831.791.271-04	Flávia Aparecida Chaves Furlaneto Messora	16982020181	flafurlaneto@hotmail.com

Scientific Contact: Flávia Aparecida Chaves Furlaneto Messora

Study Design / Financial Support

Study Design: Intervention/Experimental

Health conditions or problems

Health condition or problem
Gingivitis

General Descriptors for Health Conditions

ICD1-10: International Classification of Diseases

ICD code	CID Description
K05	Gingivitis and periodontal disease

DeCS:Health Science Descriptors

DECS code	DECS Description
C07.465.714.258.480	Gingivitis

Specific Descriptors for Health Conditions

ICD1-10: International Classification of Diseases

ICD code	CID Description
K05	Gingivitis and periodontal disease

DeCS:Health Science Descriptors

DECS code	DECS Description
C07.465.714.258.480	Gingivitis

Type of Intervention: Experimental

Nature of Intervention

- Procedure/operative/surgery

Intervention Descriptors

List of ICDs

ICD code	CID Description
K05	Gingivitis and periodontal disease

List of CEDS

DECS code	DECS Description
C07.465.714.258.480	Gingivitis

Phase

- Phase 1

Design:

Randomized, double-blind clinical study.

Financial support

CNPJ	Name	E-mail	Telephone	Type
				Own financing

Key words

Key words
Gingivitis
Probiotic
Chemical control

Summary:

Considering the limitations of oral hygiene in the general population and also the involvement of various mechanisms relating to the host's innate and adaptive immune system in the pathogenesis of periodontal diseases, the use of probiotics as a new adjuvant therapy for reducing plaque and gingivitis has aroused the interest of the dental scientific community, since they can modulate the host's immune-inflammatory response and modify the bacterial environment. The purpose of this randomized, double-blind, placebo-controlled clinical study will be to evaluate the effects of oral administration of the probiotic strain Bifidobacterium animalis subsp. lactis HN019 (B. lactis HN019) in patients with generalized gingivitis. Sixty individuals with periodontal disease (generalized gingivitis) will receive the gold standard periodontal treatment for gingivitis (prophylaxis and/or supragingival scraping) 1 week (-1s) before the start of the study. Patients will be instructed, immediately after their first visit (baseline), to consume tablets containing B. lactis HN019 (test group) or placebo (control group) once a day for 56 days.
Clinical periodontal, immunological and microbiological parameters will be assessed at baseline (pre-intervention period) and 28 and 56 days after the start of probiotic or placebo administration. The clinical parameters assessed will be: plaque index, gingival index, gingival bleeding index, clinical probing depth, clinical attachment level, bleeding on probing. Immunological parameters will be analyzed using enzyme immunoassays (LuminexTM xMAP®) to check the levels of the cytokines IL-1, IL-1, IL-8, RANTES, MCP-1 and MIP-1 present in the gingival crevicular fluid. Supragingival and subgingival bacterial plaques will be collected to analyze the microbiological composition of the biofilm using checkerboard DNA-DNA hybridization and to detect and quantify B. lactis HN019 in the biofilm using polymerase chain reaction (qPCR). The data obtained will be statistically analyzed (p<0.05).Keywords: Gingivitis; Probiotic; Chemical control.

Introduction:

1. QUALIFICATION OF THE MAIN PROBLEM TO BE ADDRESSED1.1 Periodontal diseases (PDs): treatment perspectives Periodontal diseases (PDs) comprise a group of inflammatory diseases, including gingivitis and periodontitis, which affect the protective and supporting tissues of the teeth and can lead to tooth loss, affecting a large part of the population. The epidemiological survey SB Brasil 2010 showed that gingival bleeding gradually increases with age, affecting half of all individuals between the ages of 35 and 44. In a recent epidemiological survey carried out in the United States, it was shown that one in two Americans aged 30 and over has periodontitis (EKE et al., 2012).Bacterial biofilm is the primary etiological factor for the onset of gingival inflammation and subsequent destruction of periodontal tissues (HAFFAJEE & SOCRANSKY, 1994). The host's immunoinflammatory response to bacterial aggression is responsible for the pathogenesis and progression of the disease from gingivitis to periodontitis (SALVI & LANG, 2005). Acquired and environmental risk factors (e.g. diabetes mellitus, smoking and stress), as well as some genetically transmitted characteristics (e.g. gene polymorphisms for Interleukin [IL]-1) can accentuate the inflammatory response resulting from bacterial aggression and, eventually,susceptibility to PDs (SALVI & LANG, 2005). PDs have been associated with approximately 57 systemic disorders, such as diabetes mellitus, coronary heart disease, adverse effects on pregnancy, breast cancer, among others (AMÓDIO et al., 2014; MONSARRAT et al., 2016). These systemic effects have led to the understanding that finding an effective treatment for chronic inflammatory gum disease is vital not only for the prevention of tooth loss, but also as part of the patient's overall health care (SAMUELS et al., 2012; MONSARRAT et al., 2016).Mechanical removal of bacterial plaque is the most effective method for controlling gingivitis, which in a study on the development of gingivitis, inflammation of the gingival tissue was compatible with the accumulation of biofilm (LOE et al., 1965).However, the general population does not carry out adequate plaque control (PETERSEN & OGAWA, 2005). Strategies based on professional recommendations, i.e. demonstration methods and dialog with patients on how to improve their oral hygiene, do not lead to the expected results (KOTTKE et al., 1988; BIEN et al., 1993). It has been shown that approximately 30 to 60% of the information provided by professionals is forgotten within an hour and that 50% of health recommendations are not followed (DIMATTEO et al., 2012). Based on the consensus of the 11th European Workshop on Periodontology on effective prevention of periodontal and peri-implant diseases, it is known that the patient needs to acquire positive attitudes for behavioral change, and that achieving real behavioral change in relation to adequate plaque control is one of the most challenging responsibilities of professionals, and can take a long time (TONETTI et al., 2015), The fact that the general population does not undergo adequate oral hygiene, coupled with current knowledge about the influence of PDs on the individual's systemic health, has prompted the development of agents to control biofilm formation and/or to modulate the host's immunoinflammatory response to plaque (VAN DER OUDERAA, 1991; PULIKKOTIL & NATH, 2015). The use of antimicrobial mouthwashes with chlorhexidine and essential oils has been shown to be the most suitable for promoting long-term plaque and gingivitis control (CHARLES et al., 2004). However, the adverse effects of chlorhexidine mouthwashes, such as altered taste, increased supragingival calculus formation, soft tissue lesions in young patients, allergic responses and staining of teeth and soft tissues, raise questions about their efficacy for long-term use (FLOTRA et al., 1971; QUIRYNEN et al., 2001). Currently,there is a great search for an agent that has the same properties as chlorhexidine in inhibiting the development of gingivitis, but without its adverse effects, and can be used for a long period of time. possibility of modulating the host response in PDs by interfering with the expression of pro-inflammatory cytokines was demonstrated in a study carried out by Assuma et al. (1998)this study,periodontitis was induced by placing bandages infected with Porphyromonas gingivalis in primates. Local injection of antagonists of some pro-inflammatory interleukins reduced the recruitment of inflammatory cells by 80%, the formation of osteoclasts by 67% and bone loss by 60% when compared to control teeth that did not receive local injection of the antagonists (ASSUMA et al., 1998). In this context, considering the limitations of oral hygiene and also the participation of various mechanisms relating to the host's innate and adaptive immune system in the pathogenesis of PDs, such as the release of pro-inflammatory and anti-inflammatory cytokines (COCHRAN, 2008; SCHERES et al., 2010; DUTZAN et al., 2012), the production of beta-defensins (BD) (LU et al., 2005), activation of Toll-like receptors (TLR) (HANS & HANS, 2011) and infiltration of natural killer cells (STELIN et al., 2009), the use of probiotics as a new adjuvant therapy to reduce plaque and gingivitis has aroused the interest of the dental scientific community, since they can modulate the host's immunoinflammatory response and modify the bacterial environment. Recently, a systematic review and meta-analysis demonstrated that the current scientific evidence supports the use of probiotics in the therapeutic approach to gingivitis and periodontitis (GRUNER et al., 2016).1.2, Probiotics: a new approach to the treatment of PDsProbiotic therapy can provide advantages that are not observed when antibiotics and/or antiseptics are used for chemical plaque control (KARUPPAIAH et al., 2013), as it does not promote bacterial resistance and can naturally interfere with the host's local and systemic immunoinflammatory response. A recent "proof of concept" study demonstrated that a mixture of Streptococcus species applied to the teeth of dogs with periodontitis, as an adjunct therapy to root scraping and root planing (RAR), delayed the recolonization of periodontal pathogens and reduced inflammation (TEUGHELS et al., 2007).Probiotics are defined as live microorganisms, mainly bacteria, that are safe for consumption and capable of producing beneficial effects on the host's health when ingested in sufficient quantities (FAO/WHO, 2002). The consumption of probiotics can boost the host's immune system, as well as act in the prevention/treatment of certain diseases and/or discomforts in humans, such as diarrhea due to infection with Helicobacter pylori, poor digestion of lactose and irritable bowel syndrome. In addition, they act to prevent colon and bladder cancer, control cholesterol levels and high blood pressure, protect against urinary and respiratory tract infections and suppress allergies (OUWEHAND et al., 2003; AMROUCHE, 2005; COMMANE et

al., 2005). Probiotics are generally regulated as dietary supplements and marketed to improve or maintain health (TSUBURA et al., 2009). The main microorganisms used for probiotic purposes are bacteria of the genus Lactobacillus and Bifidobacterium (TEUGHELS et al., 2008).first study using probiotics and targeting the oral cavity was carried out over 60 years ago as a treatment for mucosal inflammation (KRAGEN, 1954). But only recently has the oral cavity been suggested as a relevant target for probiotic applications (MEURMAN, 2005). Probiotics have been evaluated mainly in the control of dental caries, as they can reduce the levels of Streptococcus mutans in saliva (NÄSE et al., 2001; AHOLA et al., 2002; CAGLAR et al., 2006; CAGLAR et al., 2008). The mechanisms of action of probiotics in the oral cavity appear to be analogous to those described for the balance of intestinal microflora (HAUKIOJA, 2010). These mechanisms could be an alternative not only for controlling dental caries, but also for treating gingivitis and periodontitis. Microorganisms used for probiotic purposes can have a direct effect on periodontal pathogens, affecting their growth, adhesion and colonization (STAMATOVA & MEURMAN, 2009). Probiotic bacteria can produce various components that act as antimicrobial agents, such as lactic acid, hydrogen peroxide, bacteriocins and bacteriocin-like inhibitory substances (GILLOR et al., 2008; GORDON, 2009; OELSCHLAEGER, 2010). Sookkhee et al. (2001) isolated lactic acid-producing bacteria from the oral cavity of healthy Thai volunteers and showed that they developed antimicrobial activity against Porphyromonas gingivalis and Streptococcus mutans. Van Hoogmoed et al. (2000) observed that a biosurfactant produced by Streptococcus mitis is capable of reducing the adhesion of S. mutans and various periodontopathogens. Another mechanism suggested to explain the action of probiotics in the treatment of PDs refers to the modulation of the host's immunoinflammatory response (STAMATOVA & MEURMAN, 2009). Some studies have shown that certain probiotic species can attenuate the expression of IL-8 induced by periodontopathogens in oral epithelial cells (COSSEAU et al., 2008; ZHANG et al., 2008; SLIEPEN et al., 2009) and reduce the levels of pro-inflammatory cytokines (IL-8, IL- and Tumor Necrosis Factor [TNF]-) in gingival crevicular fluid (TWETMAN et al., 2009). Shimauchi et al. (2008) found that probiotic consumption significantly decreased levels of salivary lactoferrin, a protein indicative of periodontal inflammation, in highlysusceptibleindividuals.Staab et al. (2009) demonstrated that probiotic intake can reduce polymorphonuclear elastase activity, as well as myeloperoxidase and matrix metalloproteinase-3 levels in the gingival crevicular fluid of individuals with gingivitis. The studies (SHIMAUCHI et al., 2008; STAAB et al., 2009; IERARDO et al., 2010; SLAWIK et al. 2011; INIESTA et al., 2012; HALLSTROM et al., 2013; LEE et al., 2015; NCE et al., 2015; TEKCE et al., 2015; NADKERNY et al., 2016; MORALES et al., 2016) that have investigated the effects of probiotics on PDs to date have mainly used microorganisms from the Lactobacillus genus. However, other potential probiotics deserve to be investigated. The agent Bifidobacterium animalis subsp. lactis HN019, which originates from dairy products, is considered a potential probiotic and has the ability to resist the action of bile and very acidic pH (PRASAD et al., 1998). This strain is also capable of adhering in high quantities to different types of intestinal epithelial cells (GOPAL et al., 2001) and has interesting immunomodulatory properties (GILL et al., 2000). Human studies have shown that B. lactis HN019 was able to improve the innate immune response of elderly and middle-aged individuals (GILL et al., 2001), 2001), increased the cytotoxic activity of natural killer cells and the phagocytic activity of peripheral monocytes, with this activity persisting for up to six weeks after the probiotic was discontinued (ZHOU & GILL, 2005), reduced iron deficiency in pre-school children, resulting in weight gain (SAZAWAL et al., 2010) and promoted the protection of enterocytes against acute infection (LIU et al., 2010). An animal study showed that 80% of mice treated with B. lactis HN019 daily for one week remained alive for three weeks after being infected with Salmonella typhimurium. In animals that did not consume the probiotic, the mortality rate was 93% (SHU et al., 2000). In a study carried out by our group, it was found that ingesting the probiotic

B. lactis HN019 by mice promotes a reversal in the ratio of B-1/B-2 cells (BOGSAN et al., 2014). B cells comprise two distinct subtypes: conventional B lymphocytes (or B-2) and B-1 lymphocytes. The increased presence of B-1 cells in individuals with periodontitis (5 to 6 times more than in individuals without periodontitis) may explain the greater destruction of periodontal tissues observed in these patients (BERGLUNDH et al., 2002). There are reports that the modulation of bone resorption in vivo and the differentiation of osteoclast-like cells may be promoted by the interaction of B-1 cells (PUGLIESE et al., 2012), since multinucleated cells depend on B-1 cells for their formation (BOGSAN et al., 2005). From the knowledge we have to date, our group recently carried out the first study with the administration of B. lactis in PDs. The effects of local administration of B. lactis HN019 were analyzed in rats with ligature-induced periodontitis (OLIVEIRA et al., 2016). It was observed that treated animals had higher proportions of Actinomyces and Streptococcus-like species and lower proportions of Eikenella corrodens and Prevotella intermedia-like species than untreated animals. In addition, there were higher expressions of OPG and beta-defensins and lower expressions of IL-1 and RANKL in the animals given B. lactis HN019 than in the untreated animals. All these results obtained with the use of B. lactis HN019 suggest that this probiotic strain could be very useful in the treatment of PDs. There are still few clinical studies evaluating the effects of probiotics on gingivitis. These studies have been carried out in models of experimental gingivitis with the suspension of oral hygiene on all the subjects' teeth (STAAB et al., 2009; LEE et al., 2015) or on just a few teeth (HALLSTROM et al., 2013; SLAWIK et al., 2011) and also with the administration of probiotics in patients with already installed gingivitis (TWETMAN et al., 2009; KRASSE et al., 2006; IERARDO et al., 2010; INIESTA et al., 2012).Clinical studies show that the mechanical removal of bacterial plaque leads to the complete resolution of gingival inflammation, i.e. clinically healthy gums, with no deleterious effects for patients, within a few days of starting proper oral hygiene procedures (LOE et al., 1965; PANCER et al., 2016). Among the clinical studies that have evaluated the effects of probiotics on gingivitis, there are none using the strain

B. lactis HN019. In general, clinical studies have shown that the use of probiotics can promote a significant reduction in periodontopathogens (INIESTA et al., 2012), improve gingival clinical parameters (KRASSE et al., 2006; HARINI & ANEGUNDI, 2010; IERARDO et al., 2010; LEE et al., 2015, NADKERNY et al., 2015), 2015), reduce inflammatory markers in gingival crevicular fluid (GFCF) (STAAB et al., 2009; TWETMAN et al., 2009; LEE et al., 2015) or saliva (SHIMAUCHI et al., 2008; IERARDO et al., 2010) and inhibit the development of gingivitis (STAAB et al., 2009; SLAWIK et al., 2011; KARUPPAIAH et al., 2013; LEE et al., 2015). In the study by Hallstrom et al. (2013), the authors were unable to demonstrate a protective effect of Lactobacillus administration on the inflammatory pattern or microbiological composition of the supragingival plaque of patients with experimental gingivitis.However, as the authors themselves point out, more clinical studies are needed to elucidate the role of probiotics in the treatment of gingivitis, periodontitis and even peri-implant mucositis. It is also important to note that there is still a large gap in the literature on the real mechanisms of action of probiotics for the treatment and prevention of PDs, especially with regard to their role in modulating the host's periodontal immunoinflammatory response. As similar immunoinflammatory mechanisms, which determine the host's health-disease process, occur in periodontal tissues and in the intestinal mucosa, it is believed that the immunoinflammatory action of probiotics in the oral cavity is analogous to that described in the intestinal mucosa. Recent studies have shown that probiotics can: potentiate host immunity through an increase in beta-defensin (BD) expression in the intestinal mucosa (DENG et al., 2013) likewise Oliveira et al; (2016) obtained similar results in the oral epithelium, reinforce the intestinal epithelial barrier by increasing TLR expression (CASTILLO et al., 2011), reduce intestinal inflammation by interfering with the expression of cells positive for Differentiation Group (CD)-4, CD-8, CD-57 and Foxhead Box P3 (Foxp3) or the Nuclear Factor Kappa-beta pathway (JEON et al., 2012;NISHITANI et al., 2009), decrease the production of pro-inflammatory cytokines (IL-1, IL-4, IL-8, IL-17, TNF-, Interferon [INF]-, Monocyte Chemotactic Protein [MCP-1] and Macrophage Colony Stimulating Factor [M-CSF]) (PHILIPPE et al., 2011; ZHU et al., 2012; RODRIGUES et al., 2012; OKAMOTO et al., 2012; MARIMAN et al., 2012; BADIA et al., 2012) and increase the production of anti-inflammatory cytokines (IL-10 and Transforming Growth Factor [TGF]-) (FINAMORE et al., 2012; RODRIGUES et al., 2012; ARRIBAS et al., 2012). Thus, further studies are essential to explore these mechanisms of action of probiotics in periodontal tissues. A

Probiotic therapy is still at an "infant stage" for periodontal health care, but it undoubtedly opens a door to the new paradigm of treating PDs in a nanomolecular way (CHATTERJEE et al., 2011). Many previous clinical studies evaluating the use of probiotics in PDs have produced inconsistent results, which can be attributed to differences in the strains used and the design of the studies, which can allow for bias (LEE et al., 2015). Therefore, new studies are essential to better elucidate the role of beneficial bacteria in the oral cavity, identify new species of bacteria, establish times and new administration vehicles and determine the real clinical significance of this therapy (TEUGHELS et al., 2008; STAMATOVA & MEURMAN, 2009; TONETTI & CHAPPLE, 2011).

Hypothesis:

It is hoped that the use of probiotics (test group) will potentiate the effects of gold standard periodontal therapy, leading to better results in terms of clinical periodontal, microbiological and immunological parameters when compared to the control group.

Primary Objective:

To evaluate the effects of oral administration of the probiotic strain B. lactis HN019 as an adjuvant to the treatment o f gingivitis in humans.

Secondary Objective:

In patients with generalized gingivitis, with oral administration of probiotics or placebo, evaluate:
Clinical parameters: plaque index, gingival index, gingival bleeding index, clinical probing depth, clinical insertion level, bleeding on probing;
Levels of the cytokines IL-1, IL-1, IL-8, RANTES, MCP-1 and MIP-1 present in gingival crevicular fluid, using enzyme immunoassays (LuminexTM xMAP®);
Biofilm microbiota, by checkerboard DNA-DNA hybridization;
Detection and quantification of Bifidobacterium animalis subsp. lactis HN019 in biofilm by Polymerase Chain Reaction (qPCR).

Proposed Methodology:

Consent for the research (Free and Informed Consent Form), submission of the project to the CEP; Sample calculation (total = 60, n = 30); Selection of patients (FORP-USP Postgraduate Clinic); All patients will undergo a full-mouth periapical radiographic examination. The volunteers will be grouped into an oral hygiene program (OHP) according to their specific needs.
In this program, patients will receive instructions for effective plaque self-control, including information on the Bass technique (BASS, 1954) and interproximal cleaning with dental floss. They will also be encouraged to brush the back of the tongue once a day and will receive a toothbrush and the same toothpaste to be used throughout the trial period (Colgate Total®, Anakol Ind. Com. Ltda -Kolynos do Brasil - Colgate Palmolive Co., São Bernardo do Campo, SP, Brazil). Before starting the study, the selected individuals will be identified by a numerical code. According to a random numerical table generated by a computer program, the study coordinator will allocate each patient to one of the following experimental groups: control (placebo) or test (probiotic therapy). The volunteers will not know which experimental group they belong to. One week before the initial collections (-1s), all patients included in the study will receive prophylaxis and/or supragingival scraping, as necessary. On day 0, all subjects will receive tablets with the same composition, texture, shape and flavor, except for the presence of the probiotic. In the test group, the 10 mg tablets will contain 109 colony-forming units (CFUs) of Bifidobacterium animalis subsp. lactis HN019. Subjects will be instructed, immediately after their first consultation (baseline), to consume the tablet once a day for 8 weeks. They will be instructed to always consume the gum after the last brushing, before going to bed, letting the gum dissolve in the mouth.During the 56 days in which they consume the tablets, the volunteers will also be instructed to keep the tablets in the fridge, not to consume any other probiotic product and not to use any chemical plaque control product, with the exception of dentifrice. After 28 days of starting daily intake of Bifidobacterium animalis subsp. lactis HN019, the patients will be instructed to return for collection of new clinical data, and they will have to spend 8 hours prior to the examination without any hygiene measures, with the exception of the tablets, (AYALA., et al.; 2016). Participants will be instructed to maintain their oral hygiene routine throughout the study. Clinical periodontal, immunological and microbiological parameters will be assessed in three periods: at baseline (pre-intervention period), 28 days after the start of probiotic or placebo administration and 56 days after the start of probiotic or placebo administration. Clinical periodontal examinations will be carried out by a single trained and calibrated examiner, who will be unaware of the experimental groups to which each volunteer belongs. The examiners of the immunological and microbiological evaluations will also be blind to the experimental groups of the samples analyzed. The probiotic and non-probiotic (placebo) tablets will be prepared by a compounding pharmacy and both will have the same format and will be packaged in identical bottles. The tablets will be sent to the study coordinator, who will mark each patient's numerical code on a set of 56 tablets (the amount to be consumed by each individual over 8 weeks), according to the experimental group to which they belong. The coded packs will be sent to the study's clinical examiner, who will distribute them to the patients and at no time will have any information about their contents. (See the rest in the project file).

Inclusion Criteria:

Inclusion criteria will be: (1) systemically healthy individuals, (2) presence of gingival inflammation in more than 30% of the sites (GI=1), (3) presence of 28 20 fully erupted permanent teeth, excluding third molars and teeth indicated for exodontia, (4) willingness to adhere to the study protocol.

Exclusion Criteria:

Exclusion criteria for the present study: (1) pregnant or lactating women, (2) systemic involvement that could interfere with the results of the study (e.g: diabetes mellitus, immunological disorders), (3) intake of antimicrobials and/or anti-inflammatories in the last 6 months and probiotics or oral antiseptics in the last month, (4) use of medication with gingival implications, (5) history or presence of periodontitis, (6) presence of non-plaque-induced gum disease, (7) known allergies to the experimental materials, (8) presence of orthodontic appliances, (9) extensive prosthetic involvement, (10) smoking, (11) legally incapacitated patients.

Risks:

The risks present refer to the discomfort the patient will experience when undergoing the gold standard periodontal treatment for gingivitis (prophylaxis and/or supragingival scraping). The use of tablets containing placebo or probiotics will not pose any risk to the patient's health.

Benefits:

Balancing the intestinal flora

Data Analysis Methodology:

The normality and homoscedasticity of the data obtained will be verified. Inter- and intra-group comparisons at different time intervals will be carried out using appropriate parametric or non-parametric tests. A significance level of 5% will be used for all statistical analyses. All calculations will be carried out using SPSS software (SPSS, Chicago IL, USA).

Primary outcome:

Gingival Bleeding Index

Secondary outcome:

Turesky Plaque IndexGingival Index (LOE & SILNESS, 1963)Clinical probing depth (mm)Clinical insertion level (mm)Bleeding on probing, assessed dichotomously (AINAMO & BAY, 1975)Microbiological composition of the biofilm by checkerboard DNA-DNA hybridizationAbsolute quantification of Bifidobacterium animalis subsp. lactis HN019 in the biofilm by qPCRQuantification of cytokines (pg/l) IL-1, IL-1, IL-8, RANTES, MCP-1, MIP-1

Sample size in Brazil: 60

Recruitment countries

Country of origin of the study	Country	No. of survey participants
Yes	BRAZIL	60

Other information

Will secondary data sources (medical records, demographic data, etc.) be used?

No

Report the number of individuals personally approached, recruited, or who will undergo some kind of intervention at this research center:

60

Groups into which the research participants at this center will be divided

Group ID	No. of individuals	Interventions to be carried out
Control (gold standard periodontal therapy)	30	Prophylaxis and/or supragingival scraping, consumption of placebo tablets and additional periodontal treatment (if necessary) at the end of the experiment.
Test (gold standard periodontal therapy and probiotic)	30	Prophylaxis and/or supragingival scraping, consumption of probiotic tablets and additional periodontal treatment (if necessary) at the end of the experiment.

Is the Study Multicentric in Brazil?

No

Do you propose waiving the ICF?

No

Will samples be retained for bank storage?

No

Execution schedule

Stage identification	Start (DD/MM/YYYY)	End (DD/MM/YYYY)
Preparation of abstracts for presentation at national and international conferences	01/05/2019	30/08/2019
Immunological analysis	01/08/2018	01/01/2019
Clinical, immunological and microbiological monitoring	01/08/2018	01/01/2019
Final report on the activities carried out	01/05/2019	30/08/2019
Statistical analysis of the data obtained	01/02/2019	30/04/2019
Data collection	01/03/2018	02/07/2018
Clinical, immunological and microbiological monitoring	01/05/2018	02/07/2018
Patient screening-Partial procurement of consumables	01/08/2017	02/07/2018
Preparation of abstracts for presentation at national and international conferences	28/02/2019	31/05/2019
Preparation of manuscripts for publication in journals with a selective editorial policy classified A1 in the CAPES Qualis system.	01/05/2019	30/08/2019

Partial activity report - 1	02/07/2018	31/07/2018
Microbiological analysis	01/08/2018	01/01/2019

Financial Budget

Budget identification	Type	Value in Reais (R$)
Gracey Curets	Costs	R$ 1.500,00
Toothpaste (Colgate)	Costs	R$ 500,00
Consumables (paper, xerox, printing, clinical, laboratory, etc.)	Costs	R$ 5.000,00
PCR	Costs	R$ 3.500,00
Clinical Periodontal Probes	Costs	R$ 500,00
Toothbrushes (Colgate)	Costs	R$ 800,00
Checkboard	Costs	R$ 30.000,00
Luminex	Costs	R$ 30.000,00
Total in R$		R$ 71.800,00

Bibliography:

5. BIBLIOGRAPHICAL REFERENCES 1. Ahola AJ, Yli-Knuuttila H, Suomalainen T, Poussa T, et al. Short-term consumption of probiotic-containing cheese and its effect on dental caries risk factor. Arch Oral Biol., 47:799-804, 2002. 2. Ainamo J & Bay I. Periodontal indexes for and in practice. Tandlaegebladet, 80:149-52, 1975. 3. Amódio J, Palioto DB, Carrara HH, Tiezzi DG, et al. Oral health after breast cancer treatment in postmenopausal women. Clinics (Sao Paulo), 69(10):706-8, 2014. 4. Amrouche T. Contribution to the study of the immunomodulatory power of bifidobacteria: in vitro analysis and ex vivo study of the molecular mechanisms involved / Tahar Amrouche. Québec: Université Laval, 2005. 175p. 5. Arribas B, Garrido-Mesa N, Perán L, Camuesco D, et al. The immunomodulatory properties of viable Lactobacillus salivarius ssp. salivarius CECT5713 are not restricted to the large intestine. Eur J Nutr, 51:365-374, 2012. 6. Assuma R, Oates T, Cochran D, Amar S, Graves DT. IL-1 and TNF antagonists inhibit the inflammatory response and bone loss in experimental periodontitis. J Immunol, 160:403-409, 1998. 7. Alkaya B, Laleman I, Keceli S, Ozcelik O, Cenk Haytac M, Teughels W. Clinical effects of probiotics containing Bacillus species on gingivitis: a pilot randomized controlled trial. J Periodont Res 2016; doi:10.1111/jre.12415. 8. Badia R, Brufau MT, Guerrero-Zamora AM, Lizardo R, et al. -Galactomannan and Saccharomyces cerevisiae var. boulardii modulate the immune response against Salmonella enterica serovar Typhimurium in porcine intestinal epithelial and dendritic cells. Clin Vaccine Immunol., 19:368-376, 2012. 9. Bass CC. An effective method of personal oral hygiene. J La State Med Soc., 106:57-73, 1954. 10. Berglundh T, Liljenberg B, Tarkowski A, Lindhe J. The presence of local and circulating autoreactive B cells in patients with advanced periodontitis. J. Clin. Periodontol., 29:281-286, 2002. 11. Bien T, Miller WM, Tonigan J. Brief interventions for alcohol problems: a review. Addiction 1993: 88: 315-336. 12. Bogsan CS, Novaes e Brito RR, Palos Mda C, Mortara RA, et al. B-1 cells are pivotal for in vivo inammatory giant cell formation. Int. J. Exp. Pathol., 86:257-265, 2005. 13. Bogsan CSB, Ferreira L, Maldonado C, Perdigon G, et al. Fermented or unfermented milk using Bifidobacterium animalis subsp. lactis HN019: Technological approach determines the probiotic modulation of mucosal cellular immunity. Food Research International, 64, 283-288, 2014. 14. Caglar E, Cildir SK, Ergeneli S, Sandalli N, Twetman S. Salivary mutans streptococci and lactobacilli levels after ingestion of the probiotic bacterium Lactobacillus reuteri ATCC 55730 by straws or tablets. Acta Odontol Scand, 64:314-318, 2006. 15. Caglar E, Kuscu OO, Cildir SK, Kuvvetli SS, Sandalli N. A probiotic lozenge administered medical device and its effect on salivary mutans streptococci and lactobacilli. Int J Paediatr Dent, 18:35-9, 2008. 16. Castillo NA, Perdigón G, de Moreno de Leblanc A. Oral administration of a probiotic Lactobacillus modulates cytokine production and TLR expression improving the immune response against Salmonella enterica serovar Typhimurium infection in mice. BMC Microbiol., 11:177, 2011. 17. Chatterjee A, Bhattacharya H, Kandwal A. Probiotics in periodontal health and disease. J Indian Soc Periodontol., 15:23-28, 2011. 18. Charles CH, Mostler KM, Bartels LL, Mankodi SM. Comparative antiplaque and antigingivitis efficacy of a chlorhexidine and an essential oil mouthrinse: 6-month clinical trial. J Clin Periodontol, 31(10):878-84, 2004. 19. Cochran DL. Inflammation and bone loss in periodontal disease. J Periodontol., 79(Suppl):1569-1576, 2008. 20. Commane D, Hughes R, Shortt C, Rowland I. The potential mechanisms involved in the anti-carcinogenic action of probiotics. Mutat Res., 11;591:276-289, 2005. 21. Cosseau C, Devine DA, Dullaghan E, Gardy JL, et al. The commensal Streptococcus salivarius K12 downregulates the innate immune responses of human epithelial cells and promotes host-microbe homeostasis. Infect. Immun., 76, 4163-4175, 2008. 22. Deng J, Li Y, Zhang J, Yang Q. Co-administration of Bacillus subtilis RJGP16 and Lactobacillus salivarius B1 strongly enhances the intestinal mucosal immunity of piglets. Res Vet Sci, 94:62-68, 2013. 23. DiMatteo MR, Giordani PJ, Lepper HS, Croghan TW. Patient adherence and medical treatment outcomes: a meta-analysis. Med Care 2002: 40: 794-811. 24. Dutzan N, Vernal R, Vaque JP, García-Sesnich J, et al. Interleukin-21 expression and its association with proinflammatory cytokines in untreated chronic periodontitis patients. J Periodontol, 83:948-54, 2012. 25. Eke PI, Dye BA, Wei L, Thornton-Evans GO, et al. Prevalence of periodontitis in adults in the United States: 2009 and 2010. J Dent Res, 91:914-20, 2012. 26. Finamore A, Roselli M, Britti MS, Merendino N, Mengheri E. Lactobacillus rhamnosus GG and Bifidobacterium animalis MB5 induce intestinal but not systemic antigen-specific hyporesponsiveness in ovalbumin-immunized rats. J Nutr, 142:375-81, 2012. 27. Flotra L, Gjermo P, Rolla G, Waerhaug J. Side effects of chlorhexidine mouthwashes. Scand J Dent Res, 79(2):119-25, 1971. 28. Gill HS, Rutherfurd KJ, Cross ML. Dietary probiotic supplementation enhances natural killer cell activity in the elderly: An investigation of age-related immunological changes. J. Clin. Immunol., 21:264-271, 2001. 29. Gill HS, Rutherfurd KJ, Prasad J, Gopal PK. Enhancement of natural and acquired immunity by Lactobacillus rhamnosus (HN001), Lactobacillus acidophilus (HN017) and Bifidobacterium lactis (HN019). Br J Nut., 83:167-76, 2000. 30. Gillor O, Etzion A, Riley MA. The dual role of bacteriocins as anti- and probiotics. Appl. Biotechnol, 81:591-606, 2008. 31. Gruner D, Paris S, Schwendicke F. Probiotics for managing caries and periodontitis: Systematic review and meta- analysis. J Dent, 48:16-25, 2016. 32. Gopal PK, Prasad J, Smart J, Gill HS. In vitro adherence properties of Lactobacillus rhamnosus DR20 and Bifidobacterium lactis DR10 strains and their antagonistic activity against an enterotoxigenic Escherichia coli. Int. J. Food Microbiol., 67:207-216, 2001. 33. Gordon DM. The potential of bacteriocin producing probiotics and associated caveats. Future Microbiol., 4:941-943, 2009. 34. Guidelines for the evaluation of probiotics in food: report of a joint FAO/WHO working group on drafting guidelines for the evaluation of probiotics in food. London: FAO/WHO, 2002.

35. Haffajee AD & Socransky SS. Microbial etiological agents of destructive periodontal diseases. Periodontology 2000, 5:78-111, 1994. 36. Hans M, Hans VM. Toll-like receptors and their dual role in periodontitis: a review. J Oral Sci, 53:263-271, 2011. 37. Hallström H, Lindgren S, Yucel-Lindberg T, Dahlén G, et al. Effect of probiotic lozenges on inflammatory reactions and oral biofilm during experimental gingivitis. Acta Odontol Scand.,71(3-4):828-33, 2013. 38. Harini PM, Anegundi RT. Efficacy of a probiotic and chlorhexidine mouth rinses: a short-term clinical study. J Indian Soc Pedod Prev

Dent., 28(3):179-82, 2010. 39. Ierardo G, Bossù M, Tarantino D, Trinchieri V, et al. The arginine-deiminase enzymatic system on gingivitis: preliminary pediatric study. Ann Stomatol (Rome), 1(1):8-13, 2010. 40. Iniesta M, Herrera D, Montero E, Zurbriggen M, et al. Probiotic effects of orally administered Lactobacillus reuteri-containing tablets on the subgingival and salivary microbiota in patients with gingivitis. A randomized clinical trial. J Clin Periodontol, 39(8):736-44, 2012. 41. nce G, Gürsoy H, pçi D, Cakar G, et al. Clinical and biochemical evaluation of lozenges containing Lactobacillus reuteri as an adjunct to non-surgical periodontal therapy in chronic periodontitis. J Periodontol 2015;86:746-54. 42. Jeon SG, Kayama H, Ueda Y, Takahashi T, et al. Probiotic Bifidobacterium breve induces IL-10-producing Tr1 cells in the colon. PLoS Pathog., 8(5):e1002714., 2012. 43. Junick J, Blaut M. Quantification of human fecal bifidobacterium species by use of quantitative real-time PCR analysis targeting the groEL gene. Appl Environ Microbiol., 78:2613-2622, 2012. 44. Karuppaiah RM, Shankar S, Raj SK, Ramesh K, et al. Evaluation of the efficacy of probiotics in plaque reduction and gingival health maintenance among school children - A Randomized Control Trial. J Int Oral Health, 5(5):33-7, 2013. 45. Kragen H. The treatment of inflammatory affections of the oral mucosa with a lactic acid bacterial culturepreparation. Zahnarztl Welt.10;9(11):306-8. 1954 46. Krasse P, Carlsson B, Dahl C, Paulsson A, et al. Decreased gum bleeding and reduced gingivitis by the probiotic Lactobacillus reuteri. Swed Dent J., 30:55-60, 2006. 47. Kottke T, Battista RN, Degriese G, Brekke M. Attributes of successful smoking cessation interventions in medical practice: a meta-analysis of 30 controlled trials. JAMA, 259: 2882-2889, 1988. 48. Liu C, Zhang ZY, Dong K & Guo XK. Adhesion and immunomodulatory effects of Bifidobacterium lactis HN019 on intestinal epithelial cells INT-407. World J Gastroenterol, 14;16:2283-2290, 2010. 49. Lu Q, Samaranayake LP, Darveau RP, Jin L. Expression of human beta-defensin-3 in gingival epithelia. J Periodontal Res, 40:474-481, 2005. 50. Lee JK, Kim SJ, Ko SH, Ouwehand AC, Ma DS. Modulation of the host response by probiotic Lactobacillus brevis CD2 in experimental gingivitis. Oral Dis, 21(6):705-12, 2015. 51. Loe H, Silness J. Periodontal Disease In Pregnancy. I. Prevalence and Severity. Acta Odontol Scand, 21:533-51, 1963.

52. Loe H, Theilade E, Jensen Sb. Experimental Gingivitis In Man. J Periodontol, 36:177-87, 1965. 53. Mariman R, Kremer B, van Erk M, Lagerweij T, et al. Gene expression profiling identifies mechanisms of protection to recurrent trinitrobenzene sulfonic acid colitis mediated by probiotics. Inflamm Bowel Dis, 18:1424-1433, 2012. 54. Meurman JH. Probiotics: do they have a role in oral medicine and dentistry. Eur J Oral Sci., 113:188-196, 2005. 55. Morales A, Carvajal P, Silva N et al. Clinical effects of Lactobacillus Rhamnosus in non-surgical treatment of chronic periodontitis: a randomized placebo-controlled trial with 1-year follow-up. J Periodontol 2016;4:1-12. 56. Monsarrat P, Blaizot A, Kemoun P, Ravaud P, et al. Clinical research activity in periodontal medicine: a systematic mapping of trial registers. Journal Clin Periodontol, 43: 390-400, 2016. 57. Nadkerny PV, Ravishankar PL, Pramod V, Agarwal LA, Bhandari S. A comparative evaluation of the efficacy of probiotic and chlorhexidine mouthrinses on clinical inflammatory parameters of gingivitis: A randomized controlled clinical study. J Indian Soc Periodontol, 19(6):633-9, 2015. 58. Näse L, Hatakka K, Savilahti E, Saxelin M, et al. Effect of long-term consumption of a probiotic bacterium, Lactobacillus rhamnosus GG, in milk on dental caries and caries risk in children. Caries Res., 35:412-420, 2001. 59. Nishitani Y, Tanoue T, Yamada K, Ishida T, et al. Lactococcus lactis subsp. cremoris FC alleviates symptoms of colitis induced by dextran sulfate sodium in mice. Int Immunopharmacol., 9:1444-1451, 2009. 60. Okamoto K, Fujiya M, Nata T, Ueno N, et al. Competence and sporulation factor derived from Bacillus subtilis improves epithelial cell injury in intestinal inflammation via immunomodulation and cytoprotection. Int J Colorectal Dis, 27:1039-1046, 2012. 61. Ouwehand AC, Salvadori B, Fonden R, Mogensen G, et al. Health effects of probiotics and culture-containing dairy products in humans. Bulletin of the International Dairy Federation, 380:4-19, 2003. 62. Oliveira LFF, Salvador SL, Silva PHF, Furlaneto FAC, Figueiredo L, Casarin R, Ervolino E, Palioto DB, Souza SLS, Taba-Jr M, Novaes-Jr AB, Messora MR. Benefits of Bifidobacterium animalis subsp lactis Probiotic in Experimental Periodontitis. J Periodontol.;88(2):197-208, 2016. 63. World Health Organization. Oral health surveys: basic methods. 4th Ed. Geneva: World Health Organization, 1997. 64. Pancer BA, Kott D, Sugai JV, Panagakos FS, et al. Effects of triclosan on host response and microbial biomarkers during experimental gingivitis. J Clin Periodontol, 43: 435-444, 2016. 65. Petersen PE, Ogawa H. Strengthening the Prevention of Periodontal Disease: The WHO Approach. J Periodontol, 76:2187-2193, 2005. 66. Pulikkotil SJ, Nath S. Effects of curcumin on crevicular levels of IL-1 and CCL28 in experimental gingivitis. Aust Dent J., 60(3):317-27, 2015. 67. Prasad J, Smart JB, Gopal PK, Gill HS. Selection and characterization of Lactobacillus and Bifidobacterium strains for use as probiotics. Int. Dairy J., 8:993-1002, 1998. 68. Philippe D, Heupel E, Blum-Sperisen S, Riedel CU. Treatment with Bifidobacterium bifidum 17 partially protects mice from Th1-driven inflammation in a chemically induced model of colitis. Int J Food Microbiol, 149:45-49, 2011. 69. Pugliese LS, Goncalves TO, Popi AF, Mariano M, et al. B-1 lymphocytes differentiate into functional osteoclast-like cells. Immunobiology, 217:336-344, 2012. 70. Quirynen M, Avontroodt P, Peeters W, Pauwels M, et al. Effect of different chlorhexidine formulations in mouthrinses on de novo plaque formation. J Clin Periodontol, 28(12):1127-36, 2001. 71. Rodrigues DM, Sousa AJ, Johnson-Henry KC, Sherman PM, Gareau MG. Probiotics are effective for the prevention and treatment of Citrobacter rodentium-induced colitis in mice. J Infect Dis, 206:99-109, 2012. 72. SB Brasil 2010: Pesquisa Nacional de Saúde Bucal: resultados principais / Ministério da Saúde. Health Care Secretariat. Health Surveillance Secretariat. - Brasília : Ministry of Health, 2012. 73. Salvi GE & Lang NP. Host response modulation in the management of periodontal diseases. J Clin Periodontol, 32 Suppl 6:108-29, 2005. 74. Samuels N1, Grbic JT, Saffer AJ, Wexler ID, Williams RC. Effect of an herbal mouth rinse in preventing periodontal inflammation in an experimental gingivitis model: a pilot study. Compend Contin Educ Dent, 33(3):204-6, 208-11, 2012. 75. Sazawal S, Dhingra U, Hiremath G, Sarkar A, et al. Effects of bifidobacterium lactis HN019 and prebiotic oligosaccharide added to milk on iron status, anemia, and growth among children 1 to 4 years old. J. pediatr. gastroenterol. nutr., 51:341-346, 2010. 76. Scheres N, Laine ML, de Vries TJ, Everts V, van Winkelhoff AJ. Gingival and periodontal ligament fibroblasts differ in their inflammatory response to viable Porphyromonas gingivalis. J Periodontal Res, 45:262-70, 2010. 77. Shimauchi H, Mayanagi G, Nakaya S, Minamibuchi M, et al. Improvement of periodontal condition by probiotics with Lactobacillus salivarius WB21: a randomized, double- blind, placebo-controlled study. J Clin Periodontol, 35:897-905, 2008. 78. Slawik S1, Staufenbiel I, Schilke R, Nicksch S, et al. Probiotics affect the clinical inflammatory parameters of experimental gingivitis in humans. Eur J Clin Nutr, 65(7):857-63, 2011. 79. Shu Q, Lin H, Rutherfurd KJ, Fenwick SG, et al. Dietary Bifidobacterium lactis (HN019) enhances resistance to oral Salmonella typhimurium infection in mice. Microbiol. Immunol., 44:213- 222, 2000. 80. Sliepen I, Van Damme J, Van Essche M, Loozen G, et al. Microbial interactions influence inflammatory host cell responses. J Dent Res, 88:1026-1030, 2009. 81. Socransky SS, Haffajee AD, Smith C, Martin L, et al. Use of checkerboard DNA-DNA hybridization to study complex microbial ecosystems. Oral Microbiol Immunol, 19:352-62, 2004. 82. Sookkhee S, Chulasiri M, Prachyabrued W. Lactic acid bacteria from healthy oral cavity of Thai volunteers: inhibition of oral pathogens. J. Appl. Microbiol., 90:172-179, 2001. 83. Staab B, Eick S, Knöfler G, Jentsch H. The influence of a probiotic milk drink on the development of gingivitis: a pilot study. J Clin Periodontol, 36:850-856, 2009. 84. Stamatova I & Meurman JH. Probiotics and periodontal disease. Periodontol 2000, 51:141-151, 2009. 85. Stelin S, Ramakrishan H, Talwar A, Arun KV, Kumar TS. Immunohistological analysis of CD1a langerhans cells and CD57 natural killer cells in healthy and diseased human gingival tissue: A comparative study. J Indian Soc Periodontol, 13:150-154, 2009. 86. Teughels W, Newman MG, Coucke W, Haffajee AD, et al. Guiding periodontal pocket recolonization: a proof of concept. J Dent Res, 86:1078-1082, 2007. 87. Teughels W, Van Essche M, Sliepen I & Quirynen M. Probiotics and oral health. Periodontol 2000, 48:111-1147, 2008. 88. Tekce M, Ince G, Gursoy H, et al. Clinical and microbiological effects of probiotic lozenges in the treatment of chronic periodontitis: a 1-year follow-up study. J Clin Periodontol 2015;42:363-72. 89. Tonetti MS, Chapple IL. Working Group 3 of Seventh European Workshop on Periodontology. Biological approaches to the development of novel periodontal therapies-consensus of the Seventh European Workshop on Periodontology. J Clin Periodontol, 38 Suppl 11:114-118, 2011. 90. Tonetti MS, Eickholz P, Loos BG, Papapanou P, et al. Principles in prevention of periodontal diseases: Consensus report of group 1 of the 11th European Workshop on Periodontology on effective prevention of periodontal and peri-implant diseases. J Clin Periodontol, 42 Suppl 16:S5-11, 2015. 91. Tsubura S, Mizunuma H, Ishikawa S, Oyake I, et al. The effect of Baccillus subtilis Mouth rinsing in patients with periodontitis. Eur J Clin Microbiol Infect Dis, 28:1353-1356, 2009. 92. Turesky S, Gilmore ND, Glickman I. Reduced plaque formation by the chloromethyl analogue of victamine C. J Periodontol., 41(1):41-3, 1970. 93. Twetman S, Derawi B, Keller M, Ekstrand K, et al. Short-term effect of chewing gums containing

probiotic Lactobacillus reuteri on the levels of inflammatory mediators in gingival crevicular fluid. Acta Odontol Scand, 67:19-24, 2009. 94. van der Ouderaa EJG: Anti-plaque agents. Rationale and prospects for prevention of gingivitis and periodontal disease. J Clin Periodontol, 18: 447-454, 1991. 95. Vivekananda MR, Vandana KL, Bhat KG. Effect of the probiotic Lactobacilli reuteri (Prodentis) in the management of periodontal disease: a preliminary randomized clinical trial. J Oral Microbiol. 2:5344, 2010. 96. Zhang G, Chen R, Rudney JD. Streptococcus cristatus attenuates Fusobacterium nucleatum-induced interleukin-8 expression in oral epithelial cells. J. Periodontal. Res., 43, 408-416, 2008. 97. Zhou JS & Gill HS. Immunostimulatory probiotic Lactobacillus rhamnosus HN001 and Bifidobacterium lactis HN019 do not induce pathological inflammation in mouse model of experimental autoimmune thyroiditis. Int. J. Food Microbiol., 103:97-104, 2005. 98. Zhu D, Chen X, Wu J, Ju Y, et al. Effect of perioperative intestinal probiotics on intestinal flora and immune function in patients with colorectal cancer. Nan Fang Yi Ke Da Xue Xue Bao, 32:1190-1193, 2012.

Upload Documents File

Attachments:

Type	Archive
Proof of Receipt	PB_COMPROVANTE_RECEPÇÃO_897890.pdf
Cover Sheet	ROSE_SHEET1.pdf
Statement of Institution and Infrastructure	aut_infrDCTMF.pdf
ICF / Terms of Assent / Justification for Absence	TCLE.docx
Others	Questionnaire.docx
Statement of Institution and Infrastructure	aut_infrC.pdf
Others	dec_part_pesq.doc
Others	Flavia_Formulario_para_EMENDA_Projeto_Gengivite.pdf
Detailed Project / Investigator Brochure	RESEARCH_DRAFT.pdf
Proof of Receipt	PB_COMPROVANTE_RECEPCAO_964740.pdf

Finish

Keep the entire research project confidential: Yes

Term: 2 years

Justification for the amendment:

All the clinical data of the patients in this research project has already been collected and the clinical procedures of the study have been completed. Samples of dental biofilm were collected for microbiological analysis. The microbiological analysis that had been submitted to the Research Ethics Committee aimed to verify the microbiological composition of the biofilm by means of checkerboard DNA-DNA hybridization and the absolute quantification of Bifidobacterium animalis subsp. lactis HN019 in the biofilm by qPCR. However, the opportunity arose to carry out a more complete microbiological analysis of the biofilm samples using next-generation sequencing technology. I declare that this will not influence patient care or the collection of their samples, which have already been carried out.
