# Supplementary material for: Microbiological profile of patients with generalized gingivitis undergoing periodontal therapy and administration of Bifidobacterium animalis subsp. lactis HN019: A randomized clinical trial
Source: PLoS One. 2024 Nov 11;19(11):e0310529. doi: 10.1371/journal.pone.0310529 (PMC11554181; doi:10.1371/journal.pone.0310529)
Supplement: S3 Appendix — Original version in Portuguese. (PDF) [file pone.0310529.s005.pdf]

S3 Appendix. Informed consent form. Original version in Portuguese.

Termo de Consentimento Livre e Esclarecido

Nós, Prof[a]. Dra. Flávia Aparecida Chaves Furlaneto, Prof. Dr. Michel Reis Messora, Pedro Henrique Felix Silva e Renata Silva Cardoso convidamos você, ____, a participar da pesquisa “Efeitos da terapia probiótica no desenvolvimento da gengivite: estudo do perfil clínico, microbiológico e imunológico da resposta do hospedeiro.”

Esta pesquisa tem como objetivo avaliar os efeitos da administração oral da cepa probiótica *B. lactis* HN019 no tratamento da gengivite. Probióticos podem ser definidos como microrganismos vivos, principalmente bactérias, seguros para o consumo e capazes de produzirem efeitos benéficos para a saúde quando ingeridos em quantidades suficientes. Antes do estudo começar, você receberá instruções específicas de higiene oral e uma limpeza em todos os seus dentes. Serão feitas radiografias. Logo em seguida, você será encaixado em um dos seguintes grupos experimentais: controle ou teste, sendo que em ambos os grupos você receberá acompanhamento clínico e 56 pastilhas que deverão ser consumidas diariamente. Você não saberá em qual grupo foi alocado durante todo o estudo. Apenas as pastilhas do grupo teste apresentarão bactérias vivas (10^9 unidades formadoras de colônias de *Bifidobacterium animalis subsp. Lactis* HN019 por grama), as quais são seguras para o consumo e podem promover equilíbrio de sua flora intestinal.

Após a realização do exame clínico, você deverá consumir a pastilha uma vez ao dia durante 8 semanas, dissolvendo-a na boca antes de dormir. Ao longo do estudo, serão coletadas algumas informações clínicas (índice de placa, índice gengival, índice de sangramento gengival, profundidade clínica de sondagem, nível clínico de inserção, sangramento à sondagem), microbiológicas (coleta de bactérias aderidas nas coroas e raízes de seus dentes) e imunológicas (coleta do líquido existente entre a gengiva e a raiz de seus dentes) com a finalidade de documentação e avaliação dos resultados obtidos. Você será acompanhado(a) por um período de 56 dias.

Os procedimentos que poderão lhe causar incômodo são: exames periodontais (procedimentos de sondagem, coleta de placa e coleta de líquido existente entre a gengiva e raiz dos dentes) que serão realizados em sua boca e radiografias. Todos os procedimentos necessários para minimização dos riscos existentes serão cuidadosamente observados. O consumo do probiótico poderá provocar gases intestinais temporários e mínimo desconforto.

Todos os dados relacionados a você serão confidenciais e sua identidade será mantida em sigilo. A divulgação dos resultados será realizada preservando sempre a sua identidade. Você será identificado(a) por um número ou apenas por suas iniciais, não tendo seu nome ou dados que lhe possam identificar revelados.

A sua participação não é obrigatória e você poderá desistir a qualquer momento, retirando o seu consentimento. A não participação neste projeto de pesquisa não trará nenhum prejuízo em sua relação com o pesquisador ou com a Faculdade de Odontologia de Ribeirão Preto – USP.

Você terá garantido o ressarcimento de despesas decorrentes da participação no estudo, tais como transporte urbano e alimentação nos dias em que for necessária sua presença para consultas ou exames.

O presente Termo, confeccionado em duas vias de igual teor, será assinado na última página e rubricado nas demais páginas pelos pesquisadores e pelo participante da pesquisa. Você receberá uma via deste termo, com o endereço e telefone do pesquisador e da secretaria do Comitê de Ética em Pesquisa (CEP) da FORP/USP. Caso tenha alguma dúvida, você poderá entrar em contato com a secretaria do CEP pelo telefone (16) 3315-0493 das 13h30 às 17h30, de segunda a sexta-feira (exceto feriados e pontos facultativos).

____
Flávia Aparecida Chaves Furlaneto, DDS, PhD

Av. do Cafe s/n, 14040-904, Ribeirao Preto-SP, Brazil
Department of Oral & Maxillofacial Surgery and Periodontology
Ribeirao Preto School of Dentistry – University of Sao Paulo
+55 (16) 3315-4140

____
Michel Reis Messora, DDS, PhD

Av. do Cafe s/n, 14040-904, Ribeirao Preto-SP, Brazil
Department of Oral & Maxillofacial Surgery and Periodontology
Ribeirao Preto School of Dentistry – University of Sao Paulo
+55 (16) 3315-4140

____

Pedro Henrique Felix Silva, DDS
Av. do Cafe s/n, 14040-904, Ribeirao Preto-SP, Brazil
Department of Oral & Maxillofacial Surgery and Periodontology
Ribeirao Preto School of Dentistry – University of Sao Paulo
+55 (16) 3315-4092

____

Renata Silva Cardoso, DDS
Av. do Cafe s/n, 14040-904, Ribeirao Preto-SP, Brazil
Department of Oral & Maxillofacial Surgery and Periodontology
Ribeirao Preto School of Dentistry – University of Sao Paulo
+55 (16) 3315-4092

Declaro que entendi os objetivos, riscos e benefícios de minha participação na pesquisa e concordo em participar.

____

Sujeito da pesquisa

RG...
Telefone...
Endereço...
....
....
Data:____/____/____.
