# Supplementary material for: Microbiological profile of patients with generalized gingivitis undergoing periodontal therapy and administration of Bifidobacterium animalis subsp. lactis HN019: A randomized clinical trial
Source: PLoS One. 2024 Nov 11;19(11):e0310529. doi: 10.1371/journal.pone.0310529 (PMC11554181; doi:10.1371/journal.pone.0310529)
Supplement: S4 Appendix — English version. (PDF) [file pone.0310529.s006.pdf]

S4 Appendix. Informed consent form. English version.

Informed Consent Form

We, Prof. Dr. Flávia Aparecida Chaves Furlaneto, Prof. Dr. Michel Reis Messora, Pedro Henrique Felix Silva and Renata Silva Cardoso invite you, ____, to participatein the research "Effects of probiotic therapy on the development of gingivitis: study of the clinical, microbiological and immunological profile of the host response."

This research aims to evaluate the effects of oral administration of the probiotic strain *B. lactis* HN019 in the treatment of gingivitis. Probiotics can be defined as live microorganisms, mainly bacteria, that are safe to consume and capable of producing beneficial health effects when ingested in sufficient quantities. Before the study begins, you will receive specific oral hygiene instructions and a cleaning of all your teeth. X-rays will be taken. You will then be placed in one of the following experimental groups: control or test, in both of which you will receive clinical follow-up and 56 tablets to be consumed daily. You will not know which group you have been allocated to throughout the study. Only the tablets in the test group will contain live bacteria (10^9 colony-forming units of *Bifidobacterium animalis subsp. Lactis* HN019 per gram), which are safe to consume and can promote a balance in your intestinal flora.

After the clinical examination, you will have to consume the tablet once a day for 8 weeks, dissolving it in your mouth before going to bed. Throughout the study, some clinical (plaque index, gingival index, gingival bleeding index, clinical probing depth, clinical insertion level, bleeding on probing), microbiological (collection of bacteria adhered to the crowns and roots of your teeth) and immunological (collection of the fluid between the gums and the roots of your teeth) information will be collected in order to document and evaluate the results obtained. You will be monitored for a period of 56 days.

The procedures that may cause you discomfort are: periodontal examinations (probing procedures, plaque collection and collection of fluid between the gums and the roots of the teeth) that will be carried out in your mouth and X-rays. All the procedures necessary to minimize the existing risks will be carefully observed. Consumption of the probiotic may cause temporary intestinal gas and minimal discomfort.

All data relating to you will be kept confidential and your identity will be kept secret. The results will be published while preserving your identity. You will be identified by a number or only by your initials, and your name or any identifying information will not be revealed.

Your participation is not compulsory and you may withdraw your consent at any time. Non-participation in this research project will not harm your relationship with the researcher or with the Faculty of Dentistry of Ribeirão Preto - USP.

You will be guaranteed reimbursement of expenses incurred as a result of participating in the study, such as urban transportation and meals on days when you are required to be present for consultations or examinations.

This Agreement, drawn up in two copies of equal content, will be signed on the last page and initialed on the other pages by the researchers and the research participant. You will receive a copy of this form, with the address and telephone number of the researcher and the office of the Research Ethics Committee (CEP) of FORP/USP. If you have any questions, you can contact the CEP secretariat on (16) 3315-0493 from 1:30 p.m. to 5:30 p.m., Monday to Friday (except public holidays).

____

Flávia Aparecida Chaves Furlaneto, DDS, PhD

Av. do Cafe s/n, 14040-904, Ribeirao Preto-SP, Brazil
Department of Oral & Maxillofacial Surgery and Periodontology
Ribeirao Preto School of Dentistry - University of Sao Paulo
+55 (16) 3315-4140

____

Michel Reis Messora, DDS, PhD

Av. do Cafe s/n, 14040-904, Ribeirao Preto-SP, Brazil
Department of Oral & Maxillofacial Surgery and Periodontology
Ribeirao Preto School of Dentistry - University of Sao Paulo
+55 (16) 3315-4140

____

Pedro Henrique Felix Silva, DDS
Av. do Cafe s/n, 14040-904, Ribeirao Preto-SP, Brazil
Department of Oral & Maxillofacial Surgery and PeriodontologyRibeirao Preto School of Dentistry - University of Sao Paulo
+55 (16) 3315-4092

____

Renata Silva Cardoso, DDS
Av. do Cafe s/n, 14040-904, Ribeirao Preto-SP, Brazil
Department of Oral & Maxillofacial Surgery and PeriodontologyRibeirao Preto School of Dentistry - University of Sao Paulo
+55 (16) 3315-4092

I hereby declare that I have understood the objectives, risks, and benefits of my participation in the research and agree to participate.

____
Research subject

RG...
Phone...
Address..
....
....
Date:____/____/____.
