# Supplementary material for: Microbiological profile of patients with generalized gingivitis undergoing periodontal therapy and administration of Bifidobacterium animalis subsp. lactis HN019: A randomized clinical trial
Source: PLoS One. 2024 Nov 11;19(11):e0310529. doi: 10.1371/journal.pone.0310529 (PMC11554181; doi:10.1371/journal.pone.0310529)
Supplement: S1 Text — (DOCX) [file pone.0310529.s007.docx]

**S1 Text. Bacterial DNA Extraction, 16S rRNA Gene Sequencing and Bioinformatics Analysis.**

Microbial DNA was obtained from 96 biofilm samples using a commercial QIAamp DNA mini-Kit (QIAGEN, Düsseldorf, Germany). Next generation sequencing targeting the V1-V2 hypervariable region of the 16S rRNA gene from the samples was carried out at NGS Soluções Genômicas (Piracicaba, SP, Brazil). The Illumina recommendations for library preparation were followed. Libraries were prepared using 2uL of stock DNA, 0.2uM of each primer, 0.8X Ultra Mix 2X PCRBio (PCR Biosystems, Wayne, PA, USA) and ultrapure water to make up a final volume of 25uL. The PCR reaction was performed under the following conditions: 95ºC for 3 minutes, 25 cycles of 95ºC for 30 seconds, 62ºC for 30 seconds, 72ºC for 30 seconds and the final extension of 72ºC for 5 minutes. PCR products were purified with the Beads AMPureXP kit (Beckman Coulter. Brea., CA, USA). After purification, the Illumina adapters were ligated in a PCR reaction. This reaction was also purified. The ligation product of the adapters was normalized to the same concentration. After normalization, a pool was made with the same volume of each sample and subsequent quantification by qPCR, to validate and determine the final concentration of the pool. KAPA Library Quantification Kit for Illumina Platforms (KAPA Biosystems. Woburn. MA) was used in the qPCR reactions. A single-read sequencing of 250 nucleotides was performed using the MiSeq Illumina platform (Illumina. San Diego. CA) 27F primer (5′-TCGTCGGCAGCGTCAGATGTGTATAAGAGACAGAGAGTTTGATCMTGGCTCAG-3').

Demultiplexed sequences with quality without barcodes and linker-primers were imported into the Quantitative Insights into Microbial Ecology (QIIME) 2 package version 2021.11 [1]. Reads were filtered by removal of low quality regions and chimeric sequences and denoise and correction of amplicon sequence data using the q2-dada2 plugin (DADA2). After quality filtering, data was summarized and tabulated.

The ASVs were then taxonomically assigned to species-level based on Human Oral Microbiome Database (eHOMD) 16S rRNA RefSeq Version 15.22 [2] using the sklearn-based taxonomy classifier algorithm. The extended eHOMD places 16S rRNA gene reference sequences for each human microbial taxon (HMT) on a phylogenetic tree (https://v2.homd.org/ftp/phylogenetic_trees/refseq/current/eHOMD_16S_rRNA_RefSeq.svg), so unnamed or uncultivated species are defined based on sequence identity and added to the phylogeny using a provisional naming scheme that permits taxonomic assignment for cross-study comparison. Furthermore, each HMT in eHOMD is represented by one to six highly curated eHOMD references to account for intraspecies variability across different strains and dissimilar 16S rRNA genomic copies within individual strains [3], strengthening taxonomy classification accuracy.

**References**

1 Bolyen E, Rideout JR, Dillon MR, Bokulich NA, Abnet CC, Al-Ghalith GA, et al. Reproducible, interactive, scalable and extensible microbiome data science using QIIME 2. Nat Biotechnol. 2019 Aug;37(8):852–7.

2 Chen T. Yu WH. Izard J. Baranova OV. Lakshmanan A. Dewhirst FE. The Human Oral Microbiome Database: a web accessible resource for investigating oral microbe taxonomic and genomic information. Database (Oxford). 2010 Jul 6;2010:baq013.

3 Escapa IF. Chen T. Huang Y. Gajare P. Dewhirst FE. Lemon KP. New Insights into Human Nostril Microbiome from the Expanded Human Oral Microbiome Database (eHOMD): a Resource for the Microbiome of the Human Aerodigestive Tract. mSystems. 2018 Dec 4;3(6):e00187-18.
