# Supplementary material for: Microbiological profile of patients with generalized gingivitis undergoing periodontal therapy and administration of Bifidobacterium animalis subsp. lactis HN019: A randomized clinical trial
Source: PLoS One. 2024 Nov 11;19(11):e0310529. doi: 10.1371/journal.pone.0310529 (PMC11554181; doi:10.1371/journal.pone.0310529)
Supplement: S1 Table — * represents significant differences within the placebo group at different time-points (Wilcoxon test. p<0.05). ** represents significant differences within the probiotic group at different time-points (Wilcoxon test. p<0.05). *** represents significant differences between groups post-therapy (Mann-Whitney. p<0.05). Post-therapy: 8 weeks after treatment. (DOCX) [file pone.0310529.s008.docx]

**S1 Table. Relative abundance of bacterial genera in the gingival biofilm of gingivitis patients pre- and post-therapies.**

| **Genera detected at ≥ 1% at baseline** | **Placebo Group (n=28)** | | | **Probiotic Group (n=25)** | |
| --- | --- | --- | --- | --- | --- |
|  | **Baseline** | | **Post-therapy** | **Baseline** | **Post-therapy** |
| ***Streptococcus*** | 9.73% | 10.07% | | 12.32% | 9.21% |
| ***Leptotrichia*** | 8.94% | 9.08% | | 7.55% | 7.32% |
| ***Actinomyces*** | 8.90% | 11.14% | | 12.40% | 12.56% |
| ***Fusobacterium*** | 8.32% | 6.63% | | 6.40% | 6.67% |
| ***Prevotella*** | 6.80% | 5.75% | | 5.14% | 5.67% |
| ***Veillonella*** | 5.91% | 6.35% | | 6.77% | 6.51% |
| ***Corynebacterium*** | 5.13% | 6.85% | | 7.54% | 7.64% |
| ***Neisseria*** | 4.56% | 5.09% | | 3.22% | 2.70% |
| ***Saccharibacteria* (TM7) [G-1]** | 4.19% | 3.54% | | 4.97% | 6.09% |
| ***Rothia*** | 3.83% | 1.65% | | 3.14% | 2.67% |
| ***Selenomonas*** | 3.39% | 3.08% | | 2.20% | 2.82% |
| ***Capnocytophaga*** | 3.30% | 3.64% | | 3.11% | 3.80% |
| ***Lautropia*** | 3.02% | 2.66% | | 4.29% | 3.84% |
| ***Arachnia*** | 2.80% | 1.99% | | 1.63% | 1.74% |
| ***Porphyromonas*** | 1.21% | 1.33% | | 1.31% | 1.51% |
| ***Treponema*** | 1.14% | 1.18% | | 0.98% | 1.10% |
| ***Schaalia**** | 1.30% | 1.87% | | 2.45% | 1.54% |
| **Genera detected at ≤ 1% at baseline that changed significantly over time** | **Baseline** | **Post-therapy** | | **Baseline** | **Post-therapy** |
| *Granulicatella*** | 0.70% | 0.75% | | 1.07% | 0.64% |
| *Tannerella** | 0.69% | 0.40% | | 0.41% | 0.53% |
| *Saccharibacteria* (TM7) [G-2]**** | 0.43% | 0.14% | | 0.07% | 0.23% |
| *Catonella*** | 0.42% | 0.30% | | 0.24% | 0.44% |
| *Peptostreptococcaceae* [XI][G-7]***** | 0.45% | 0.50% | | 0.33% | 0.63% |
| *Peptostreptococcus**** | 0.15% | 0.11% | | 0.13% | 0.26% |
| *Propionibacterium**** | 0.002% | 0.00% | | 0.29% | 0.15% |

* represents significant differences within the placebo group at different time-points (Wilcoxon test. p<0.05). ** represents significant differences within the probiotic group at different time-points (Wilcoxon test. p<0.05). *** represents significant differences between groups post-therapy (Mann-Whitney. p<0.05). Post-therapy: 8 weeks after treatment.
