# Supplementary material for: Microbiological profile of patients with generalized gingivitis undergoing periodontal therapy and administration of Bifidobacterium animalis subsp. lactis HN019: A randomized clinical trial
Source: PLoS One. 2024 Nov 11;19(11):e0310529. doi: 10.1371/journal.pone.0310529 (PMC11554181; doi:10.1371/journal.pone.0310529)
Supplement: S2 Table — *p< 0.05; **p<0.01. Numbers refer to the Spearman’s correlation coefficient (rho). T8: 8 weeks after initial therapy. (DOCX) [file pone.0310529.s009.docx]

**S2 Table. Correlation between oral genera and % of reduction in BOMP at both therapeutic groups.**

| **Change in relative abundance post-therapy (T8)** | **% of reduction in BOMP** |
| --- | --- |
| **Placebo Group** | |
| *Bergeyella* | 0.637** |
| *Kingella* | 0.438* |
| **Probiotic Group** | |
| *Bacteroidetes* [G-3] | 0.487* |
| *Corynebacterium* | -0.635** |
| *Neisseria* | 0.470* |
| *Peptostreptococcaceae* [XI][G-1] | 0.512* |
| *Peptostreptococcaceae* [XI][G-5] | 0.483* |
| *Saccharibacteria* (TM7) [G-8] | 0.571* |

*p< 0.05; **p<0.01. Numbers refer to the Spearman's correlation coefficient (rho). T8: 8 weeks after initial therapy.
