# Supplementary material for: Microbiological profile of patients with generalized gingivitis undergoing periodontal therapy and administration of Bifidobacterium animalis subsp. lactis HN019: A randomized clinical trial
Source: PLoS One. 2024 Nov 11;19(11):e0310529. doi: 10.1371/journal.pone.0310529 (PMC11554181; doi:10.1371/journal.pone.0310529)
Supplement: S3 Table — Mean abundance of oral species detected at ≥ 0.1% for all samples at baseline. * Refers to taxa that differed between therapeutic groups at baseline or at 8 weeks post-therapy (Mann-Whitney test. p < 0.05). Green shading represents significant differences between baseline and post-therapy within each group (Wilcoxon test. p < 0.05). (DOCX) [file pone.0310529.s010.docx]

**S3 Table. Relative abundance of bacterial taxa at species level.** Mean abundance of oral species detected at ≥ 0.1% for all samples at baseline.

| **Oral species at abundance ≥ 0.01%** | **Placebo Group (n=28)** | | **Probiotic Group (n=25)** | |
| --- | --- | --- | --- | --- |
|  | **Baseline** | **Post-therapy** | **Baseline** | **Post-therapy** |
| *Actinomyces sp.* HMT-169 | **0.86%*** | 1.79% | **2.26%*** | 3.25% |
| *Capnocytophaga gingivalis* | 0.20% | 0.33% | 0.16% | 0.42% |
| *Capnocytophaga sp.* HMT-326 | 0.06% | **0.06%*** | 0.16% | **0.18%*** |
| *Capnocytophaga sp.* HMT-380 | 0.13% | 0.11% | 0.07% | 0.16% |
| *Catonella morbi* | 0.41% | 0.28% | 0.22% | 0.37% |
| *Granulicatella adiacens* | 0.66% | 0.73% | 1.06% | 0.64% |
| *Peptostreptococcaceae* [XI][G-7] *[Eubacterium] yurii* subsps. *yurii margaretiae* | 0.39% | **0.47%*** | 0.32% | **0.61%*** |
| *Peptostreptococcus stomatis* | 0.15% | **0.11%*** | 0.13% | **0.26%*** |
| *Porphyromonas catoniae* | 0.25% | 0.28% | 0.28% | 0.62% |
| *Prevotella maculosa* | 0.15% | **0.08*** | 0.17% | **0.19*** |
| *Prevotella sp.* HMT-472 | **0.44%*** | 0.46% | **0.08%*** | 0.21% |
| *Saccharibacteria (TM7) [G-1] bacterium* HMT-348 | **0.49%*** | 0.52 | **0.61%*** | 0.72 |
| *Saccharibacteria (TM7) [G-1] bacterium* HMT-349 | 0.97% | 0.71% | 2.10% | 2.61% |
| *Saccharibacteria (TM7) [G-1] bacterium* HMT-488 | 0.69% | 0.90% | 0.16% | 0.38% |
| *Saccharibacteria (TM7) [G-2] bacterium* HMT-350 | 0.43% | 0.14% | 0.07% | 0.23% |
| *Schaalia sp.* HMT-180 | 0.69% | 0.86% | 0.55% | 0.45% |
| *Solobacterium moorei* | 0.31% | 0.19% | 0.10% | 0.19% |
| *Streptococcus cristatus clade 578* | 0.32% | **0.50%*** | 0.20% | **0.11%*** |
| *Streptococcus intermedius* | 0.47% | 0.37% | 0.37% | 0.12% |

* Refers to taxa that differed between therapeutic groups at baseline or at 8 weeks post-therapy (Mann-Whitney test. p < 0.05). Green shading represents significant differences between baseline and post-therapy within each group (Wilcoxon test. p < 0.05).
