# Supplementary material for: Microbiological profile of patients with generalized gingivitis undergoing periodontal therapy and administration of Bifidobacterium animalis subsp. lactis HN019: A randomized clinical trial
Source: PLoS One. 2024 Nov 11;19(11):e0310529. doi: 10.1371/journal.pone.0310529 (PMC11554181; doi:10.1371/journal.pone.0310529)
Supplement: S4 Table — Dependent variable: reduction on bleeding on marginal probing (BOMP). Changes in the abundance of 441 oral species were entered as predictor variables in the analysis using the stepwise method. The model that met all assumptions was obtained at step 6. Decrease in abundance of Treponema sp. HMT-231 after treatment was a good predictor of high reduction in gingival bleeding. Increase in abundances of Bergeyella sp. HMT-322 and Schaalia sp. HMT-178 after treatment were good predictors of high reduction in gingival bleeding. (DOCX) [file pone.0310529.s011.docx]

**S4 Table. Multiple linear regression for prediction of reduction in BOMP based on changes in relative abundance of oral species/phylotypes post-therapy in the Placebo group.**

|  | **Unstandardized Coefficients** | | **Standardized Coefficients** | **t** | **Sig.** | **95.0% Confidence Interval for B** | | **Collinearity Statistics** | |
| --- | --- | --- | --- | --- | --- | --- | --- | --- | --- |
|  | **B** | **Std. Error** | **Beta** |  |  | **Lower Bound** | **Upper Bound** | **Tolerance** | **VIF** |
| **(Constant)** | .158 | .023 |  | 6.865 | .000 | .109 | .207 |  |  |
| ***Treponema sp._*HMT-231** | -.243 | .038 | **-.460** | -6.392 | .000 | -.324 | -.163 | .873 | 1.146 |
| ***Actinomyces oris*** | -.042 | .013 | -.226 | -3.126 | .007 | -.071 | -.014 | .865 | 1.156 |
| ***Bergeyella sp._*HMT-322** | .048 | .007 | **.503** | 6.723 | .000 | .033 | .064 | .809 | 1.236 |
| ***Schaalia sp._*HMT-178** | .074 | .015 | **.355** | 5.051 | .000 | .043 | .105 | .918 | 1.090 |
| ***Streptococcus infantis_clade_638*** | -.575 | .138 | -.292 | -4.169 | .001 | -.868 | -.283 | .923 | 1.084 |
| ***Burkholderia cepacia*** | -.224 | .061 | -.251 | -3.659 | .002 | -.354 | -.094 | .957 | 1.045 |
| **Model summary** | | | | **ANOVA** | | | | | |
| **R** | **R Square** | **Adjusted R Square** | **Std. Error of the Estimate** |  | **Sum of Squares** | **df** | **Mean Square** | **F** | **Sig.** |
| 0.963 | .928 | .901 | .08094 | **Regression** | 1.344 | 6 | .224 | 34.194 | .000 |
|  |  |  |  | **Residual** | .105 | 16 | .007 |  |  |
|  |  |  |  | **Total** | 1.449 | 22 |  |  |  |

Dependent variable: reduction on bleeding on marginal probing (BOMP). Changes in the abundance of 441 oral species were entered as predictor variables in the analysis using the stepwise method. The model that met all assumptions was obtained at step 6. Decrease in abundance of *Treponema* sp. HMT-231 after treatment was a good predictor of high reduction in gingival bleeding. Increase in abundances of *Bergeyella sp.* HMT-322 and *Schaalia sp.* HMT-178 after treatment were good predictors of high reduction in gingival bleeding.
