# Supplementary material for: Microbiological profile of patients with generalized gingivitis undergoing periodontal therapy and administration of Bifidobacterium animalis subsp. lactis HN019: A randomized clinical trial
Source: PLoS One. 2024 Nov 11;19(11):e0310529. doi: 10.1371/journal.pone.0310529 (PMC11554181; doi:10.1371/journal.pone.0310529)
Supplement: S5 Table — Dependent variable: reduction on bleeding on marginal probing (BOMP). Changes in the abundance of 441 oral species were entered as predictor variables in the analysis using the stepwise method. The model that met all assumptions was obtained at step 6. Decrease in abundances of Actinomyces sp. HMT-175 and Leptotrichia HMT-498 after therapy were good predictors of high reduction in gingival bleeding. (DOCX) [file pone.0310529.s012.docx]

**S5 Table. Multiple linear regression for prediction of reduction in BOMP based on changes in relative abundance of oral species/phylotypes post-therapy in the Probiotic group.**

|  | **Unstandardized Coefficients** | | **Standardized Coefficients** | **t** | **Sig.** | **95.0% Confidence Interval for B** | | **Collinearity Statistics** | |
| --- | --- | --- | --- | --- | --- | --- | --- | --- | --- |
|  | **B** | **Std. Error** | **Beta** |  |  | **Lower Bound** | **Upper Bound** | **Tolerance** | **VIF** |
| **(Constant)** | .807 | .012 |  | 70.005 | .000 | .782 | .833 |  |  |
| ***Actinomyces sp._*HMT-175** | -.041 | .002 | **-.910** | -22.887 | .000 | -.045 | -.037 | .949 | 1.054 |
| ***Leptotrichia _*HMT-498** | -.005 | .000 | **-.422** | -10.211 | .000 | -.006 | -.004 | .880 | 1.136 |
| ***Fusobacterium sp._*HMT-204** | -.047 | .008 | -.230 | -5.711 | .000 | -.066 | -.029 | .927 | 1.079 |
| ***Mitsuokella sp._*HMT-131** | -.031 | .010 | -.135 | -3.236 | .008 | -.052 | -.010 | .862 | 1.160 |
| ***Prevotella oralis*** | .030 | .008 | .142 | 3.581 | .004 | .012 | .049 | .955 | 1.047 |
| ***Actinomyces naeslundii*** | -.016 | .005 | -.121 | -3.014 | .012 | -.028 | -.004 | .938 | 1.066 |
| **Model summary** | | | | **ANOVA** | | | | | |
| **R** | **R Square** | **Adjusted R Square** | **Std. Error of the Estimate** |  | **Sum of Squares** | **df** | **Mean Square** | **F** | **Sig.** |
| .992 | .983 | .974 | .03546 | **Regression** | .824 | 6 | .137 | 109.178 | .000g |
|  |  |  |  | **Residual** | .014 | 11 | .001 |  |  |
|  |  |  |  | **Total** | .837 | 17 |  |  |  |

Dependent variable: reduction on bleeding on marginal probing (BOMP). Changes in the abundance of 441 oral species were entered as predictor variables in the analysis using the stepwise method. The model that met all assumptions was obtained at step 6. Decrease in abundances of *Actinomyces* sp. HMT-175 and *Leptotrichia* HMT-498 after therapy were good predictors of high reduction in gingival bleeding.
